# An integrated approach for studying exposure, metabolism, and disposition of traditional Chinese medicine using PATBS and MDRB tools: a case study of semen *Armeniacae Amarum*

**DOI:** 10.1186/s13020-024-01031-8

**Published:** 2024-11-14

**Authors:** Dandan Zhang, Junyu Zhang, Simian Chen, Hairong Zhang, Yuexin Yang, Shan Jiang, Yun Hong, Mingshe Zhu, Qiang Xie, Caisheng Wu

**Affiliations:** 1 Fujian Provincial Key Laboratory of Innovative Drug Target Research and State Key Laboratory of Cell Stress Biology, School of Pharmaceutical Sciences, Xiamen University, Xiamen, 361102 Fujian China; 2 Xiamen Key Laboratory for Clinical Efficacy and Evidence-Based Research of Traditional Chinese Medicine, Xiamen University, Xiamen, 361005 China; 3 Department of Cardiology, School of Medicine, The First Affiliated Hospital of Xiamen University, Xiamen University, Xiamen, 361005 China; 4 Mass Defect Technologies, Princeton, NJ USA

**Keywords:** TCM, Semen *Armeniacae Amarum*, Intelligent MS data processing technology, PATBS, ADME

## Abstract

**Background:**

Deciphering the in vivo processes of traditional Chinese medicine (TCM) is crucial for identifying new pharmacodynamic substances and new drugs. Due to the complexity and diversity of components, investigating the exposure, metabolism, and disposition remains a major challenge in TCM research. In recent years, a number of non-targeted smart mass-spectrometry (MS) techniques, such as precise-and-thorough background-subtraction (PATBS) and metabolomics, have realized the intelligent identification of in vivo components of TCM. However, the metabolites characterization still largely relies on manual identification in combination with online databases.

**Results:**

We developed a scoring approach based on the structural similarity and minimal mass defect variations between metabolites and prototypes. The current method integrates three dimensions of mass spectral data including *m/z*, mass defect of MS1 and MS2, and the similarity of MS2 fragments, which was sequentially analyzed by a R-based mass dataset relevance bridging (MDRB) data post-processing technique. The MDRB technology constructed a component relationship network for TCM, significantly improving metabolite identification efficiency and facilitating the mapping of translational metabolic pathways. By combining MDRB with PATBS through this non-targeted identification technology, we developed a comprehensive strategy for identification, characterization and bridging analysis of TCM metabolite in vivo. As a proof of concept, we adopted the proposed strategy to investigate the process of exposure, metabolism, and disposition of Semen *Armeniacae Amarum* (CKXR) in mice.

**Significance:**

The currently proposed analytical approach is universally applicable and demonstrates its effectiveness in analyzing complex components of TCMs in vitro and in vivo. Furthermore, it enables the correlation of in vitro and in vivo data, providing insights into the metabolic transformations among components sharing the same parent nucleus structure. Finally, the developed MDRB platform is publicly available for (https://github.com/933ZhangDD/MDRB) for accelerating TCM research for the scientific community.

**Graphical Abstract:**

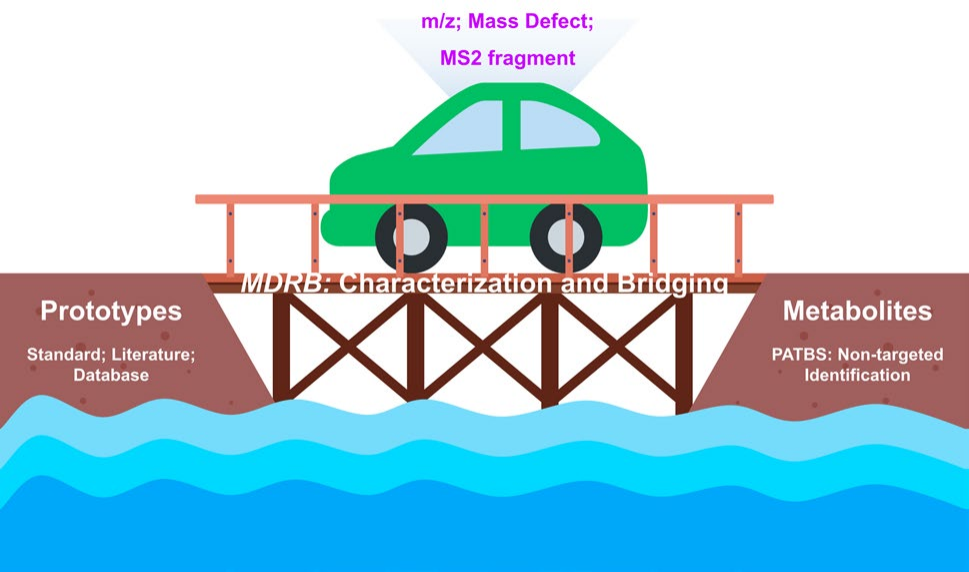

**Supplementary Information:**

The online version contains supplementary material available at 10.1186/s13020-024-01031-8.

## Introduction

Traditional Chinese medicine (TCM) has been known for having complex components, multiple biological targets, and usually relying on holistic effects, imposing challenges for active pharmacological ingredients identification and the corresponding in vivo metabolites characterization [1, 2]. In the past two decades, a number of research groups have made remarkable achievements in the identification of in vivo substances in TCM, and have developed a variety of high-resolution mass spectrometry (HRMS) data post-processing mining techniques, including targeted and non-targeted processing techniques [3]. Targeted techniques were based on known metabolic pathways or specific parent nuclei and functional group fragments. Typical examples can include high resolution-extracted ion chromatography (HR-EIC) [4, 5], mass defect filter (MDF) [6–8], product ion filter (PIF) [9–11], neutral loss filter (NLF) [9, 10, 12], and mass spectral trees similarity filter (MTSF) [13, 14]. Non-targeted techniques, such as precise-and-thorough background-subtraction (PATBS) [15, 16], or metabolomics techniques [17, 18], do not require prediction of the structure of herbal components. With these techniques, researchers realized the identification of TCM components in a more comprehensive manner in vivo. Furthermore, these techniques also offer partial characterization capabilities. For example, MTSF technology could intelligently perform tree mapping management of the acquired MS data, matching statistical scores of potential metabolites with similarity to the profile of the prototypes of the dendrogram, thereby correlating metabolites with their parent compounds without being limited by a predictable metabolic pathway. However, this technique only enables matching between single metabolite and the prototype database, and only utilizes the similarity of the secondary mass spectrometry maps. It should be noted that a single parent compound can produce multiple metabolites. Moreover, structurally similar parental compound may simultaneously produce metabolites with identical structures, which further complicated the matching process and impeding the interpretation of the metabolic process of TCMs in vivo. Therefore, the establishment of an effective correlation between in vivo and in vitro components would therefore significantly enhance current characterization efforts.

Generally, when components are detected to be transformed in vivo, the core structure of the parent compounds tend not to be significantly altered. The MS information of prototypes and metabolites tend to have a reasonable degree of similarity, especially for MS2 information, which is important to match the prototype and the further metabolites [18]. Furthermore, it has been shown that the mass defect between metabolites and the prototypes is usually in a certain range. Hence, the mass defect of the primary mass spectra can also help to correlate the prototypes and metabolites. However, compared to chemical drugs, metabolism of TCM is very complex and diverse, while the molecular changes of many metabolites are more significant and unpredictable. For example, some components of TCM with glycosidic structures may undergo other phase I and phase II metabolic reactions on the basis of deglycosylation, which might result in large changes in the moiety. In this case, the mass defect of MS2 is of great significance. Similar to the mass defect of MS1, the mass defect of MS2 fragments of prototypes and its metabolites is within a certain range, which can likewise provide critical information for bridging the prototypes and the metabolites. Therefore, this study will combine the similarity of MS2 fragment with MS1 and MS2 mass defect to develop a research method for the related substances in TCM, and to find the transformation relationship between the prototypes and metabolites in a more comprehensive, holistic, and accurate way.

As a traditional medicinal herb, Semen *Armeniacae Amarum*, also known as Chao Kuxingren (CKXR), has been widely used in TCM clinical formulas. Its clinical use is mainly for cough and asthma, chest fullness and phlegm, intestinal dryness and constipation [19]. It is an essential component of clinical treatments for COVID-19 (Jinhua Qinggan Granules, Lianhua Qingwen Capsule, Qingfei Paidu Soup, Huashi Baidu formula and XuanFei Baidu formula), due to its effective anti-inflammatory or antitussive active effect [20–24]. Current research on CKXR has focused on the quality control marker, Amygdalin, as recorded in the Chinese Pharmacopoeia, and its pharmacological activity and toxicity have also been well studied [25, 26]. However, studies on the in vivo metabolism and pharmacodynamic substance basis of CKXR in an integrated form are limited. Investigating the exposed substance profile of CKXR, comprehensively exploring the metabolic transformation pathways of CKXR, and constructing the whole process profile of ADME of CKXR from an in vivo perspective will provide valuable insights in its pharmacodynamic substances or underlying mechanism of action.

In this study, we integrated the mass spectrometry data of *m/z*, MS2 fragment matching and mass defect of the difference limit, to establish a novel mass dataset relevance bridging (MDRB) data post-processing technique, enabling the identification of metabolites and exploring the correlation between in vitro and in vivo data. Based on this, we proposed a complete strategy for the construction of in vivo and in vitro component network of TCM with adopting CKXR as a model herb. First, we realized the non-target identification of metabolites by our self-developed PATBS technique. Second, MDRB technique was employed to realize the characterization of metabolites and the bridge the correlation between prototype and metabolites, where in the current study, CKXR was used as a model herb, and the relevant components of CKXR in mice were maximally identified using PATBS. Furthermore, the identification of the exposed components of CKXR was completed in a stepwise manner through MDRB, achieving the analysis of the metabolic transformation pathway of CKXR in mice. Finally, the ADME profile of CKXR in vivo was constructed, providing a foundation for the exploration of the pharmacodynamic substances of CKXR.

## Method and material

### Chemicals and materials

The following chemicals and materials were used: Fried bitter almonds (Guosongtang Pharmaceutical Co., Ltd., Hebei, China); amygdalin, prunasin (purity ≥ 98%; Baoji Chenguang Biological Co., Ltd., Baoji, China); Oasis^®^ hydrophilic lipophilic balance (HLB) solid-phase extraction column (3 mL/60 mg; Waters, Milford, MA, USA); methanol and acetonitrile (HPLC grade; Tedia High-Purity Solvents, Fairfield, OH, USA); formic acid (LCMS / HPLC grade; Anaqua^™^ Chemicals Supply, Wilmington, DE, USA); normal saline (ChenXin Biology Co., Ltd., Jining, China); heparin sodium (Shanghai Aladdin Bio-Chem Technology Co., Ltd., Shanghai, China); ultrapure water preparation (MilliQ water purification system, Merck KGaA, Darmstadt, Germany); LPS (Beijing Solarbio Technology Co., LTD., Beijing, China); mouse interleukin (IL)-6 uncoated ELISA kits, mouse tumour necrosis factor alpha (TNFα) uncoated ELISA kits, and mouse IL-1β uncoated ELISA kits (Thermo Fisher Scientific Co., Waltham, MA, United States).

### Preparation of CKXR lyophilized powder

Weigh 100 g of CKXR and crush it, then add water to extract twice, the first addition of water is 5 times the amount of CKXR, *i.e.*, 500 mL, and decoct for 1 h. Filter to get the decoction of the first time; The dregs of the medicine were decocted for the second time, and the amount of water added was the same as that of the first time, and the decoction was boiled for 1 h. The decoction was filtered again to obtain the decoction solution; the two decoctions were combined, and the solution was left to stand, and then filtered to obtain the medicinal liquid; the decoction was continued to be concentrated. The obtained CKXR decoction concentrate was lyophilized to obtain the corresponding lyophilized powder of the medicinal herb.

### Study on metabolism of CKXR in mice

#### Animal experiment

Male C57BL/6 mice (18–20 g) were purchased from Xiamen University (Xiamen, China) Laboratory Animal Center. All animal care and experimental procedures were approved by the Animal Ethics Committee of Xiamen University (Animal Ethics Approval Number: XMULAC20200112, Xiamen University Animal Ethics Committee). All animals were acclimatized for 1 week prior to animal experiments and placed under standard conditions (12 h light/12 h dark cycle; temperature 25 ± 2 ℃; humidity 50–75%) with access to food and water. No food or water was allowed for 12 h prior to drug administration.

There were 35 mice, of which 5 were used to take blank plasma, blank lung tissue, blank urine, and blank feces. Blank samples were collected and stored at − 80 ℃. The remaining 30 were randomly divided into 6 groups of 5 mice each, and the mice were gavaged with CKXR administration solution. The dose was 10 g/kg, and the volume of gavage was 10 mL/kg. Plasma, lung tissue, urine and feces samples were collected after administration.

Blood was collected from mice by eye removal, and blood was collected from each group at 1, 2, 4, 6, 12, and 24 h after drug administration, collected in EP tubes containing sodium heparin (10 μL of 1% sodium heparin was added for every 0.5 mL of plasma), and centrifuged at a low speed of 4500 rpm for 10 min to obtain the supernatant, which was stored at − 80 ℃. The mice at each time point were killed by decapitation after blood sampling, and their lung tissues were collected, and the surface blood was washed with physiological saline and stored at − 80 ℃. At the same time, urine and feces were collected from 1–12 h to 12–24 h in 50 mL centrifuge tubes, placed in a sealed bag and stored at − 80 ℃.

#### Sample pretreatment

**Plasma sample pretreatment** Plasma samples from different time points were mixed according to the AUC pooled method (Table S1) to obtain a final volume of 960 μL [27, 28], The samples were diluted in an equal volume with ultrapure water, enriched and purified using an Oasis^®^HLB 3 mL column extractor. The methanol eluate was collected and dried using nitrogen as the drying medium. Blank plasma samples were processed with the same procedure as above.

**Urine sample pretreatment** Urine samples were collected from 0–12 to 12–24 h before and after drug administration, and 1.5 mL of each urine sample from different time periods after drug administration were mixed to obtain a final volume of 3 mL. At the same time, 3 mL of blank urine was taken. After centrifugation at 4500 rpm for 5 min to remove insoluble impurities, the samples were enriched and purified using an Oasis^®^HLB 3 mL column extractor, respectively. The methanol eluate was collected and dried using nitrogen as drying medium.

**Lung sample pretreatment** The lung tissues of mice collected at different time points were homogenized by adding 3 times the volume of saline. The homogenate was centrifuged at 4500 rpm for 5 min and the supernatant was taken to obtain the homogenized supernatant of lung tissue at each time point. 100 μL of homogenate supernatant was taken at different time points after drug administration and mixed to obtain a final volume of 600 μL of homogenate supernatant of lung tissue samples. The lung tissue samples were vortexed with 3 times the amount of methanol for 30 s, centrifuged at 13000 rpm for 10 min, and then the supernatant was collected and dried with nitrogen gas as drying medium. Blank lung tissue samples were processed as above.

The above-treated samples were re-solubilized with 100 μL of 70% methanol, vortexed for 30 s, sonicated for 10 min, and centrifuged at 13000 rpm for 10 min, respectively, and the supernatants were collected for analysis.

**Feces sample pretreatment** Feces were collected from pre-dosing and 0–24 h post-dosing, weighed and soaked in three times the amount (m/v) of saline for softening and then added with 3 times the amount of methanol for ultrasonic extraction, respectively. Centrifugation was carried out at 13,000 rpm for 10 min, and the supernatant was collected and left to be analyzed.

#### UPLC-HRMS analysis

The sample detection was performed with a Thermo Fisher Q Exactive Orbitrap mass spectrometer (Thermo Fisher Scientific Inc., Waltham, MA, USA). Mobile phases A and B were H_2_O with 0.1% formic acid and 100% acetonitrile, respectively. Sample separation was performed using an ACQUITY UPLC HSS T3 chromatographic column (2.1 × 50 mm, 1.8 μm; Waters, Milford, MA, USA) at 35 ℃, and the flow rate was 0.3 mL/ min. The gradients were set as follows: 0–2 min (2–2% B); 2–8 min (2–10% B); 8–16 min (10–12% B); 16–18 min (12–90% B); 18–20 min (90–98% B); 20–23 min (98–98% B); 23–23.1 min (98–2% B); 23.1–28 min (2–2% B). The eluent was monitored at the ultraviolet (UV) wavelengths of 207, 254, and 270 nm. The injection was performed at the volume of 3 μL for CKXR and 5 μL for the samples.

The MS parameters were set as follows: full MS resolution of 35,000; scanning range *m/z* of 100–1200; dd-MS2 resolution of 17,500; (N) collision energy (CE)/stepped (N) CE of 25, 30 and 35%; spray voltage ( +) of 3.8 kV; spray voltage (–) of 3.5 kV; capillary temperature of 320 ℃; sheath gas (N2) flow rate of 35 arb; sweep gas (N_2_) flow rate of 5 arb; and auxiliary gas (N_2_) flow rate of 10 arb.

#### Data analysis

Xcalibur software (version 3.0.63.3; Thermo Scientific) was used to collect the experimental data. The experimental data were processed using the MDRB data post-processing technology strategy proposed in this study. The MS2 dataset was obtained by data dependent scanning, compared with Pubchem (https://pubchem.ncbi.nlm.nih.gov/) and Chemspider (https://www.chemspider.com/) databases, and combined with the MS cleavage pattern, the structural characterization of the prototypes of CKXR was performed, and a list of the components of CKXR was obtained. The MS2 dataset of biological samples before and after drug administration was processed using PATBS to identify exogenous components in pairs of mice, which were defined as related components (including prototypes and metabolites) of CKXR in mice, and a list of related components of CKXR in mice was obtained. Finally, the in vivo and in vitro component lists are progressively correlated using the MDRB algorithmic strategy, and the relevant algorithms are uploaded to the platform (https://github.com/933ZhangDD/MDRB) and are publicly accessible.

### Study on the efficacy of CKXR in vitro

#### Cell culture

Mouse macrophage RAW264.7 cells were cultured in Dulbecco’s modified Eagle medium (Gibco, Carlsbad, CA, USA) containing 20% fetal bovine serum and 1% penicillin–streptomycin in an incubator containing 5% CO_2_ at 37 ℃.

#### Cell sample pretreatment

RAW264.7 cells were cultured with medium containing LPS (1 μg/mL) for 24 h for inflammation model induction, and then added to medium containing amygdalin (6.25 μmol/L) and prunasin (6.25 μmol/L) and CKXR (10 mg/mL) for the treatment of drug administration, respectively. After co-incubation for 24 h, the supernatant was collected and centrifuged at 2000 rpm for 5 min and set aside for ELISA analysis; the cells were washed with PBS, and RNA was extracted for qRT-PCR analysis.

#### Quantitative real-time PCR (qRT-PCR)

Trizol reagent was used to extract the total RNA, and the isolated RNA was dissolved in 20 μL of diethyl pyrocarbonate and stored at − 80 ℃ until further use. The reaction system was 20 μL in volume, containing 1 μL of sample template DNA, 10 μL of one-step Hieff^®^ qPCR SYBR^®^ Green Master Mix (Low Rox Plus), 10 mM primer (0.4 μL each), and 8.2 mL of ultrapure water. The reaction conditions were 95 ℃ for 5 min (1 cycle), 95 ℃ for 10 s, and 60 ℃ for 30 s (40 cycles in total). Agilent AriaMx (Agilent Technologies Co., Ltd., Palo Alto, CA, USA) was utilized for the analysis of PCR products. The primers (Xiamen Bioray Biotechnology Co., Ltd., Fujian, China) used for qRT-PCR were as follows: glyceraldehyde-3-phosphate dehydrogenase (GAPDH) (F: ATG TGTCCGTCGTGGATCTGA; R: TGCCTGCTTCACCTTCT), IL-1β (F: ACCCCAAAAGATGAAGGGCTG; R: TACTGCCTGCCTGAAGCTCT), IL-6 (F: ATCCAGTTGCCTTCTTGGGACTGA; R: TTGGATGGTCTTGGTCCTTAGCCA;), TNF-α (F: CATCTTCTCAAAATTCGAGTGACAA; R: TGGGAGTAGACAAGGTACAACCC). The 2^−△△CT^ method was used to calculate the IL-6, IL-1β and TNF-α expression levels, with GAPDH as the internal reference.

#### Enzyme-linked immunosorbnent assay (ELISA)

The concentrations of pro-inflammatory cytokines in the cell culture media of different dosing groups were detected using IL-1 beta Mouse ELISA Kit, IL-6 Mouse ELISA Kit and TNF alpha Mouse ELISA Kit.

## Result

### Libraries establishment and mass dataset relevance bridging (MDRB) strategy

**Fig. 1:**
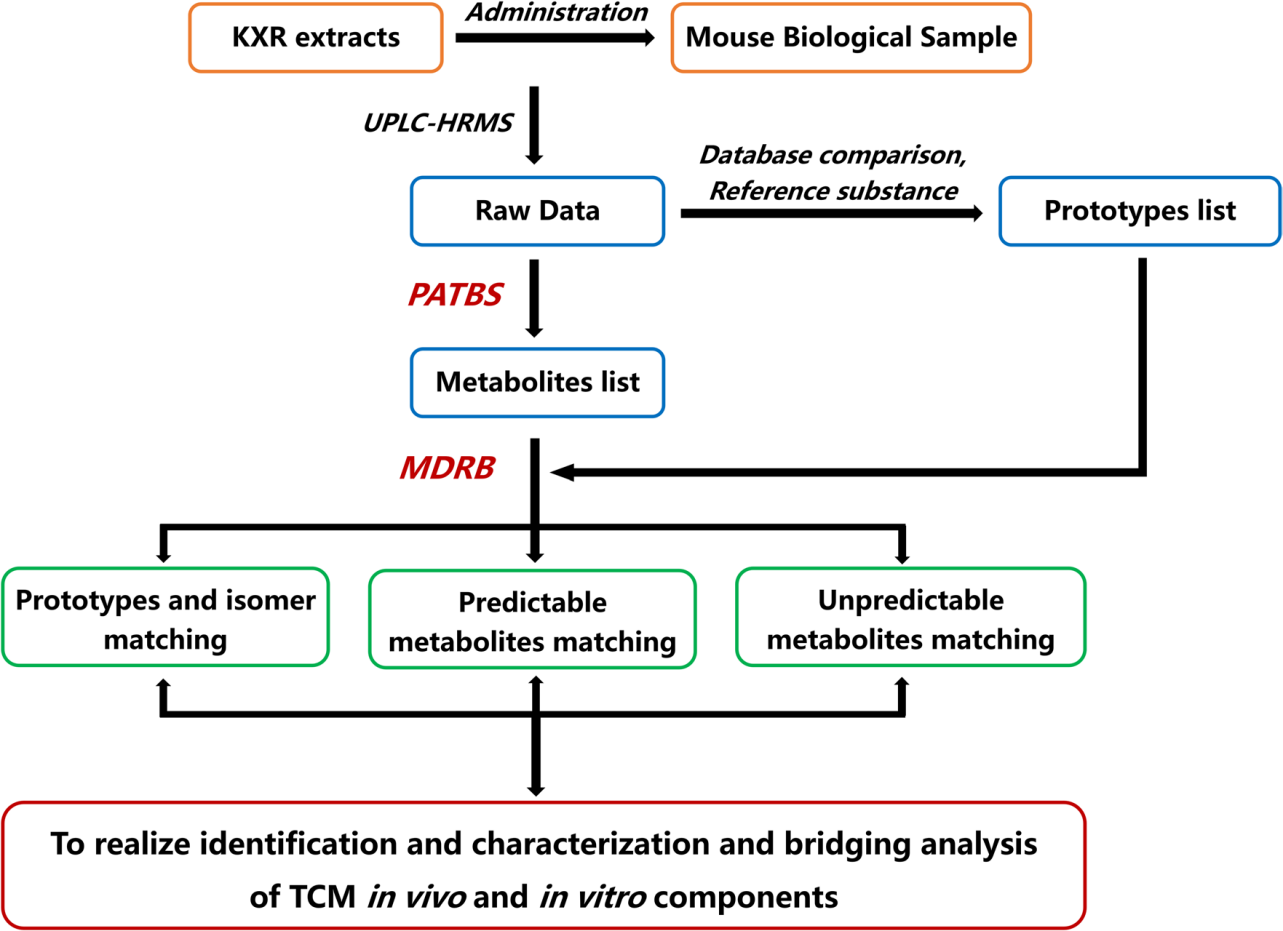
A workflow of using UPLC–HRMS, PATBS and the mass dataset relevance bridging (MDRB) technique for the discovery and identification of metabolites

In order to realize the transformation attribution of relevant components in TCM and to comprehensively construct important in vivo metabolic profiles, we established a novel strategy for the identification, characterization, and bridging analysis of in vivo metabolites (Fig. 1). The first step is to establish two lists, including prototype list and metabolite list. Identification of prototypical components was carried out by the fingerprint analysis of CKXR to generate a prototype list. The metabolite list mainly relies on the PATBS non-targeting technique. This technique is mainly based on retention time (RT) and *m/z*, as well as setting a narrow mass window (10 ppm) and time window (± 0.5 min), comparing the blank and sample spectra, and realizing the background subtraction of the biological samples according to a certain subtraction ratio. In this case, the mapping and maximize the obtainment of CKXR related components in mice can be simplified, in order to constitute a metabolite list. Secondly, we utilized the MDRB strategy to achieve progressive correlation of in vivo and in vitro components, which is mainly divided into three steps: prototypes matching, predictable metabolites matching, and unpredictable metabolites matching. This matching is mainly based on MS1, MS2, RT, *m/z*, and mass defect information, which is assigned by different comparison and screening methods to construct the association between prototypes and metabolites. Employing this holistic analysis strategy, we can identify and characterize the metabolites and map the metabolic networks of TCM in vivo, offering a visual representation of its overall mechanism of action.

### Recognition of prototypic components and metabolites in vivo

**Fig. 2:**
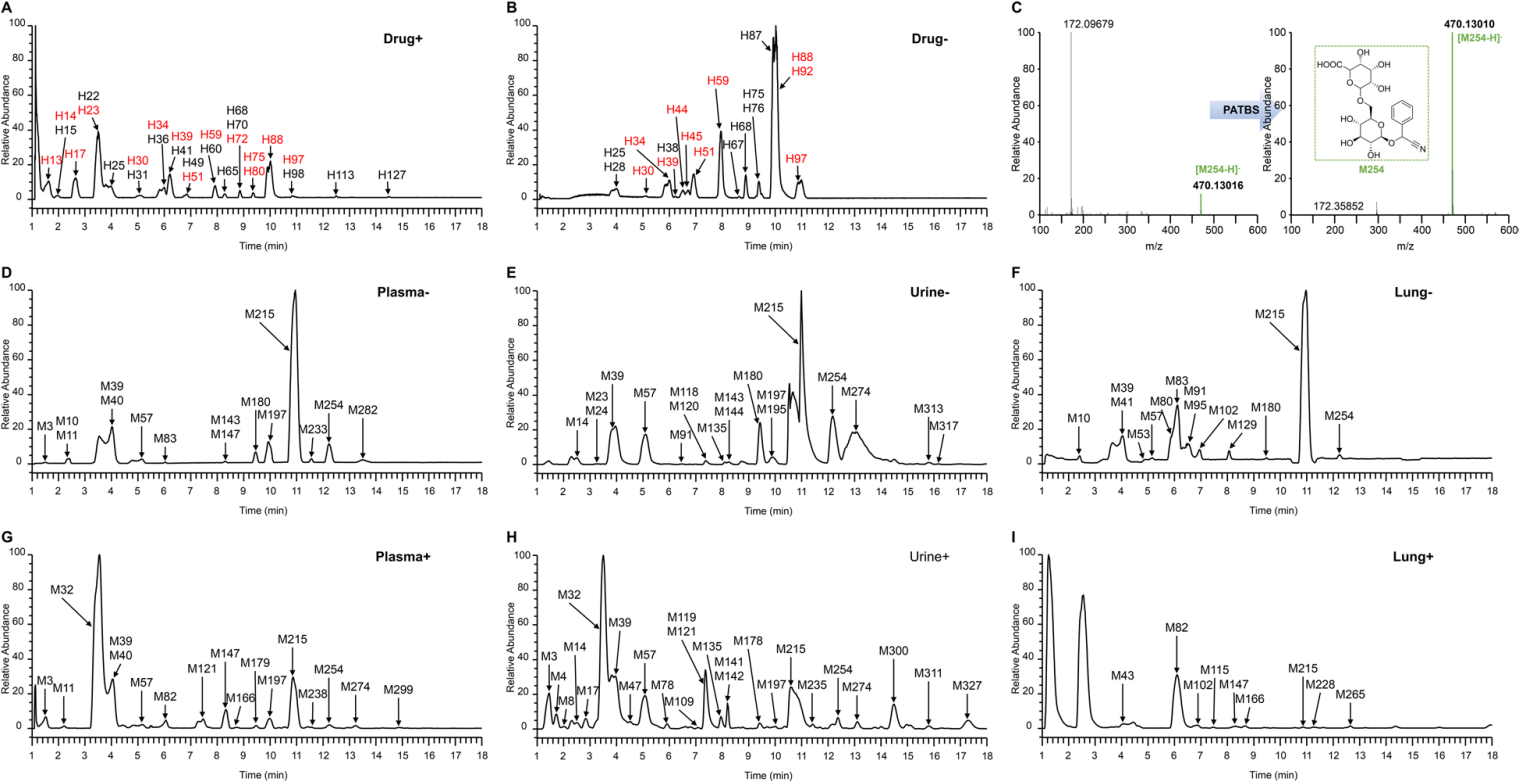
High-resolution total ion chromatogram of CKXR in positive ion mode (**A**) and in negative ion mode (**B**). **C** Full-scan MS spectrum of M254 from PATBS-processed UPLC-HRMS dataset in the negative mode. **D**–**I** After PATBS-processed chromatography of full-scan MS analysis of plasma, urine and lung after administration in positive and negative ion mode

The CKXR sample was detected to obtain the high-resolution total ion chromatogram (TIC) in positive and negative ion modes. Different components exhibited different retention behaviors on the column and produce characteristic MS2 fragmentation patterns. The identification and characterization of the components of CKXR were carried out based on various methods, such as the identification of the reference substances, and the comparison of the literature and databases [Pubchem (https://pubchem.ncbi.nlm.nih.gov/) and Chemspider databases (https://www.chemspider.com/Default.aspx)]. We found a total of 199 components in CKXR (Fig. 2A, B). Among them, 96 substances were detected in the positive ion mode, while 136 substances were detected in the negative ion mode, and 33 substances responded to both positive and negative ion modes. Detailed MS information of the relevant components of CKXR is shown in Table S2. Our experiment conjectured the possible structures of 128 components, of which 15 (including amygdalin, prunasin, and chlorogenic acid) were characterized by the reference substances or literature. During the process of identification and characterization, we found that CKXR had the highest relative contents of amygdalin and prunasin. Their RT and MS2 information were consistent with the reference substances, and the specific MS2 information was resolved in Figure S1. Finally, we constructed a list of prototypes of CKXR.

Furthermore, we used the PATBS technique for non-targeted selection of peaks of CKXR-related components in biological samples, which can simplify MS information and accurately and completely remove the signals of endogenous interfering substances in full MS datasets [14, 15]. In Fig. 2C, before PATBS treatment, M254 showed low abundance because of the presence of high abundance endogenous components. This might be easily overlooked during MS1 data retrieval. However, MS1 information of M254 was presented in a more effective manner after PATBS technique treatment. PATBS enables comprehensive, non-targeted identification of differential components in samples before and after drug administration. The interference of endogenous components can be effectively excluded, thus rapidly obtaining CKXR-related components in the administered biological samples. We obtained 351 CKXR-related exogenous components, including 41 prototypes and 310 metabolites in mice. 87 CKXR-related components were retrieved from plasma; 53 CKXR-related components were retrieved from lung tissues; 342 CKXR-related components were retrieved from urine; and 42 CKXR-related components were retrieved from feces. All identified CKXR-related components were compiled into a metabolite list. Detailed MS information of CKXR-related metabolites in mice is shown in Table S3 and Fig. 2D–I.

### Characterization and bridging between in vitro and in vivo components based on MDRB

**Fig. 3:**
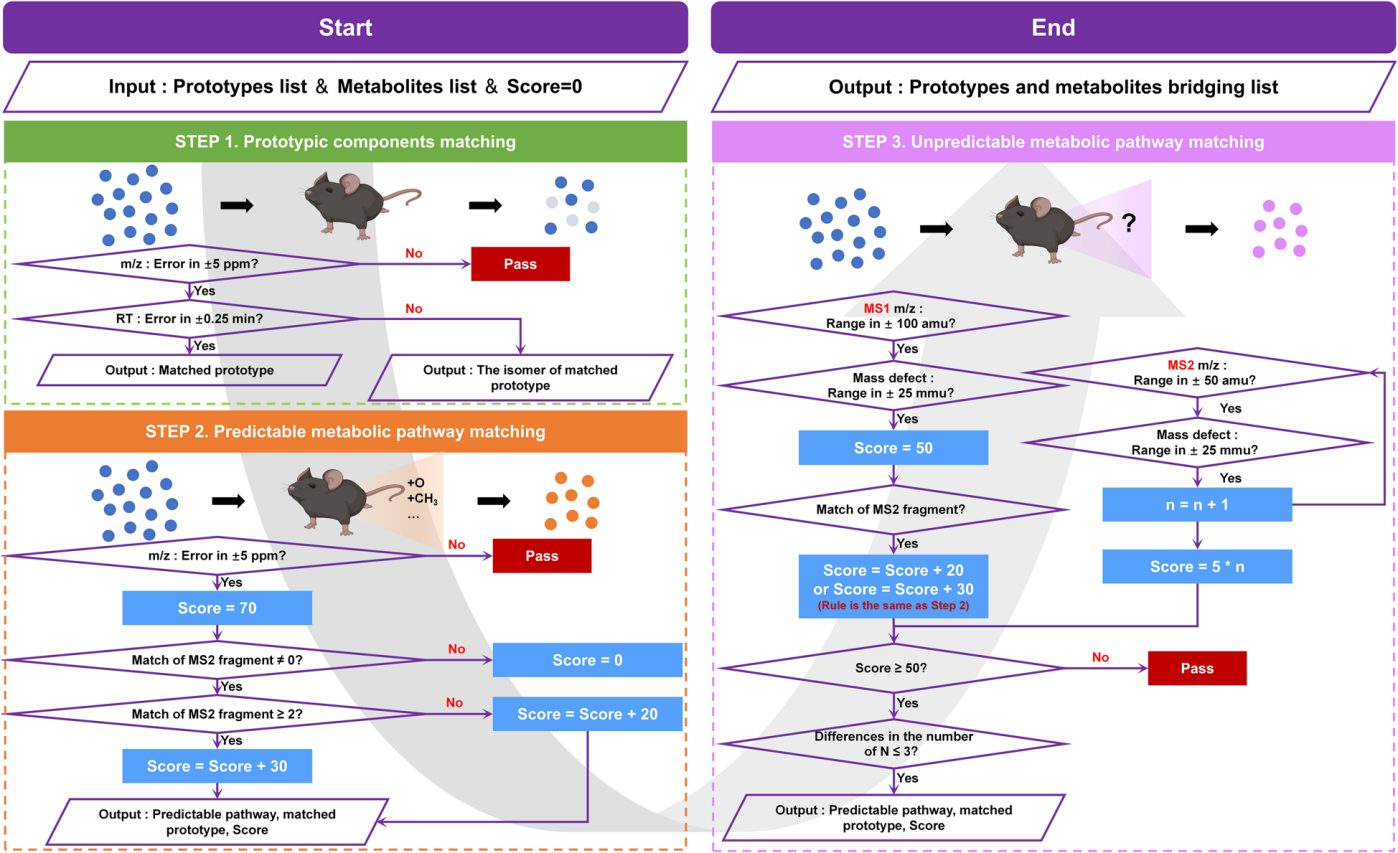
Algorithm assignment process and scoring mechanism

Characterization of metabolites usually relies on predictable metabolic pathways, achieved through primary mass number changes and MS2 similarity. However, this process does not cover unpredictable metabolites and often results in the loss of information related to potential active ingredients [29]. Since the mass defect between metabolites and prototypes tends to be within a certain range, mass defect can play a key role in the search for characterization of unpredictable metabolites [30]. In order to efficiently and comprehensively obtain the network of relationships between prototypes and metabolites, we established the MDRB strategy, which synthesizes the predictable changes in the *m/z*, the mass defect of MS1 and MS2, and the similarity of MS2 fragments for the characterization of metabolites and their correlation bridging with the prototypes. In summary, the characterization and association of the list of prototypes (Table S2) and the list of related components in mice (Table S3) were achieved through a step-by-step matching process. During the matching process, we established an assignment mechanism to facilitate researchers to visualize the intensity of the association between the two (Fig. 3), and a screenshot of the relevant key codes can be found in Figure S2.

The first step is the characterization of prototypes and isomers of TCM. We set screening standards of RT of ± 25 s and *m/z* of ± 5 ppm to characterize the prototypes of TCM in mice. The screening standard for isomerism was then *m/z* within ± 5 ppm error, but RT was not within ± 25 s.

The next step is the characterization of predictable metabolites, which is the products of established and anticipated metabolic pathways. We based the known predictable metabolic responses provided by Metworks 1.3.0.200. Combined with the characteristics of TCM components, we added the deglycosylation reactions that TCM components are easy to undergo within living organisms (Table S4). With the help of databases to predictable metabolites, a catalog of high-resolution *m/z* values of expected metabolites that undergo one- or two-step metabolic reactions is established. When the *m/z* of a component in the metabolite list in mice was consistent with the *m/z* of a prototypes after a predictable metabolic reaction (within ± 5 ppm), it was initially included in the category of possible metabolites of the prototypes and assigned a score of 70, which was further validated. MS2 fragments can provide more information for the association between metabolites and prototypes, and we assign a score to the similarity match hierarchy of MS2 information. When the degree of match is 1, 20 points will be assigned. When the degree of match is greater than or equal to 2, 30 points will be assigned. When the degree of match is 0, 0 points will be assigned. And it is initially concluded that there is no metabolic transformation relationship between the two under consideration.

The final step is the characterization of unpredictable metabolites, which is the products undergo unpredictable metabolic pathways. Here we introduced mass defect to filter of large-scale datasets. Different mass defect ranges (± 10 mmu, ± 25 mmu, ± 50 mmu, and ± 75 mmu) were selected for optimization in order to achieve a more comprehensive and efficient pathway attribution for the prediction of metabolite ground transformation in TCM (Figure S3). The results show that a narrower range, with a reduced proportion of metabolic pathways that can be included (Figure S3A), is able to largely exclude the interference of endogenous components, but the positivity rate is affected to some extent (Figure S3B). The wider range allows for higher positivity rates, but there is often interference that cannot be eliminated when performing multi-component metabolic transformation correlations, leading to increased false positivity rates (Figure S3C). Therefore, we chose ± 25 mmu as MS1 and MS2 mass defect range for unpredictable metabolic pathway matching between prototypes and metabolites. This mass defect range can exploit more effective metabolite information, provide more accurate metabolic transformation relationship, greatly reduce false positive results, and have better practical application value. **MS1 Mass Defect Screening Standard: **When MS1 *m/z* of a component in the metabolite list in mice falls within ± 100 amu of MS1 *m/z* of a prototypes and has a mass defect of ± 25 mmu or less, it is initially included in the range of unpredictable metabolites of prototypes and assigned a score of 50. Matching of MS2 fragmentation information was then performed, with matches and scores assigned consistent with predictable metabolic pathways. **MS2 Mass Defect Screening Standard: **MS2 fragments of a component in the metabolite list are matched one-to-one with MS2 fragments of the prototypes. When MS2 *m/z* of the metabolite falls within ± 50 amu of MS2 *m/z* of the prototypes and the mass defect is within ± 25 mmu, the matching degree will be increased by one accordingly, and 5 points will be assigned for every instance of successful match. Finally, after manually checking for matches of unpredictable metabolites, we set a retention score of 50. In addition, we scrutinized the known metabolic pathways, adding to define that the change in the number of N between prototypes and metabolites was not greater than 3.

Components that do not fall within the scope of the previously mentioned steps are considered elusive in terms of their metabolic pathways. To date, these metabolites have evaded identification of their biochemical processes. It is worth noting that the additive form of components is not limited to the addition or subtraction of hydrogen ions, and we have fully considered the effects of different additive forms (e.g., [M + Na]^+^, [M + NH4]^+^, [M + HCOO]^−^) during the actual matching process, which can be adjusted according to the actual results. Meanwhile, predictable metabolic pathways can equally be adapted to one's own samples. In addition, to increase the confidence of MS2 fragmentation information, we also defined the mass defect between MS1 and MS2, assigning a score of 2 when it falls within ± 50 mmu.

In summary, the main factors of scoring are *m/z* of MS1, *m/z* of MS2 and mass defect. Here, we believe that scoring is extremely related to the importance of factors. Factors more valuable to the matching process were assigned higher scores. Specifically, MS1 plays an important role in predictable metabolic pathway. Only when MS1 of the signal to be matched is within the range of conditions can it be considered as a predictable metabolite. At the same time, mass defect is of vital importance for the unpredictable metabolic pathway. Substances with mass defect beyond the allowed range will be regarded as uncommon metabolites and thus be removed from the list of metabolite candidates. MS2 mainly supports the matching process. If MS2 of a signal is similar to that of prototype, the matching would be more reliable. Take metabolite A, and its matching signal (signal B and signal C) as an example. After step 2 process (predictable metabolic pathway matching), signal B and C both could be regarded as predictable prototypes of metabolite A. However, the difference is that signal B could match three MS2 with prototype A, while signal C could only match one MS2 with metabolite A. In this case, prototype B is assigned with 100 scores and prototype C is only assigned with 90 scores. Thus, signal B is more likely to be the predictable prototype of metabolite A.

To more intuitively illustrate the data processing effects of MDRB, we compared the number of metabolites characterized with and without MDRB. A total of 110 metabolites could be characterized manually through matching predictable metabolic pathways, calculation of *m/z* differences, and comparison of MS2. The rate of manual characterization was 31%. However, utilizing MDRB for data processing analysis, 227 metabolites could be characterized through prototype matching, predictable metabolic pathway and unpredictable metabolic pathway matching, with the characterization rate of 65% (Figure S4A). It can be seen that the use of MDRB can greatly improve amounts and characterization rate of metabolite compounds. Furthermore, we took amygdalin and prunasin as examples, comparing metabolic profiles constructed separately based on manual characterization (Figure S4B) and based on MDRB (Figure S4C). We found that the metabolic profiles were more comprehensive and comprehensive after MDRB treatment. In summary, MDRB tool analysis can increase the number of metabolites characterized and provide more information for constructing metabolic profiles.

The correlation of 227 CKXR-related components in mice was accomplished using the MDRB technique described above. We characterized 66 relevant metabolites in plasma, 222 relevant metabolites in urine, 30 relevant metabolites in feces, and 40 relevant metabolites in lung tissue. Specific matching information is presented in the Table S5. The MDRB technique proposed in this study enables a more comprehensive metabolite characterization and a complete construction of the relationship network of TCM components in vivo and in vitro. The MDRB has a 65% identification efficiency. Of note, by defining the MS2 mass defect, we can achieve more metabolite-prototypes association construction, which is beneficial to adequately characterize the relevant metabolites in TCMs in vivo.

### Metabolic transformation relationship of cyanogenic glycosides

**Fig. 4:**
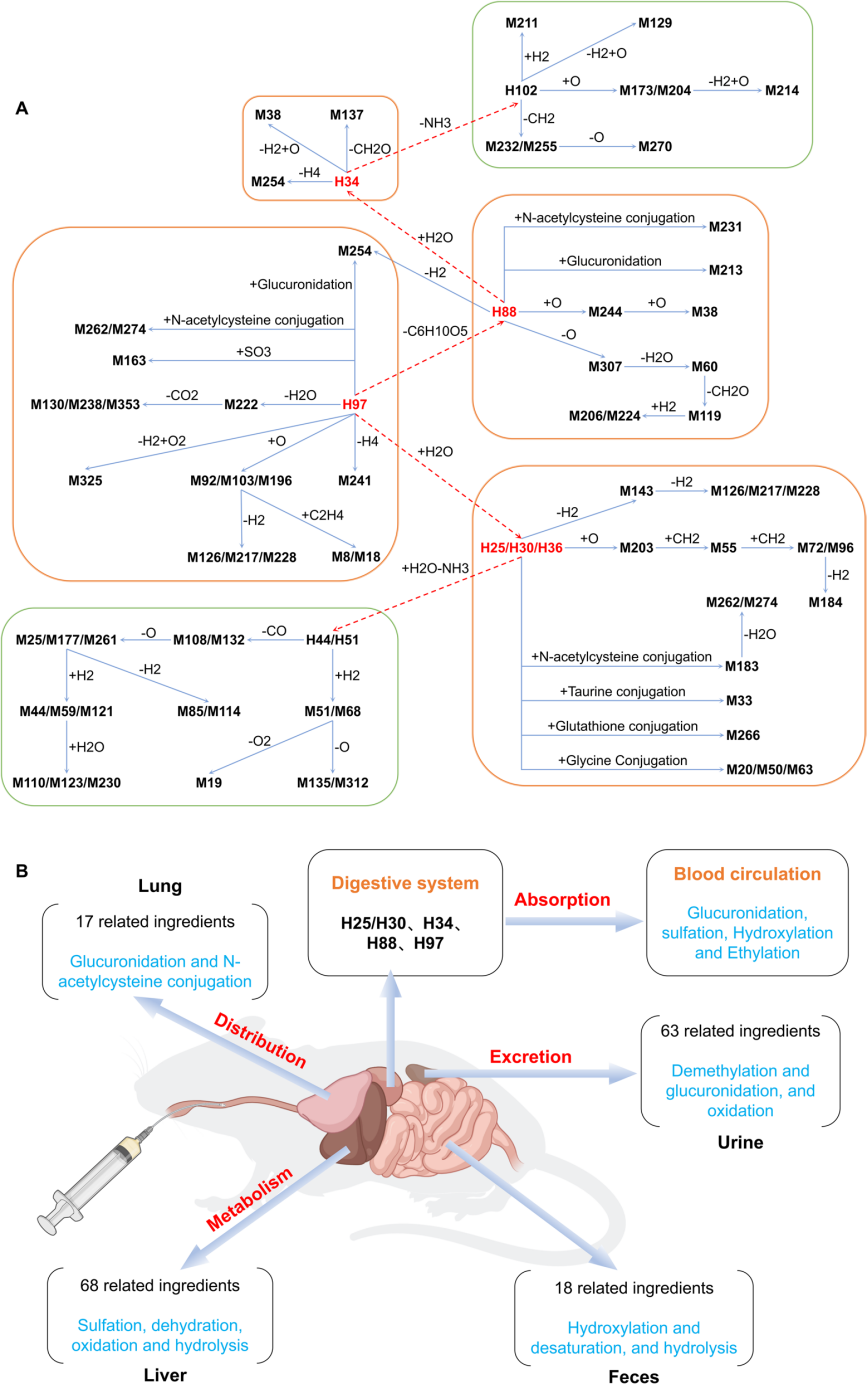
Exposed mass spectrum (**A**) and ADME metabolism profile (**B**) of cyanogenic glycosides from CKXR in mice

Following the outlined analytical approach, we procured the scoring and correspondence data (Table S4) pertaining to the constituents linked with CKXR within murine models. Consequently, we were able to delineate a predictive trajectory for metabolic conversions, specifically attributing these processes to the related metabolites of CKXR in mice. By leveraging AUC pooled method, we streamline intricate pharmacokinetic models through consistent criteria, significantly diminishing the burden of sample collection and the investment of time in analysis. This enables us to swiftly and effortlessly uncover the pronounced exposure levels of herbal components within biological systems. Such an efficient approach to pharmacokinetic assessment accelerates research progress while optimizing the monitoring of in vivo behavior of active compounds, making it both cost-effective and resource-efficient [27, 28]. We are concerned that cyanogenic glycosides represented by amygdalin (H88/M197) and prunasin (H97/M215) are the main exposed components of CKXR in mice. Therefore, we analyzed the metabolic characteristics of cyanogenic glycosides, centering on amygdalin and prunasin, and constructed the exposure profile of cyanogenic glycosides in mice (Fig. 4A). Meanwhile, we mapped the ADME metabolic profile of the cyanogenic glycosides of CKXR in mice based on the detection of cyanogenic glycosides in plasma, lung, urine and feces (Fig. 4B).

### Efficacy verification of CKXR-related components in LPS-induced cell inflammation model

According to our study, prunasin (H97) has an extremely high exposure in vivo. Furthermore, it has been shown that part of prunasin is metabolized from amygdalin in vivo and that amygdalin (H88) also has a relatively high exposure, which was also verified in our laboratory. Amygdalin and prunasin are the same cyanogenic glycoside analogs, and in the foregoing, we have successfully constructed a spectrum of cyanogenic glycoside-exposed components from CKXR in mice. We used AUC pooled method to analyze the related components in plasma [27, 28]. With this method, the peak area can represent in vivo exposure of the components to some extent. We added all related peak areas of cyanogenic glycosides in CKXR under positive ion mode, and found that they accounted for 43.16% of the whole peak areas. Similarly, we added all related peak areas of cyanogenic glycosides in CKXR under negative ion mode, and found that they accounted for 53.57% of the whole peak areas. Relevant studies have reported that amygdalin has definite anti-inflammatory effects [25]. Prunasin has a higher exposure and it was interesting to see whether it possessed an anti-inflammatory effect comparable to amygdalin. We selected two cyanogenic glycosides, amygdalin and prunasin, which have been successfully structurally identified, and also set up the group of CKXR as a whole to initially investigate their anti-inflammatory activities. Based on the concentration of amygdalin in CKXR, we chose the concentration of 10 mg/mL of CKXR medicinal solution and 6.25 μmol/L of amygdalin, in which the concentration of amygdalin was comparable. The concentration setting of prunasin was consistent with that of amygdalin.

**Fig. 5:**
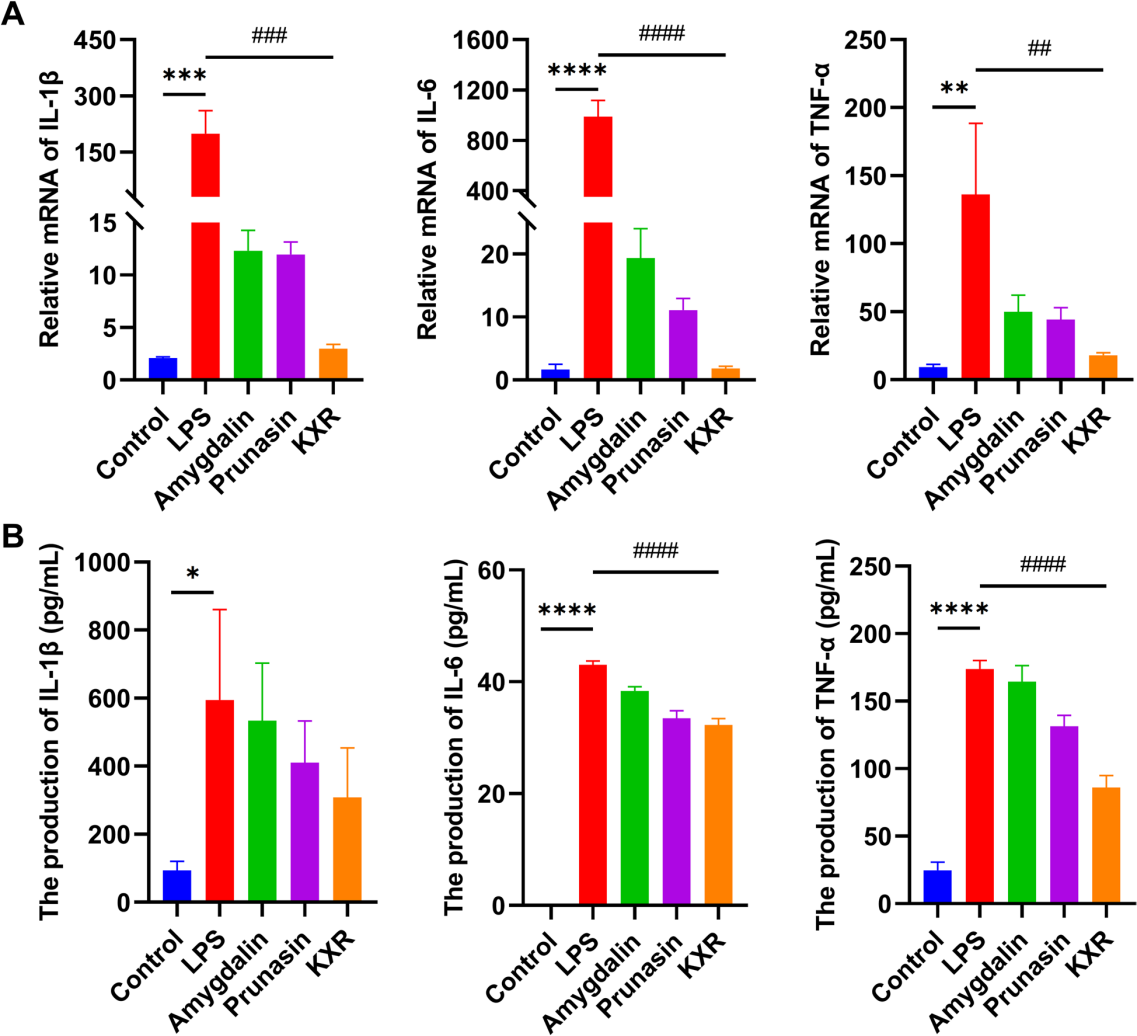
Effects of amygdalin, prunasin and CKXR on the expression of IL-1β, IL-6 and TNF-α in LPS-induced RAW264.7 cell inflammation model. **A** Effect of mRNA expression level; **B** Effect of release level. *p < 0.05, **p < 0.01, ***p < 0.001, ****p < 0.0001

**qRT-PCR.** Figure 5A showed that the model group could induce a significant increase in the mRNA expression of pro-inflammatory cytokines IL-1β, IL-6 and TNF-α in RAW264.7 cells after 24 h of LPS stimulation compared with the normal control group, and the differences were all statistically significant (p < 0.001, p < 0.0001, p < 0.01). The mRNA expression of IL-1β, IL-6 and TNF-α was significantly down-regulated in all groups after the intervention with amygdalin, prunasin and CKXR compared to the model group. The differences in IL-1β and IL-6 were statistically significant in the CKXR, amygdalin and prunasin groups compared to the model group (p < 0.001, p < 0.0001). However, down-regulation of TNF-α by CKXR (p < 0.01) was more significant than that of amygdalin and prunasin groups (p < 0.05).

**ELISA.** Figure 5B shows that the model group induced a significant increase in the expression of pro-inflammatory cytokines IL-1β, IL-6 and TNF-α in RAW264.7 cells after 24 h of LPS stimulation compared with the normal control group, and the differences were all statistically significant (p < 0.05, p < 0.0001, p < 0.0001). Compared with the model group, the expressions of IL-1β, IL-6 and TNF-α were all down-regulated after the administration of the intervention, although their down-regulation of IL-1β was not significant. However, the down-regulation of both IL-6 and TNF-α by CKXR was statistically significant (p < 0.0001, p < 0.0001).

Combining the above qRT-PCR and ELISA results, we found that both of amygdalin and prunasin have anti-inflammatory effect, in which the anti-inflammatory effect of prunasin is superior to that of amygdalin at the same concentration. This suggests that amygdalin and prunasin are most likely dependent on the consistent parent structure of both in exerting their anti-inflammatory effects. For prunasin, only one glycoside is linked to the parent structure, and its affinity for potential targets may be stronger than that of amygdalin. In addition, we found that when the CKXR administration solution was compared with the amygdalin group at comparable concentrations, the anti-inflammatory activity of the monomer was not superior to that of the overall. This suggests that the anti-inflammatory effect of CKXR is not solely exerted by amygdalin, but other cyanogenic glycosides (including prunasin) also play an important role and deserve further attention. The specific mechanism of superimposed or synergistic effects of different cyanogenic glycosides can be further verified in the future.

## Discussion

Traditional Chinese medicines (TCMs), with a history of over two thousand years, have accumulated a profound historical heritage, embodying the extensive wisdom of the Chinese people and the Chinese nation. They are regarded as the quintessence of China’s traditional medical system. There is a growing interest in developing TCM-related drugs for clinical therapeutic applications. A full realization of the substance basis and mechanism of effect of TCM has become a pressing issue, while the most difficult part of which is the complexity of TCM itself. Through the in vivo exposure perspective, the active substance component of TCMs can be screened more rapidly to some extent, thus facilitating the understanding of TCMs [15, 31]. The complexity of TCM components brings great challenges to the identification and characterization of substances related to their metabolic transformation after entering the body. In the last two decades, various non-targeted and targeted smart MS data post-processing techniques have better solved the recognition of components in vivo, but there are still more difficulties in the identification of components.

In this study, based on the predictable change of *m/z*, the mass defect between MS1 and MS2, and the similarity of MS2 fragments, we established a technique, MDRB, for in vivo metabolite characterization in TCM and its bridging with in vitro prototypes. Combining it with the PATBS technique, we developed a new analytical strategy for the metabolite identification and characterization of TCMs in vivo. This strategy realizes the construction and interpretation of the relationship network of TCM components in vivo and in vitro, and the processing flow is simple without other additional operations. Based on this new strategy, the study took CKXR as the research object, used PATBS technique to recognize metabolites, and comprehensively explored the correlation between the prototypes and metabolites of CKXR in mice by MDRB technique. We found that the main exposed substances in mice consisted of cyanogenic glycosides through AUC pooled method. We finally found 351 components and completed the correlation of 227 CKXR-related components in mice, obtained the metabolic network of CKXR in mice, and deeply analyzed the in vivo metabolic characteristics. We constructed the exposed component profiles and ADME metabolic profiles of cyanogenic glycoside components, which can help the study of the pharmacological effect and substance basis of CKXR [32]. In addition, we constructed an inflammation model by LPS-induced RAW264.7 cells. We observed the anti-inflammatory effect of amygdalin, prunasin and CKXR, and explored the possible pharmacodynamic substance basis of CKXR.

The strategy established in this study for identifying, characterizing, and bridging the metabolites of TCMs in vivo demonstrates strong generalizability and can be applied to the correlation structures of metabolites across a wide range of TCMs. Notably, the method is capable of comprehensively mining out components (including prototypes and metabolites) with the same parental structure, thus realizing the construction of exposed substance profiles of the same type of components. The analysis strategy proposed in this study offers a more efficient and comprehensive approach for the identification and characterization of metabolites of TCMs in vivo, outperforming the capabilities of a standalone intelligent data processing platform.

## Conclusion

This study introduces an innovative strategy for the in vivo identification, characterization, and bridging analysis of traditional Chinese medicine (TCM) metabolites. Employing the PATBS technology in conjunction with the newly developed MDRB, a technology that correlates in vivo and in vitro components of TCM, the research achieves a comprehensive characterization of metabolites and their correlations with parent compounds. This approach also facilitates the construction of exposed profiles and ADME metabolic profiles, suggesting its potential for holistic identification and characterization of TCM metabolites within living organisms. As a case study, the research delves into the prototypes and metabolites of CKXR in mice, utilizing the AUC pooling method to visually represent and characterize the highly exposed components. An in-depth analysis of the metabolic features of the primary exposed substances, including cyanogenic glycosides, is also conducted. The study thus provides a thorough understanding of the exposure and metabolism of CKXR in mice. Additionally, the research constructs exposed profiles of cyanogenic glycosides derived from CKXR in mice and maps of their metabolic profiles, laying the groundwork for exploring the pharmacological substance basis of CKXR. Overall, this study not only advances our knowledge of TCM metabolite analysis but also sets the stage for future investigations into the therapeutic mechanisms of TCMs.

## Supplementary Information

Additional file 1.

## Data Availability

All data for the duration of the study can be found in this article and appendix.
