# Supplementary material for: An integrated approach for studying exposure, metabolism, and disposition of traditional Chinese medicine using PATBS and MDRB tools: a case study of semen Armeniacae Amarum
Source: Chin Med. 2024 Nov 14;19:158. doi: 10.1186/s13020-024-01031-8 (PMC11566643; doi:10.1186/s13020-024-01031-8)
Supplement: Supplementary file 1 — Additional file 1. [file 13020_2024_1031_MOESM1_ESM.docx]

***Supplementary data***

**An Integrated Approach for Studying Exposure, Metabolism, and Disposition of Traditional Chinese Medicine using PATBS and MDRB Tools: A Case Study of Semen *Armeniacae Amarum***

Dandan Zhang^1 #^, Junyu Zhang^1 #^, Simian Chen^1 #^, Hairong Zhang^1^, Yuexin Yang^1^, Shan Jiang^1^, Yun Hong^1^, Mingshe Zhu^4^, Qiang Xie^3^ *, Caisheng Wu^1,2^ *

^1^ Fujian Provincial Key Laboratory of Innovative Drug Target Research and State Key Laboratory of Cell Stress Biology, School of Pharmaceutical Sciences, Xiamen University, Xiamen, Fujian, 361102, China

^2^ Xiamen Key Laboratory for Clinical Efficacy and Evidence-Based Research of Traditional Chinese Medicine, Xiamen University, Xiamen, 361005, China

^3^ Department of Cardiology, The First Affiliated Hospital of Xiamen University, School of Medicine, Xiamen University, Xiamen 361005, China

^4^ Mass Defect Technologies, Princeton, NJ, USA

^#^These authors contributed equally to this work and should be considered co-first authors.

*Corresponding author: Caisheng Wu (E-mail: [wucsh@xmu.edu.cn)](mailto:wucsh@xmu.edu.cn)) .

Amygdalin is composed of benzaldehyde, hydrocyanic acid and D-mannitol-*β*-d-glucoside-6-*β*-glucoside. The retention time of amygdalin (H88) was 10.01 min for [M+NH_4_]^+^ ion in positive ion mode, *m/z* 475.1921 (error: -0.392 ppm). The negative ion mode was [M+HCOO]^-^ ion, *m/z* 502.1565 (error: -0.305 ppm). Based on the MS/MS data of the parent ions, we extrapolated the possible cleavage modes of amygdalin in the positive and negative ion modes, respectively. In the positive ion mode, the para-molecular ion peak of amygdalin was *m/z* 475.1921 ([M+NH_4_]^+^), followed by the loss of a glycoside yielding the characteristic ion peak *m/z* 296.1119, which could be prunasin or its isomer. And the four fragments, *m/z* 97.0288, 127.0390, 145.0495 and 163.0600, are mainly fragment ions produced by the sequential loss of H_2_O that occurs from the glycosides lost on the amygdalin. In the negative ion mode, the para-molecular ion peak of amygdalin was *m/z* 502.1565 ([M+HCOO]^-^). Cleavage of the amygdalin subsequently occurred and the cleavage ion D-mannitol-*β*-d-glucoside-6-*β*-glucoside was detected at *m/z* 323.0984. The glycoside then undergoes a reaction of ring opening and sequential loss of H_2_O producing a series of associated fragment ions.

Prunasin are composed of benzaldehyde, hydrocyanic acid, and 6-*β*-glucoside. Prunasin(H97) differs from amygdalin by one glycoside, and its polarity is smaller than that of amygdalin. Therefore, in the chromatographic conditions we set up, the peak of prunasin was later than that of amygdalin. We confirmed the retention time of 10.88 min for prunasin after the fingerprinting of the reference substance, which is in accordance with the polar pattern of the two. Prunasin was [M+NH_4_]^+^ ion in positive ion mode, *m/z* 313.1395 (error: 0.278 ppm). In negative ion mode it was [M+HCOO]^-^ ion, *m/z* 340.1037 (error: -0.176 ppm). Similarly, based on the MS/MS data of the parent ion, we extrapolated the possible cleavage modes of prunasin in the positive and negative ion modes, respectively. In the positive ion mode, the para-molecular ion peak of prunasin was *m/z* 313.1395 ([M+NH_4_]^+^). The four fragments *m/z* 97.0289, 127.0392, 145.0495, and 163.0599 were mainly fragment ions produced by the glycoside undergoing sequential loss of H_2_O. In the negative ion mode, prunasin has a para-molecular ion peak at *m/z* 340.1037 ([M+HCOO]^-^). Subsequently, prunasin undergoes cleavage, where it loses hydrocyanic acid and undergoes ring-opening, and this structure was detected at *m/z* 245.0509. In addition, there is a signal at *m/z* 161.0445 for glycosidogenesis to complement the MS/MS information of prunasin.


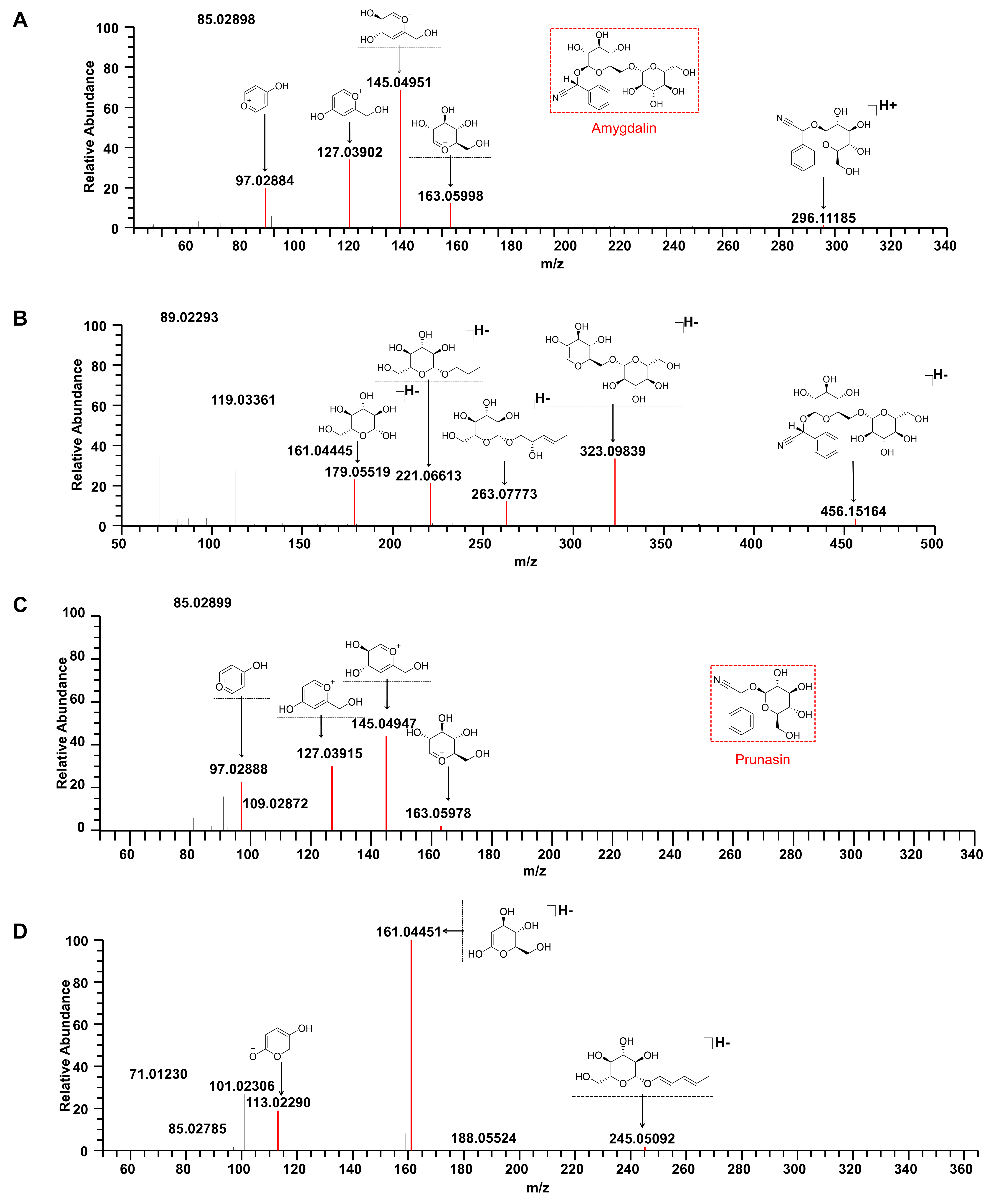


**Figure S1** Cleavage patterns of amygdalin in positive (A) and negative (B) mode; Cleavage patterns of prunasin in positive (C) and negative (D) mode.


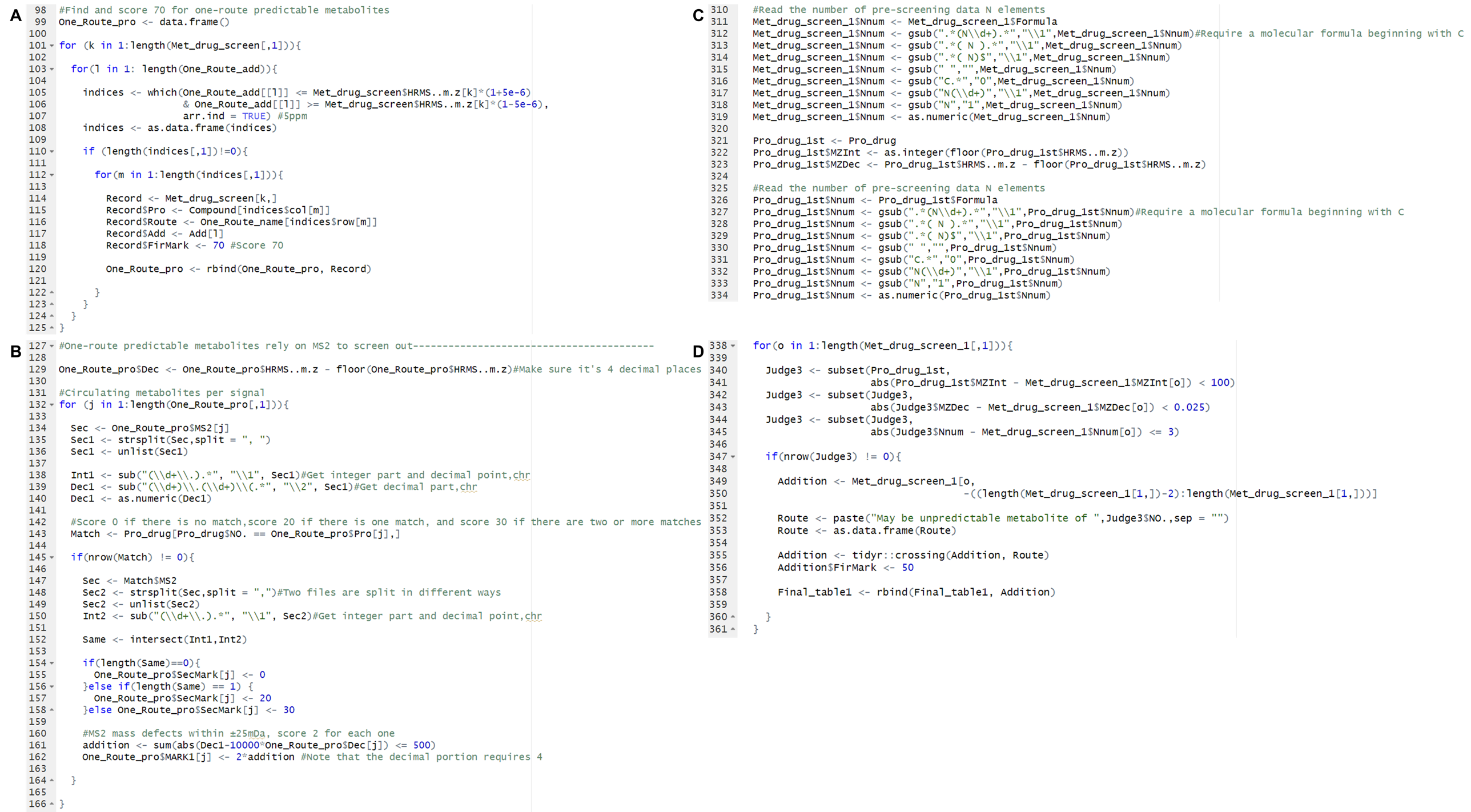


**Figure S2** MDRB arithmetic codes. (A) MS1 matching and assignment codes for One-route predictable metabolites; (B) MS2 matching and assignment codes for One-route predictable metabolites (Two-route predictable metabolites are the same); (C) Identification codes for the number of N elements; (D) MS1 and mass defect matching codes for Unpredictable metabolites.


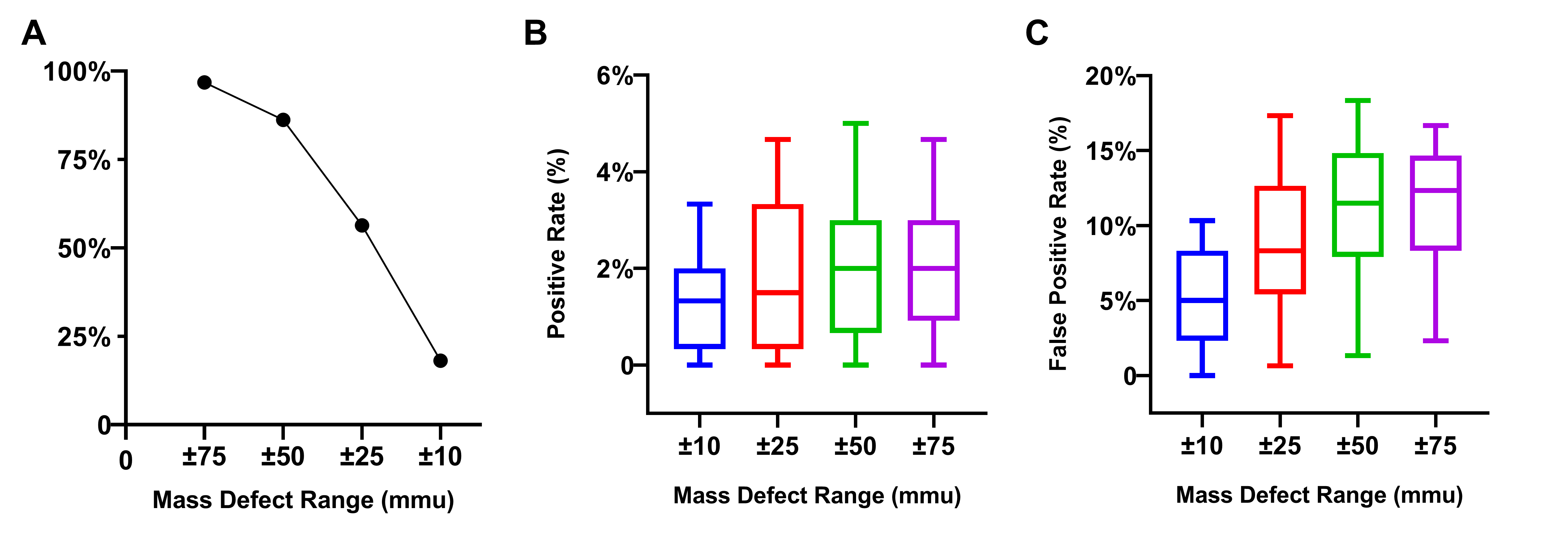


**Figure S3** (A) Proportion of known predictable metabolic pathways included at different mass defect filter ranges. (B) Positive rate and (C) false positive rate for exposed native components in mice at different mass defect filter ranges.





**Figure S4** (A) Characterization quantity and characterization rate with MDRB and without MDRB. (B) Metabolic profiles of amygdalin and prunasin based on manual characterization. (C) Metabolic profiles of amygdalin and prunasin based on MDRB. Red arrows indicate newly characterized based on MDRB.

**Table S1** Pooled plasma sample volumes at different time points of blood collection.

| Time/h | 0 | 1 | 2 | 4 | 6 | 12 | 24 |
| --- | --- | --- | --- | --- | --- | --- | --- |
| Ratio based on AUC pooled | 1 | 2 | 3 | 6 | 8 | 18 | 12 |
| Actual plasma volume/μL | 20 | 40 | 60 | 120 | 160 | 360 | 240 |

**Table S2** Mass spectrometry information of relevant components of Chao Kuxingren lyophilized powder (CKXR).

| **NO.** | **RT** | **HRMS+ m/z** | **Formula** | **Error** | **MS2** | **HRMS- m/z** | **Formula** | **Error** | **MS2** | **Name** |
| --- | --- | --- | --- | --- | --- | --- | --- | --- | --- | --- |
| H1 | 1.19 |  |  |  |  | 421.1356 | C17 H25 O12 | 1.12 | 306.0773(46) 272.0889(51) 254.0778(13) 165.0548(100) 143.0448(55) 115.0023(63) | Lamiide |
| H2 | 1.19 |  |  |  |  | 341.1089 | C12 H21 O11 | -0.13 | 341.1087(13) 179.0554(23) 161.0447(16) 119.0337(41) 101.0229(24) 89.0229(100) | Sucrose |
| H3 | 1.19 |  |  |  |  | 191.0190 | C6 H7 O7 | -4.06 | 191.0189(27) 129.0179(10) 111.0074(100) 87.0073(32) | Citric acid |
| H4 | 1.25 |  |  |  |  | 243.0621 | C9 H11 O6 N2 | -0.57 | 243.0623(16) 200.0559(35) 152.0345(18) 140.0341(16) 110.0234(100) | Uridine |
| H5 | 1.25 |  |  |  |  | 279.0390 | C11 H3 O2 N8 | 2.10 | 279.2332(13) 243.0627(100) 200.0559(59) 111.0189(50) 110.0231(39) | 2-(4,6-Dihydroxy-2-pyrimidinyl)-1H-imidazo[4,5-b]pyrazine-5,6-dicarbonitrile |
| H6 | 1.25 |  |  |  |  | 422.0880 | C22 H16 O8 N | -0.45 | 306.0761(24) 272.0893(59) 210.0886(20) 143.0445(74) 128.0343(38) 115.0022(100) | 1-Methyl-1H-Pyrrole-2,4-Dicarboxylic Acid Di-1,3-Benzodioxol-5-Ylmethyl Ester |
| H7 | 1.31 | 344.1343 | C15 H22 O8 N | 0.75 | 326.1234(67) 308.1125(87) 280.1180(100) 182.0815(57) 165.0553(37) 148.0757(34) |  |  |  |  | Phenylephrine Glucuronide |
| H8 | 1.31 | 182.0815 | C9 H12 O3 N | 1.54 | 182.0804(3) 165.0546(45) 147.0438(20) 136.0757(100) 123.0442(38) 119.0493(16) |  |  |  |  | Tyrosine |
| H9 | 1.36 | 132.1025 | C6 H14 O2 N | 1.55 | 132.1021(3) 86.0970(100) |  |  |  |  | Leucine/Isoleucine |
| H10 | 1.41 |  |  |  |  | 167.0452 | C7 H7 O3 N2 | -5.84 | 167.0451(84) 123.0550(22) 70.0283(100) | 3-Nitro-4-ethoxypyridine |
| H11 | 1.47 | 280.1655 | C14 H22 O3 N3 | -0.35 | 280.1649(100) 235.1446(56) 157.0974(55) 149.0232(93) 130.0861(98) 112.0759(20) |  |  |  |  | 2-{Methyl[(1-methyl-1H-pyrazol-5-yl)methyl]carbamoyl}cyclohexanecarboxylic acid |
| H12 | 1.50 | 294.1547 | C12 H24 O7 N | -0.23 | 276.1439(100) 258.1333(79) 248.1496(17) 230.1384(91) 212.1278(16) 132.1019(37) | 292.1405 | C12 H22 O7 N | 1.11 | 167.0445(21) 130.0861(100) | N-(1-Deoxy-1-fructosyl)leucine |
| H13 | 1.61 | 268.1039 | C10 H14 O4 N5 | -0.34 | 268.1036(5) 136.0617(100) | 312.0952 | C11 H14 O6 N5 | 0.62 | 266.0904(6) 134.0459(100) | Adenosine |
| H14 | 1.91 | 284.0990 | C10 H14 O5 N5 | 0.19 | 152.0567(100) | 282.0845 | C10 H12 O5 N5 | 0.49 | 282.0844(20) 150.0409(100) 133.0143(4) | Guanosine |
| H15 | 1.99 | 145.0497 | C6 H9 O4 | 0.79 | 145.0495(25) 127.0391(27) 99.0445(100) |  |  |  |  | 4,5-dihydroxy-5-(hydroxymethyl)cyclopent-2-en-1-one |
| H16 | 2.38 |  |  |  |  | 267.1087 | C10 H19 O8 | 0.71 | 155.0271(69) 128.1236(72) | 2,3,4,5-tetrahydroxy-6-(2-(2-hydroxyethoxy)ethoxy)hexanal |
| H17 | 2.65 | 166.0863 | C9 H12 O2 N | 0.27 | 166.0861(4) 120.0810(100) |  |  |  |  | Phenylalanine |
| H18 | 3.49 | 303.1336 | C16 H19 O4 N2 | -1.17 | 227.0666(100) 134.0602(78) 106.0656(13) |  |  |  |  | ethyl 3-carbamoyl-6-methyl-2-oxo-4-phenyl-3,4-dihydro-1H-pyridine-5-carboxylate |
| H19 | 3.49 | 393.0295 | C13 H13 O14 | -1.20 | 340.0254(29) 312.0309(19) 269.0213(23) 251.0113(39) 223.0168(40) |  |  |  |  |  |
| H20 | 3.51 | 387.0861 | C23 H15 O6 | -0.58 | 254.0333(74) 244.0492(29) 236.0231(29) 226.0384(100) 208.0277(19) |  |  |  |  | (2Z)-2-(2H-Chromen-3-ylmethylene)-3-oxo-2,3-dihydro-1-benzofuran-6-yl 2-furoate |
| H21 | 3.52 | 507.1093 | C18 H23 O15 N2 | -0.03 | 356.0467(100) 312.0308(98) 240.0191(26) 225.0072(43) |  |  |  |  |  |
| H22* | 3.59 | 407.1523 | C15 H28 O11 Na | -0.25 | 407.1522(100) |  |  |  |  | Propyl-β-gentiobioside |
| H23* | 3.61 | 402.1969 | C15 H32 O11 N | -0.14 | 163.0596(13) 145.0496(76) 127.0387(40) | 429.1615 | C16 H29 O13 | 0.32 | 383.1558(100) 221.1025(89) 161.0444(63) 113.0231(42) 101.0230(89) | Propyl-β-gentiobioside |
| H24 | 3.65 |  |  |  |  | 419.1331 | C21 H23 O9 | -3.97 | 269.1816(76) | 2-((5,7-dihydroxy-2-(4-hydroxyphenyl)chroman-3-yl)oxy)-6-methyltetrahydro-2H-pyran-3,4,5-triol |
| H25 | 3.97 | 336.1052 | C14 H19 O7 N Na | -0.63 | 336.1049(100) 174.0522(5) | 358.1140 | C15 H20 O9 N | -0.88 | 312.1092(15) 161.0445(22) 150.0549(100) 113.0230(13) 101.0229(26) | Diethyl 2-[(ethoxycarbonyl)amino]-5-methyl-3,4-furandicarboxylate |
| H26 | 3.98 | 139.0390 | C7 H7 O3 | 0.21 | 139.0391(32) 121.0286(51) |  |  |  |  | 4-Hydroxy-4,5-dihydro-6H-cyclopenta[b]furan-6-one |
| H27 | 3.98 |  |  |  |  | 457.0316 | C10 H17 O20 | -0.67 | 457.0393(15) 412.0343(57) 368.0448(59) |  |
| H28 | 3.98 |  |  |  |  | 461.1302 | C19 H25 O13 | 0.34 | 137.0232(100) 93.0331(16) | Sibiricose A3 |
| H29 | 4.02 | 649.2213 | C28 H38 O14 N2 Na | -0.33 | 336.1050(100) |  |  |  |  | (Z)-2-(acetoxymethyl)-6-(((2-ethyl-8,9-dihydroxy-1,3-dioxododecahydro-6H-benzo[e]isoindol-6-ylidene)amino)oxy)tetrahydro-2H-pyran-3,4,5-triyl triacetate |
| H30* | 5.12 | 336.1052 | C14 H19 O7 N Na | -0.63 | 336.1048(100) 174.0531(6) | 358.1144 | C15 H20 O9 N | 0.16 | 312.1091(19) 161.0445(27) 150.0549(100) 113.0231(14) 101.0230(26) | Mandelic acid amide-β-D-glucopyranoside |
| H31 | 5.53 | 605.2186 | C25 H37 O15 N2 | -0.45 | 605.2178(9) 443.1655(27) 281.1128(100) | 603.2048 | C25 H35 O15 N2 | 0.79 | 603.2093(7) 559.2176(27) 243.0776(42) 199.0870(100) 173.1075(75) 119.0336(39) | Ethyl [({(2R,4S,5R,6R)-5-acetamido-2,4-diacetoxy-6-[(1S,2R)-1,2,3-triacetoxypropyl]tetrahydro-2H-pyran-2-yl}carbonyl)amino]acetate |
| H32 | 5.82 | 547.2131 | C23 H35 O13 N2 | -0.43 | 547.2123(10) 385.1597(20) 223.1075(100) |  |  |  |  |  |
| H33 | 5.93 | 514.1318 | C23 H25 O11 N Na | -0.28 | 514.1315(100) |  |  |  |  | 5-Methyl-1-(2,3,4,6-tetra-O-acetylhexopyranosyl)-1H-indole-2,3-dione |
| H34* | 5.98 | 476.1761 | C20 H30 O12 N | -0.40 | 152.0705(23) 134.0600(100) 106.0654(16) | 520.1669 | C21 H30 O14 N | -0.48 | 474.1621(46) 312.1090(54) 263.0769(19) 161.0444(75) 150.0548(73) 101.0230(100) | Mandelic acid amide-β-gentiobioside |
| H35 | 5.98 |  |  |  |  | 619.0842 | C16 H27 O25 | -0.84 | 573.0781(45) 529.0888(100) |  |
| H36 | 5.99 | 314.1232 | C14 H20 O7 N | -0.63 | 134.0600(100) 106.0655(29) |  |  |  |  | Menisdaurin |
| H37 | 6.04 | 591.2030 | C24 H35 O15 N2 | -0.33 | 267.0963(17) 249.0865(70) 232.0602(100) 221.0918(82) 204.0657(23) 106.0653(10) | 589.1892 | C24 H33 O15 N2 | 0.97 | 589.1885(77) 474.1613(43) 161.0445(27) 150.0549(47) 119.0336(24) 114.0183(100) |  |
| H38 | 6.12 | 510.1816 | C20 H32 O14 N | -0.30 | 169.0495(100) 151.0387(13) 145.0494(21) 127.0391(14) 125.0597(17) | 491.1411 | C20 H27 O14 | 0.92 | 445.1193(2) 167.0339(100) 152.0102(12) 123.0439(4) | β-L-Fructofuranosyl 6-O-(4-hydroxy-3-methoxybenzoyl)-α-D-glucopyranoside |
| H39 | 6.21 | 205.0970 | C11 H13 O2 N2 | -0.61 | 205.0971(1) 188.0704(100) 146.0599(66) 144.0807(11) | 203.0819 | C11 H11 O2 N2 | -3.50 | 203.0819(100) 159.0916(19) 142.0652(19) 116.0491(99) | Tryptophan |
| H40 | 6.21 | 188.0705 | C11 H10 O2 N | -0.35 | 188.0703(34) 146.0599(100) 144.0806(19) 118.0653(14) |  |  |  |  | Benzylmaleimide |
| H41 | 6.24 | 367.1497 | C17 H23 O7 N2 | -0.78 | 349.1377(23) 332.1133(50) 276.1221(25) 229.0970(78) 188.0704(100) 144.0803(24) |  |  |  |  | 2'-α-mannosyl-L-tryptophan |
| H42 | 6.40 | 633.2615 | C26 H41 O14 N4 | 0.15 | 633.2586(98) 471.2073(87) 309.1551(100) 274.1186(32) 246.1239(60) |  |  |  |  | 5-Deoxy-1,2-O-isopropylidene-5-(4-{2-[(2,3,4,6-tetra-O-acetyl-β-D-glucopyranosyl)oxy]ethyl}-1H-1,2,3-triazol-1-yl)-α-D-xylofuranose |
| H43 | 6.48 | 547.2138 | C23 H35 O13 N2 | 0.81 | 547.2112(9) 385.1588(12) 223.1075(100) |  |  |  |  |  |
| H44* | 6.49 | 332.1339 | C14 H22 O8 N | -0.25 | 145.0495(35) 135.0441(40) 127.0391(25) | 313.0931 | C14 H17 O8 | 0.51 | 313.0930(9) 161.0445(28) 113.0230(50) 101.0230(100) | Mandelic acid-β-D-glucopyranoside |
| H45* | 6.69 | 494.1868 | C20 H32 O13 N | 0.01 | 163.0600(13) 145.0494(64) 135.0440(36) 127.0391(36) | 475.1458 | C20 H27 O13 | 0.08 | 475.1475(5) 431.1545(11) 269.1032(39) 161.0445(59) 113.0231(43) 101.0230(100) | Mandelic acid-β-gentiobioside |
| H46 | 6.72 |  |  |  |  | 575.0706 | C25 H19 O16 | 4.70 | 334.0008(64) 305.0014(12) 115.0286(14) | 3-{4-[2-(Carboxymethoxy)-2-oxoethoxy]phenyl}-2-(2-methoxy-2-oxoethoxy)-4-oxo-4H-chromene-6,7-dicarboxylic acid |
| H47 | 6.80 |  |  |  |  | 682.2203 | C27 H40 O19 N | 0.50 | 636.2178(58) 474.1638(48) 221.0662(39) 179.0553(66) 161.0444(61) 119.0337(51) | α-D-Galactopyranosyl-(1→3)-β-D-galactopyranosyl-(1→4)-N-(2-methoxybenzoyl)-β-D-glucopyranosylamin |
| H48 | 6.84 |  |  |  |  | 562.1779 | C23 H32 O15 N | 0.32 | 562.1770(54) 532.1671(100) 208.0611(11) 176.0707(39) 101.0227(17) |  |
| H49 | 6.85 | 561.2291 | C24 H37 O13 N2 | 0.20 | 561.2286(30) 399.1758(10) 237.1230(100) | 559.2142 | C24 H35 O13 N2 | -0.42 | 559.2148(100) 397.1633(8) 235.1083(12) 129.0659(23) 102.0545(18) |  |
| H50 | 6.90 |  |  |  |  | 353.0875 | C16 H17 O9 | -0.81 | 353.0889(4) 191.0555(100) 179.0340(65) 135.0442(17) | Isomer of chlorogenic acid |
| H51* | 6.90 | 332.1339 | C14 H22 O8 N | -0.25 | 145.0494(43) 135.0440(45) 127.0390(30) | 313.0930 | C14 H17 O8 | 0.32 | 313.0933(6) 161.0444(31) 113.0231(56) 101.0230(100) | Mandelic acid-β-D-glucopyranoside |
| H52 | 6.91 |  |  |  |  | 627.1935 | C28 H35 O16 | 0.67 | 313.0930(67) 269.1042(37) 161.0447(85) 159.0286(12) 113.0228(55) 101.0230(100) | 2-O-[(2S,3R,4S)-3,4-Dihydroxy-4-{[(4-hydroxy-3,5-diméthoxybenzoyl)oxy]méthyl}tétrahydro-2-furanyl]-β-D-glucopyranoside de 3,4-diméthoxyphényle |
| H53 | 6.96 |  |  |  |  | 590.1730 | C24 H32 O16 N | 0.53 | 590.1738(100) 546.1845(25) 221.0667(19) 186.0552(79) 161.0447(38) 115.0023(84) |  |
| H54 | 7.58 |  |  |  |  | 521.1515 | C21 H29 O15 | 0.57 | 151.0389(100) 136.0150(19) |  |
| H55 | 7.64 |  |  |  |  | 590.1734 | C24 H32 O16 N | 1.26 | 590.1729(63) 186.0553(100) 179.0552(13) 161.0447(18) 115.0022(59) |  |
| H56 | 7.78 |  |  |  |  | 487.1460 | C21 H27 O13 | 0.65 | 163.0390(100) 119.0489(22) |  |
| H57 | 7.80 | 339.0835 | C17 H16 O6 Na | -1.21 | 311.0703(39) 272.0644(100) 263.0591(15) 242.0542(93) 191.0384(44) |  |  |  |  | 4,4'-(1,3-Propanediylbis(oxy))bis-benzoic acid |
| H58 | 7.85 |  |  |  |  | 637.1988 | C26 H37 O18 | 0.40 | 637.1982(28) 431.1564(44) 269.1031(31) 161.0445(61) 119.0336(48) 101.0230(100) | Methyl 4-{[α-D-mannopyranosyl-(1→3)-[α-D-mannopyranosyl-(1→6)]-α-D-mannopyranosyl]oxy}benzoate |
| H59* | 7.93 | 494.1867 | C20 H32 O13 N | -0.22 | 163.0600(15) 145.0494(64) 135.0440(42) 127.0390(37) | 475.1454 | C20 H27 O13 | -0.62 | 475.1460(7) 431.1554(11) 269.1033(45) 161.0444(60) 113.0230(47) 101.0230(100) | Mandelic acid-β-gentiobioside |
| H60 | 7.93 | 515.1154 | C23 H24 O12 Na | -1.12 | 515.1164(71) 381.0800(55) 279.0384(100) 261.0281(21) 237.0282(37) 219.0176(25) |  |  |  |  | Aurantio-obtusin β-D-glucoside |
| H61 | 7.94 |  |  |  |  | 575.0705 | C25 H19 O16 | 4.60 | 575.0699(19) 539.0497(34) 389.0186(11) 335.0042(13) 330.0051(40) |  |
| H62 | 7.94 |  |  |  |  | 951.2989 | C40 H55 O26 | 0.20 | 475.1456(100) 431.1559(65) 269.1031(97) 161.0444(89) 113.0230(49) 101.0229(89) |  |
| H63 | 7.97 |  |  |  |  | 559.1024 | C33 H19 O9 | -1.96 | 513.0931(41) 351.0400(100) 269.1033(17) 161.0447(41) 101.0228(50) | 8,8',8''-Trihydroxy-6,6',6''-trimethyl-2,2':7',2''-ternaphthalene-1,1',1'',4,4',4''-hexone |
| H64 | 8.05 |  |  |  |  | 576.1934 | C24 H34 O15 N | -0.06 | 576.1935(11) 532.1669(100) | 5-acetamido-2-((3,5-dihydroxy-2-(hydroxymethyl)-6-(4-methoxyphenoxy)tetrahydro-2H-pyran-4-yl)oxy)-4-hydroxy-6-(1,2,3-trihydroxypropyl)tetrahydro-2H-pyran-2-carboxylic acid |
| H65 | 8.28 | 217.0972 | C12 H13 O2 N2 | 0.35 | 217.1047(23) 144.0808(100) | 215.0821 | C12 H11 O2 N2 | -2.38 | 215.0822(51) 171.0917(91) 142.0649(21) 116.0492(61) | Ethyl 5-phenyl-1H-pyrazole-3-carboxylate |
| H66 | 8.53 |  |  |  |  | 883.2248 | C21 H43 O28 N10 | -0.66 | 578.1533(60) 544.1675(11) 500.1780(28) 338.1256(13) 272.0897(67) 176.0708(100) |  |
| H67 | 8.63 |  |  |  |  | 293.1245 | C12 H21 O8 | 0.99 | 293.1243(100) 131.0701(98) 119.0336(18) 101.0229(20) | (2,2-Dimethyl-1,3-dioxolan-4-yl)methyl β-D-galactopyranoside |
| H68 | 8.85 | 536.1976 | C22 H34 O14 N | 0.35 | 195.0651(52) 177.0545(100) 145.0495(23) 127.0391(15) | 517.1562 | C22 H29 O14 | -0.17 | 193.0498(100) 178.0261(13) 134.0361(7) | (5R,6S)-1-Oxo-5-vinyl-5,6-dihydro-1H,3H-pyrano[3,4-c]pyran-6-yl 6-O-β-D-glucopyranosyl-β-D-glucopyranoside |
| H69 | 8.85 | 339.1073 | C16 H19 O8 | -0.51 | 177.0545(100) 145.0284(23) |  |  |  |  | trans-3-O-p-coumaroylquinic acid |
| H70 | 8.85 | 279.0624 | C15 H12 O4 Na | -1.22 | 279.0624(18) 237.0273(24) 177.0544(100) 149.0227(34) |  |  |  |  | Isoliquiritigenin |
| H71 | 8.90 | 485.1629 | C20 H30 O12 Na | -0.16 | 485.1628(100) 365.1046(4) |  |  |  |  |  |
| H72* | 9.02 | 355.1024 | C16 H19 O9 | 0.09 | 193.0494(100) |  |  |  |  | Neochlorogenic acid |
| H73 | 9.22 | 642.2003 | C26 H37 O16 N Na | -0.21 | 642.1989(100) 536.1608(16) 509.1463(16) 480.1498(6) |  |  |  |  |  |
| H74 | 9.31 | 526.2127 | C21 H36 O14 N | -0.61 | 185.0807(100) 153.0548(16) |  |  |  |  | (2S,3S,4R,5R,6S)-6-(((4aS,7aR)-7-(hydroxymethyl)-1,4a,5,7a-tetrahydrocyclopenta[c]pyran-1-yl)oxy)-2-((((2R,3R,4S,5S,6R)-3,4,5-trihydroxy-6-(hydroxymethyl)tetrahydro-2H-pyran-2-yl)oxy)methyl)tetrahydro-2H-pyran-2,3,4,5-tetraol |
| H75* | 9.36 | 450.1970 | C19 H32 O11 N | 0.01 | 145.0494(42) 127.0391(24) | 477.1614 | C20 H29 O13 | 0.10 | 431.1558(55) 269.1034(66) 161.0445(75) 113.0230(45) 101.0230(100) | Benzyl-β-gentiobioside |
| H76 | 9.38 | 637.2448 | C26 H41 O16 N2 | -0.39 | 163.0599(20) 145.0495(81) 127.0391(41) | 664.2098 | C27 H38 O18 N | 0.61 | 618.2081(5) 407.1202(9) 383.1192(9) 221.0663(42) 179.0552(100) 161.0446(32) | 1H-Indol-3-yl β-D-glucopyranosyl-(1→4)-β-D-glucopyranosyl-(1→4)-β-D-glucopyranoside |
| H77 | 9.38 |  |  |  |  | 467.1329 | C25 H23 O9 | -3.97 | 137.1656(78) | (3S)-6-Hydroxy-3-methyl-1,7,12-trioxo-1,2,3,4,7,12-hexahydro-8-tetraphenyl 6-deoxy-β-L-mannopyranoside |
| H78 | 9.41 | 371.1173 | C13 H23 O12 | -3.11 | 321.0482(18) 279.0386(46) 147.0645(19) 129.0545(21) 111.0444(11) |  |  |  |  | Methyl-3-O-β-D-glucopyranuronosyl-α-D-galactopyranosid |
| H79 | 9.45 |  |  |  |  | 323.1347 | C13 H23 O9 | -0.05 | 323.1354(66) 119.0337(54) 113.0231(24) 101.0229(48) | Strophanthobiose |
| H80* | 9.47 | 355.1023 | C16 H19 O9 | -0.28 | 327.0746(31) 237.0281(16) 163.0389(100) | 353.0879 | C16 H17 O9 | 0.24 | 191.0553(100) | Chlorogenic acid |
| H81 | 9.47 | 293.0995 | C13 H18 O6 Na | -0.24 | 293.1006(100) 131.0266(16) | 315.1087 | C14 H19 O8 | 0.60 | 161.0449(100) 130.0904(57) 106.0269(58) 101.0228(99) | Benzyl α-D-mannopyranoside |
| H82 | 9.50 |  |  |  |  | 359.1350 | C16 H23 O9 | 0.71 | 137.1471(18) 105.1057(20) 101.0229(18) | 3-Hydroxy-2-(4-hydroxy-3-methoxyphenyl)propyl-β-D-glucopyranosid |
| H83 | 9.53 |  |  |  |  | 578.1554 | C19 H32 O19 N | -3.46 | 578.1554(8) 500.1771(16) 338.1257(9) 176.0707(100) |  |
| H84 | 9.56 |  |  |  |  | 519.1722 | C22 H31 O14 | 0.48 | 519.1713(16) 195.0655(100) 119.0336(20) 101.0228(18) | (4aS,5R,6S)-1-Oxo-5-vinyl-4,4a,5,6-tetrahydro-1H,3H-pyrano[3,4-c]pyran-6-yl 6-O-β-D-glucopyranosyl-β-D-glucopyranoside |
| H85 | 9.92 |  |  |  |  | 492.1282 | C10 H22 O14 N9 | -2.03 | 323.0986(65) 179.0552(21) 161.0450(24) 125.0230(27) 119.0336(49) 113.0231(30) |  |
| H86 | 9.92 |  |  |  |  | 586.1130 | C17 H24 O18 N5 | 1.36 | 540.1036(64) 361.0460(24) 323.0983(19) 263.0100(21) 128.0341(51) |  |
| H87 | 10.00 | 496.1208 | C23 H23 O10 N Na | -1.20 | 496.1205(100) 324.0088(22) |  |  |  |  | 7-({6-Deoxy-4-O-methyl-3-O-[(5-methyl-1H-pyrrol-2-yl)carbonyl]-α-L-mannopyranosyl}oxy)-2-oxo-2H-chromene-4-carboxylic acid |
| H88* | 10.01 | 475.1921 | C20 H31 O11 N2 | -0.39 | 163.0599(12) 145.0495(64) 127.0391(33) | 502.1565 | C21 H28 O13 N | -0.31 | 456.1511(2) 323.0984(33) 179.0552(26) 161.0445(33) 125.0231(26) 119.0336(63) | Amygdalin |
| H89 | 10.02 |  |  |  |  | 544.1655 | C23 H30 O14 N | -1.12 | 498.1213(14) |  |
| H90 | 10.02 |  |  |  |  | 913.3101 | C25 H49 O23 N14 | 0.08 | 456.1521(6) 323.0985(57) 221.0662(30) 179.0552(34) 161.0445(41) 125.0231(34) |  |
| H91 | 10.15 |  |  |  |  | 537.1811 | C22 H33 O15 | -2.61 | 297.1137(52) 221.0675(22) 152.0475(20) 125.0231(41) 119.0337(67) 101.0230(62) | (1S,4aS,6S,7R,7aS)-1-{[6-O-(β-D-Glucopyranosyl)-β-D-glucopyranosyl]oxy}-6-hydroxy-7-methyl-1,4a,5,6,7,7a-hexahydrocyclopenta[c]pyran-4-carboxylic acid |
| H92* | 10.16 |  |  |  |  | 353.0878 | C16 H17 O9 | -0.02 | 191.0552(41) 179.0341(82) 173.0445(100) 135.0435(32) | Cryptochlorogenic acid |
| H93 | 10.22 | 339.1074 | C16 H19 O8 | -0.07 | 279.0016(16) 177.0544(100) |  |  |  |  | cis-3-O-p-coumaroylquinic acid |
| H94 | 10.35 |  |  |  |  | 537.1828 | C22 H33 O15 | 0.57 | 491.1758(12) 329.1252(37) 179.0562(14) 161.0450(65) 113.0229(52) 101.0230(100) | (1S,4aS,6S,7R,7aS)-1-{[6-O-(β-D-Glucopyranosyl)-β-D-glucopyranosyl]oxy}-6-hydroxy-7-methyl-1,4a,5,6,7,7a-hexahydrocyclopenta[c]pyran-4-carboxylic acid |
| H95 | 10.60 |  |  |  |  | 618.2044 | C26 H36 O16 N | 0.64 | 618.2037(2) 323.0995(15) 179.0550(17) 161.0445(33) 113.0230(100) |  |
| H96 | 10.82 | 441.1731 | C19 H30 O10 Na | -0.11 | 441.1726(100) |  |  |  |  |  |
| H97* | 10.85 | 313.1395 | C14 H21 O6 N2 | 0.28 | 145.0496(39) 127.0393(27) | 340.1037 | C15 H18 O8 N | -0.18 | 188.0560(5) 161.0445(100) 159.0287(6) 113.0230(21) 101.0231(23) | Prunasin |
| H98 | 10.85 | 334.0688 | C17 H13 O5 NNa | 0.68 | 118.0090(82) |  |  |  |  | 2-(3-methoxyphenyl)-4-oxo-4H-benzo[d][1,3]oxazin-6-yl acetate |
| H99 | 10.91 |  |  |  |  | 447.1510 | C19 H27 O12 | 0.52 | 401.1454(100) 269.1040(85) 161.0451(63) 131.0335(20) 113.0228(19) 101.0230(50) | 4-(Hydroxymethyl)-2-methoxyphenyl 6-O-[(2R,3R,4R)-3,4-dihydroxy-4-(hydroxymethyl)tetrahydro-2-furanyl]-β-D-glucopyranoside |
| H100 | 11.07 |  |  |  |  | 206.0817 | C11 H12 O3 N | -2.85 | 206.0812(58) 164.0707(38) | ethyl 4'-methyloxanilate |
| H101 | 11.16 |  |  |  |  | 544.1679 | C23 H30 O14 N | 1.24 | 544.1684(21) 176.0707(100) |  |
| H102 | 11.31 |  |  |  |  | 447.1513 | C19 H27 O12 | 1.14 | 401.1462(12) 269.1028(100) 161.0445(44) 101.0232(25) |  |
| H103 | 11.45 | 181.0861 | C10 H13 O3 | 1.10 | 181.0854(100) 148.0518(18) | 179.0704 | C10 H11 O3 | -5.24 | 179.0705(9) 133.0074(4) 89.1009(4) | ethyl mandelate |
| H104 | 11.45 |  |  |  |  | 567.1938 | C23 H35 O16 | 3.22 | 359.1342(36) 161.0447(62) 113.0228(37) 101.0231(100) | Methyl (1S,4aS,8S,8aS)-8-{[6-O-(β-D-glucopyranosyl)-β-D-glucopyranosyl]oxy}-3-hydroxy-1-methyl-4,4a,8,8a-tetrahydro-1H,3H-pyrano[3,4-c]pyran-5-carboxylate |
| H105 | 11.48 |  |  |  |  | 491.1776 | C21 H31 O13 | 1.15 | 221.0667(10) 179.0553(19) 161.0443(13) 121.0281(100) 119.0337(17) 101.0229(20) | 4-(1,3-Dihydroxypropyl)-2-methoxyphenyl 2-O-[(2S,3R,4R)-3,4-dihydroxy-4-(hydroxymethyl)tetrahydro-2-furanyl]-β-D-glucopyranoside |
| H106 | 11.59 |  |  |  |  | 631.2251 | C28 H39 O16 | 1.18 | 423.1686(33) 375.1448(100) 227.0921(54) 195.0656(84) 165.0547(76) | 3-Methyl-2-buten-1-yl 2,3,4-tri-O-acetyl-6-O-[(2R,3R,4S)-3,4-diacetoxy-4-(acetoxymethyl)tetrahydro-2-furanyl]-β-D-glucopyranoside |
| H107 | 11.71 | 485.1634 | C20 H30 O12 Na | 0.85 | 485.1623(100) 347.0942(9) | 507.1722 | C21 H31 O14 | 0.56 | 323.0996(52) 221.0663(34) 179.0555(26) 119.0337(85) 113.0232(44) 101.0230(67) | Forsythoside E |
| H108 | 11.96 |  |  |  |  | 634.1998 | C26 H36 O17 N | 3.15 | 377.1102(8) 191.0553(43) 149.0444(100) 131.0338(25) |  |
| H109 | 12.02 |  |  |  |  | 359.1351 | C16 H23 O9 | 0.96 | 161.0446(100) 132.1213(66) |  |
| H110 | 12.21 | 624.1924 | C28 H34 O15 N | 0.23 | 268.0806(62) 250.0704(44) 124.0395(100) 109.0287(17) 106.0290(59) |  |  |  |  | 3-(1,3-Benzodioxol-5-yl)-6-methoxy-4-oxo-4H-chromen-7-yl 6-O-[(2S,3R,4R)-3,4-dihydroxy-4-(hydroxymethyl)tetrahydro-2-furanyl]-β-D-glucopyranoside |
| H111 | 12.21 |  |  |  |  | 447.1514 | C19 H27 O12 | 1.28 | 401.1451(67) 149.0448(13) 131.0336(17) | Barlerin |
| H112 | 12.24 |  |  |  |  | 461.1671 | C20 H29 O12 | 1.37 | 415.1605(55) 269.1033(100) 161.0443(74) 101.0230(72) |  |
| H113 | 12.49 | 388.2540 | C16 H31 O N9 Na | -0.97 | 177.1121(11) 133.0859(58) |  |  |  |  |  |
| H114 | 12.62 |  |  |  |  | 493.1933 | C21 H33 O13 | 1.23 | 211.0970(100) |  |
| H115 | 12.70 |  |  |  |  | 551.1625 | C22 H31 O16 | 3.30 | 195.0659(17) 181.0495(100) |  |
| H116 | 12.73 |  |  |  |  | 307.1401 | C13 H23 O8 | 0.78 | 307.1386(94) 305.0033(11) 145.0859(100) 119.0336(19) 101.0228(13) | 5-(hydroxymethyl)-2,3,7-trimethoxy-2,3-dimethylhexahydro-5H-pyrano[3,4-b][1,4]dioxin-8-ol |
| H117 | 13.07 | 290.0910 | C17 H12 O2 N3 | -4.94 | 283.0836(53) 237.0280(100) 219.0181(51) 203.0644(28) 181.0860(35) 163.0750(69) |  |  |  |  |  |
| H118 | 13.07 | 331.1538 | C19 H23 O5 | -0.48 | 287.1273(21) 255.1012(42) 227.1062(43) 189.0909(46) 151.0754(100) 137.0597(68) |  |  |  |  | 3-{4,8-Dimethyl-7-[(3-methyl-2-buten-1-yl)oxy]-2-oxo-2H-chromen-3-yl}propanoic acid |
| H119 | 13.08 | 563.2101 | C26 H36 O12 Na | 0.32 | 563.2099(100) 401.1564(54) | 585.2195 | C27 H37 O14 | 3.00 | 377.1609(8) 329.1396(100) 314.1165(5) 195.0655(11) 165.0546(10) | 4-{(1R,2S)-1,3-Dihydroxy-2-[4-(3-hydroxypropyl)-2-methoxyphenoxy]propyl}-2-methoxyphenyl β-D-glucopyranoside |
| H120 | 13.09 |  |  |  |  | 575.1908 | C32 H31 O10 | -2.45 | 125.1021(100) |  |
| H121 | 13.13 | 589.2603 | C26 H41 O13 N2 | -0.01 | 589.2557(14) 427.2077(26) 265.1544(100) |  |  |  |  |  |
| H122 | 13.13 | 509.1050 | C24 H22 O11 Na | -0.95 | 254.0477(100) 238.0530(28) 210.0580(17) 208.0426(51) 190.0321(67) 162.0375(28) |  |  |  |  | (2R,3R)-5,7-Dihydroxy-2-(4-hydroxy-3,5-dimethoxyphenyl)-3,4-dihydro-2H-chromen-3-yl 3,4,5-trihydroxybenzoate |
| H123 | 13.19 |  |  |  |  | 715.2465 | C32 H43 O18 | 1.44 | 715.2529(6) 391.1391(51) 343.1200(25) 195.0653(100) 181.0498(29) 165.0544(53) |  |
| H124 | 13.55 |  |  |  |  | 553.1935 | C26 H33 O13 | 3.46 | 391.1406(23) 343.1187(100) 195.0656(39) 165.0549(17) | Methyl (4aS,5R,7S,7aS)-1-(β-D-glucopyranosyloxy)-7-hydroxy-5-{[3-(4-hydroxyphenyl)propanoyl]oxy}-7-methyl-1,4a,5,6,7,7a-hexahydrocyclopenta[c]pyran-4-carboxylate |
| H125 | 13.56 | 563.2100 | C26 H36 O12 Na | 0.22 | 563.2105(100) 401.1561(47) | 585.2197 | C27 H37 O14 | 3.23 | 329.1406(91) 195.0663(100) 165.0552(82) |  |
| H126 | 13.75 | 181.0860 | C10 H13 O3 | 0.33 | 181.0857(100) 148.0518(15) |  |  |  |  | ethyl mandelate |
| H127 | 14.56 | 432.2804 | C19 H38 O6 N5 | -3.01 | 177.1120(11) 133.0859(55) |  |  |  |  |  |
| H128 | 14.58 | 415.2535 | C17 H36 O6 N4 Na | 1.79 | 133.0860(40) |  |  |  |  |  |
| H129 | 14.62 | 681.3089 | C34 H49 O14 | -4.08 | 443.1887(14) 405.0984(11) 259.0932(18) 241.0813(18) 157.0971(82) 129.1025(100) | 679.2952 | C34 H47 O14 | -1.18 | 679.2956(17) 635.2675(43) 332.1253(90) 301.1538(15) 185.0563(45) 128.0340(100) | (3β)-3-{[4-O-(β-D-Glucopyranosyl)-β-D-xylopyranosyl]oxy}-14-hydroxy-19-oxocarda-4,20(22)-dienolide |
| H130 | 15.77 | 623.2445 | C29 H39 O13 N2 | -0.25 | 623.2412(13) 461.1902(25) 299.1386(100) |  |  |  |  | 6-({3-[(2-Hydroxyethyl)amino]-3-oxopropyl}carbamoyl)-3a,4,5,7a-tetrahydro-1,3-benzodioxol-4-yl 4-[3-(hexopyranosyloxy)-1-propen-1-yl]benzoate |
| H131 | 16.65 |  |  |  |  | 605.2457 | C27 H41 O15 | 2.86 | 605.2454(100) 221.0658(14) 161.0445(15) 119.0336(18) 101.0229(30) |  |
| H132 | 17.14 | 476.3067 | C21 H42 O7 N5 | -2.47 | 177.1124(11) 133.0859(51) |  |  |  |  | 1-N-Ethylsisomicin |
| H133 | 17.19 |  |  |  |  | 615.2303 | C28 H39 O15 | 3.16 | 359.1492(100) 195.0658(69) 165.0550(43) | asperuloide B |
| H134 | 18.31 |  |  |  |  | 529.1935 | C24 H33 O13 | 1.54 | 205.0863(100) 161.0956(13) | (2E)-4-(6-{[6-O-(6-Deoxy-α-L-mannopyranosyl)-β-D-glucopyranosyl]oxy}-3-hydroxy-2-methylphenyl)-2-methyl-2-butenoic acid |
| H135 | 18.41 |  |  |  |  | 443.1929 | C21 H31 O10 | 1.49 | 443.1895(11) 161.0440(7) 119.0334(52) 113.0230(44) 101.0229(88) | dihydrophaseic acid 4-O-β-D-glucoside |
| H136 | 18.51 |  |  |  |  | 693.3120 | C35 H49 O14 | -1.20 | 693.3123(31) 649.2858(37) 332.1252(93) 223.1079(24) 185.0558(40) 128.0340(100) |  |
| H137 | 18.52 |  |  |  |  | 521.2040 | C26 H33 O11 | 2.31 | 329.1394(100) 175.0755(9) | Urolignoside |
| H138 | 18.55 | 331.1546 | C19 H23 O5 | 1.90 | 331.1726(24) 285.1117(24) 255.1014(38) 227.1065(48) 189.0910(34) 151.0754(100) |  |  |  |  |  |
| H139 | 18.55 |  |  |  |  | 377.1616 | C20 H25 O7 | 2.77 | 329.1397(100) 314.1161(50) 195.0657(11) 165.0547(37) 149.0232(14) | 1-(4-hydroxy-3-methoxyphenyl)-2-(4-(3-hydroxypropyl)-2-methoxyphenoxy)propane-1,3-diol |
| H140 | 18.59 |  |  |  |  | 367.1980 | C16 H31 O9 | 1.67 | 367.1972(100) 330.1985(3) |  |
| H141 | 18.61 | 563.1879 | C29 H32 O10 Na | -1.49 | 268.0812(91) 250.0707(75) 124.0394(100) 109.0288(29) 106.0291(61) |  |  |  |  |  |
| H142 | 18.61 |  |  |  |  | 623.1997 | C29 H35 O15 | 2.42 | 623.2002(51) 461.1669(11) 161.0234(100) | verbascoside |
| H143 | 18.69 |  |  |  |  | 391.1409 | C20 H23 O8 | 2.71 | 343.1187(100) 195.0655(48) 165.0546(38) 151.0384(13) |  |
| H144 | 18.71 | 171.1493 | C9 H19 O N2 | 0.76 | 171.1491(100) 89.0715(79) |  |  |  |  | 2-(1-Piperazinyl)cyclopentanol |
| H145 | 18.71 |  |  |  |  | 261.1349 | C12 H21 O6 | 1.91 | 261.1346(14) 187.0967(100) 125.0959(58) | ((4R,4aR,8R,8aR)-2,2,6,6-tetramethyltetrahydro-[1,3]dioxino[5,4-d][1,3]dioxine-4,8-diyl)dimethanol |
| H146 | 18.71 |  |  |  |  | 411.2245 | C18 H35 O10 | 2.24 | 411.2241(100) 105.0544(25) |  |
| H147 | 18.74 | 340.2593 | C18 H34 O3 N3 | -0.38 | 322.2482(9) 171.1491(78) 114.0916(100) |  |  |  |  | N-Cyclohexyl-4-{3-[(2-methoxyethyl)amino]-3-oxopropyl}-1-piperidinecarboxamide |
| H148 | 18.74 |  |  |  |  | 661.2853 | C34 H45 O13 | -1.85 | 661.2831(33) 617.2551(28) 389.1454(24) 331.1409(81) 274.1189(25) 184.0721(26) |  |
| H149 | 18.76 |  |  |  |  | 425.2041 | C18 H33 O11 | 2.91 | 425.2008(100) 367.1971(85) 323.1719(17) 189.1563(4) | Hexyl β-D-maltoside |
| H150 | 18.78 |  |  |  |  | 455.2508 | C20 H39 O11 | 2.21 | 455.2497(100) 149.0804(7) 105.0542(24) 101.0231(17) |  |
| H151 | 18.78 |  |  |  |  | 483.2090 | C20 H35 O13 | 1.38 | 483.2100(18) 251.1146(20) 163.0604(46) 119.0338(73) 105.0543(38) 101.0229(41) |  |
| H152* | 18.81 |  |  |  |  | 187.0969 | C9 H15 O4 | -3.59 | 187.0969(100) 143.1064(20) 125.0958(98) | Azelaic acid |
| H153 | 18.82 |  |  |  |  | 407.1718 | C21 H27 O8 | 1.64 | 359.1495(70) 344.1256(41) 195.0655(38) 165.0547(100) 150.0312(23) |  |
| H154 | 19.08 | 171.1492 | C9 H19 O N2 | -0.23 | 171.1491(100) 89.0714(78) |  |  |  |  | 2-(1-Piperazinyl)cyclopentanol |
| H155 | 19.09 |  |  |  |  | 201.1128 | C10 H17 O4 | -2.25 | 201.1125(100) 183.1022(16) 139.1116(74) | (4R,5S)-5-Butyl-5-ethoxy-4-hydroxydihydro-2(3H)-furanone |
| H156 | 19.12 | 264.2317 | C17 H30 O N | -2.01 | 219.1742(100) |  |  |  |  | 2,6-Di-tert-butyl-4-[(dimethylamino)methyl]phenol |
| H157 | 19.17 | 317.1313 | C11 H22 O7 N2 Na | -1.90 | 317.1319(71) 151.0324(100) 149.0958(17) 131.0855(19) 109.0652(35) 107.0858(48) |  |  |  |  | (1R,2R,3S,5R,6S)-3,5-Diamino-2,6-dihydroxycyclohexyl β-D-ribofuranoside |
| H158 | 19.27 |  |  |  |  | 242.1761 | C13 H24 O3 N | -0.19 | 242.1760(100) 225.1493(36) 181.1588(17) |  |
| H159 | 19.28 | 274.2737 | C16 H36 O2 N | -1.37 | 274.2737(100) 256.2631(6) |  |  |  |  | 2,2'-(Dodecylimino)diethanol |
| H160 | 19.29 | 318.2998 | C18 H40 O3 N | -1.57 | 318.3001(100) 256.2632(59) |  |  |  |  |  |
| H161 | 19.53 | 228.1954 | C13 H26 O2 N | -1.95 | 175.1477(44) 133.1012(18) 119.0856(15) 109.1015(61) |  |  |  |  |  |
| H162 | 19.62 | 225.1960 | C13 H25 O N2 | -0.84 | 225.1959(100) 100.1125(46) |  |  |  |  |  |
| H163 | 19.63 | 207.1019 | C12 H15 O3 | 1.64 | 207.1017(2) 121.0650(100) |  |  |  |  | ethyl (E)-3-(4-methoxyphenyl)acrylate |
| H164 | 19.83 | 209.1535 | C13 H21 O2 | -0.65 | 209.1535(100) 153.1278(35) 135.1175(21) 125.0967(23) | 207.1385 | C13 H19 O2 | -2.86 | 207.1384(100) | α-inone |
| H165 | 19.87 | 415.2109 | C24 H31 O6 | -1.39 | 119.0857(100) |  |  |  |  |  |
| H166 | 19.91 |  |  |  |  | 249.1496 | C15 H21 O3 | -0.15 | 249.1495(100) 205.1592(17) | 5-(2,5-dimethylphenoxy)-2,2-dimethylpentanoic acid |
| H167 | 19.98 | 241.1731 | C5 H21 O3 N8 | -0.14 | 241.1733(100) 100.1125(70) | 239.1585 | C5 H19 O3 N8 | -0.42 | 239.1583(70) 195.1384(100) 154.0624(27) |  |
| H168 | 19.99 |  |  |  |  | 275.1357 | C10 H19 O5 N4 | -1.61 | 275.1680(100) 204.0858(8) | 11-Hydroxy-2,11-dimethyl-3,8-dioxo-2,4,7,9-tetraazadodecan-12-oic acid |
| H169 | 20.02 |  |  |  |  | 476.2784 | C25 H38 O6 N3 | 3.66 | 476.2789(4) 279.2330(100) 196.0373(11) | Bis[1-(diisopropylamino)-1-oxo-2-propanyl] 2,6-pyridinedicarboxylate |
| H170 | 20.04 |  |  |  |  | 313.2387 | C18 H33 O4 | 0.69 | 313.2383(100) 185.1183(10) | Isoleukotoxindiol |
| H171 | 20.05 | 520.3397 | C31 H47 O4 N Na | -0.02 | 520.3403(5) 184.0732(100) 104.1073(66) |  |  |  |  | (3α,5β,7β,8ξ)-3,7-Dihydroxy-N-(4-methoxyphenyl)cholan-24-amide |
| H172 | 20.11 |  |  |  |  | 293.2124 | C18 H29 O3 | 1.03 | 293.2132(100) 275.2018(38) 249.1859(19) 221.1542(10) 193.1584(66) 183.1382(13) | Octahydrospiro[benzo[c]chromene-6,1'-cyclohexane]-4a,10a(2H,6aH)-diol |
| H173 | 20.18 |  |  |  |  | 452.2785 | C23 H38 O6 N3 | 4.18 | 452.2793(6) 255.2329(100) | N-[(trans-4-{[(1-{[(2-Methyl-2-propanyl)oxy]carbonyl}-L-prolyl)amino]methyl}cyclohexyl)carbonyl]-L-valine |
| H174 | 20.19 | 200.2009 | C12 H26 O N | -0.11 | 200.2008(100) 159.1174(15) 145.1017(25) |  |  |  |  |  |
| H175 | 20.23 |  |  |  |  | 257.1546 | C17 H21 O2 | -0.40 | 257.1546(100) 83.0485(7) | Geranyl Benzoate |
| H176 | 20.25 |  |  |  |  | 313.1811 | C20 H25 O3 | 0.45 | 313.1812(100) 269.1918(4) | 2,7-Dimethyl-2-(4-methyl-3-penten-1-yl)-2H-chromen-5-ol |
| H177 | 20.26 | 291.1950 | C18 H27 O3 | -1.58 | 291.1960(23) 249.1846(100) |  |  |  |  | (2,4-Di-tert-pentylphenoxy)acetic acid |
| H178 | 20.31 |  |  |  |  | 478.2940 | C25 H40 O6 N3 | 3.72 | 478.2941(7) 281.2486(100) 196.0372(9) |  |
| H179 | 20.31 |  |  |  |  | 295.2278 | C18 H31 O3 | -0.10 | 295.2278(100) 277.2175(43) 195.1382(26) | Hydroxy-octadecatrienoic acid |
| H180 | 20.39 | 522.3552 | C31 H49 O4 N Na | -0.42 | 522.3552(4) 504.3447(4) 184.0732(100) 104.1073(72) |  |  |  |  | (3β)-N,3-Dihydroxy-N-methyl-11-oxoolean-12-en-30-amide |
| H181 | 20.44 |  |  |  |  | 233.1543 | C15 H21 O2 | -1.82 | 233.1543(100) |  |
| H182 | 20.45 |  |  |  |  | 293.2123 | C18 H29 O3 | 0.42 | 293.2120(100) 249.2220(4) 113.0958(18) | 2-{2-[4-(1,1,3,3-Tetramethylbutyl)Phenoxy]Ethoxy}Ethanol |
| H183 | 20.73 | 279.1587 | C16 H23 O4 | -1.31 | 279.0930(100) 219.0567(17) 149.0232(63) |  |  |  |  |  |
| H184 | 20.74 | 297.2417 | C18 H33 O3 | -2.33 | 279.2318(29) 183.1376(39) 147.1170(58) 135.1168(19) 121.1013(20) | 295.2279 | C18 H31 O3 | 0.01 | 295.2278(100) 277.2176(5) 195.1383(6) | [1,1':3',1''-tercyclohexane]-2,2',2''-triol |
| H185 | 20.76 | 273.1847 | C18 H25 O2 | -0.90 | 273.1846(93) 255.1741(100) | 271.1704 | C18 H23 O2 | 0.21 | 271.2280(100) 225.2214(17) | Estradiol |
| H186 | 20.76 |  |  |  |  | 347.1715 | C16 H27 O8 | 0.98 | 301.1651(100) 226.0160(65) |  |
| H187 | 20.93 |  |  |  |  | 299.2016 | C20 H27 O2 | -0.18 | 299.2019(100) |  |
| H188 | 21.07 | 200.2008 | C12 H26 O N | -0.55 | 200.2005(78) 199.1803(46) 100.0760(33) |  |  |  |  |  |
| H189 | 21.67 |  |  |  |  | 339.2325 | C23 H31 O2 | -1.22 | 339.2328(100) 163.1117(83) | 2,2'-Methylenebis[4-methyl-6-(2-methyl-2-propanyl)phenol] |
| H190* | 21.84 |  |  |  |  | 279.2328 | C18 H31 O2 | -0.69 | 279.2330(100) | Linoleic acid |
| H191 | 22.34 |  |  |  |  | 374.1321 | C13 H20 O8 N5 | 0.84 | 374.1322(100) 338.1552(69) |  |
| H192* | 22.34 |  |  |  |  | 255.2327 | C16 H31 O2 | -0.88 | 255.2329(100) | Palmitic acid |
| H193 | 22.48 |  |  |  |  | 381.1738 | C12 H25 O8 N6 | -0.43 | 381.1740(86) |  |
| H194 | 22.48 |  |  |  |  | 400.1476 | C15 H22 O8 N5 | 0.64 | 400.1487(100) 364.1717(63) |  |
| H195* | 22.48 |  |  |  |  | 281.2485 | C18 H33 O2 | -0.48 | 281.2487(100) | Oleic acid |
| H196 | 23.36 |  |  |  |  | 283.2641 | C18 H35 O2 | -0.54 | 283.2643(100) |  |
| H197 | 25.11 |  |  |  |  | 595.2893 | C34 H43 O9 | -3.37 | 595.2889(54) 515.2902(12) 415.2257(6) 279.2329(100) |  |
| H198 | 25.17 |  |  |  |  | 571.2893 | C32 H43 O9 | -3.41 | 571.2891(69) 391.2241(9) 255.2328(100) |  |
| H199 | 25.46 |  |  |  |  | 297.1185 | C11 H21 O9 | -1.94 | 297.1188(42) |  |

*Components validated by reference substances

**Table S3** Mass spectrometry information of components of CKXR *in vivo*. (P: Plasma; U: Urine; L: Lung; F: Feces)

| **No.** | **RT** | **HRMS+ m/z** | **Formule** | **Error** | **MS2** | **HRMS- m/z** | **Formule** | **Error** | **MS2** | **P** | **U** | **L** | **F** |
| --- | --- | --- | --- | --- | --- | --- | --- | --- | --- | --- | --- | --- | --- |
| M1 | 1.23 | 266.1499 | C13 H20 O3 N3 | 0.05 | 266.1498(51) 239.1499(32) 209.1396(16) 150.0774(40) 122.0714(71) |  |  |  |  | - | + | - | - |
| M2 | 1.41 |  |  |  |  | 167.0452 | C7 H7 O3 N2 | -5.84 | 167.0451(84) 123.0550(22) 70.0283(100) | - | - | - | + |
| M3 | 1.47 | 280.1655 | C14 H22 O3 N3 | -0.35 | 280.1649(100) 235.1446(56) 157.0974(55) 149.0232(93) 130.0861(98) 112.0759(20) | 278.1511 | C14 H20 O3 N3 | 0.38 | 278.1505(4) 172.1080(100) 155.0812(4) | + | + | + | + |
| M4 | 1.64 | 306.1554 | C13 H24 O7 N | 0.63 | 306.1562(1) 164.0817(100) 144.0476(36) 122.0714(22) |  |  |  |  | - | + | - | - |
| M5 | 1.73 | 151.0867 | C8 H11 O N2 | 0.67 | 151.0865(47) 134.0598(5) 106.0655(22) |  |  |  |  | - | + | - | + |
| M6 | 1.75 |  |  |  |  | 357.1303 | C15 H21 O8 N2 | -0.17 | 311.1269(6) 276.1990(5) 161.0446(100) 113.0231(52) 101.0228(66) | + | + | - | - |
| M7 | 1.83 | 247.0825 | C10 H15 O7 | 1.23 | 204.0767(100) 187.0499(30) 161.0708(32) 133.0759(27) 118.0652(12) |  |  |  |  | - | + | - | - |
| M8 | 2.04 | 357.1653 | C16 H25 O7 N2 | -0.97 | 357.1653(33) 195.1126(77) 180.0878(100) |  |  |  |  | + | + | + | - |
| M9 | 2.13 | 306.1554 | C13 H24 O7 N | 0.63 | 306.1546(1) 164.0818(100) 144.0477(34) 122.0714(24) |  |  |  |  | - | + | - | - |
| M10 | 2.17 | 442.2184 | C20 H32 O8 N3 | -0.09 | 442.2174(49) 280.1651(100) 235.1435(14) 157.0970(31) 130.0863(29) | 440.2039 | C20 H30 O8 N3 | 0.19 | - | + | + | + | - |
| M11 | 2.29 | 264.1704 | C14 H22 O2 N3 | -0.27 | 264.1707(61) 219.1490(33) 191.0449(15) 130.0863(85) 127.0867(21) 122.0714(26) | 262.1560 | C14 H20 O2 N3 | -0.46 | 262.1558(100) 145.0971(42) 116.0489(17) | + | + | - | - |
| M12 | 2.29 |  |  |  |  | 357.0679 | C11 H17 O13 | 1.22 | 357.0674(100) 195.0661(51) 177.0548(14) 165.0545(16) 161.0445(41) 113.0229(17) 101.0229(43) | - | + | - | - |
| M13 | 2.38 | 604.2715 | C26 H42 O13 N3 | 0.27 | 604.2708(62) 280.1652(100) 235.1435(11) 157.0970(21) 145.0495(14) 130.0864(27) |  |  |  |  | - | + | - | - |
| M14 | 2.49 | 265.0930 | C10 H17 O8 | 1.17 | 247.0816(2) 230.0558(15) 204.0766(33) 162.0548(14) 134.0600(16) 105.0338(31) | 263.0785 | C10 H15 O8 | 4.86 | 203.0455(26) 160.0391(100) 157.0355(63) 114.0295(82) | + | + | - | - |
| M15 | 2.51 | 247.0825 | C10 H15 O7 | 1.23 | 230.0558(40) 204.0766(100) 187.0501(27) 161.0708(26) 133.0761(21) |  |  |  |  | - | + | - | - |
| M16 | 2.51 | 529.1789 | C22 H25 O8 N8 | -0.10 | 491.1006(1) 247.0821(11) 230.0559(23) 204.0766(45) 134.0600(15) |  |  |  |  | - | + | - | - |
| M17 | 2.74 | 286.1287 | C13 H20 O6 N | 0.15 | 153.0407(5) 118.0653(6) 106.0654(100) | 330.1195 | C14 H20 O8 N | 0.12 | 198.0399(39) 169.0497(28) 159.0535(28) 157.0352(80) | - | + | - | - |
| M18 | 3.01 | 357.1654 | C16 H25 O7 N2 | -0.53 | 357.1651(21) 145.0493(1) 127.0388(1) | 401.1565 | C17 H25 O9 N2 | -0.16 | 355.1534(10) 193.0972(100) 161.0441(54) 113.0229(29) 111.0185(39) | + | + | + | - |
| M19 | 3.17 | 285.1344 | C14 H21 O6 | 3.98 | 127.0867(17) |  |  |  |  | - | + | - | - |
| M20 | 3.19 | 371.1448 | C16 H23 O8 N2 | -0.05 | 371.1438(3) 209.0919(100) 191.0815(8) 145.0759(9) | 369.1304 | C16 H21 O8 N2 | 0.08 | 369.1299(36) 113.0233(17) 101.0227(58) | - | + | + | - |
| M21 | 3.20 | 335.1713 | C15 H27 O8 | 1.29 | 335.1719(5) 229.1293(73) 202.0974(12) 116.0708(25) 114.0664(100) | 333.1565 | C15 H25 O8 | 3.00 | 333.1005(1) 296.0999(7) 227.1145(100) 164.0566(13) 112.0502(53) | + | + | - | - |
| M22 | 3.22 |  |  |  |  | 227.1145 | C9 H15 O3 N4 | -2.09 | 227.1143(100) 148.0390(60) 112.0502(98) | + | + | - | - |
| M23 | 3.26 | 220.1082 | C11 H14 O2 N3 | 0.12 | 220.1082(1) 114.0664(100) | 218.0931 | C11 H12 O2 N3 | -2.02 | 218.0929(5) 113.0536(3) 112.0502(100) | - | + | - | - |
| M24 | 3.31 | 439.2087 | C21 H31 O8 N2 | 2.84 | 220.1079(13) 114.0664(100) |  |  |  |  | - | + | - | - |
| M25 | 3.33 | 271.1194 | C13 H19 O6 | 1.81 | 254.0921(100) 226.0974(34) 108.0560(93) | 269.1044 | C13 H17 O6 | 4.97 | 269.1050(1) 251.0934(2) 151.0613(100) 108.0554(2) 106.0396(16) | - | + | - | - |
| M26 | 3.34 | 517.2154 | C22 H29 O7 N8 | 0.02 | 220.1079(3) |  |  |  |  | - | + | - | - |
| M27 | 3.38 | 337.1866 | C16 H25 O4 N4 | -1.25 | 337.1080(46) 174.0521(4) 157.0760(100) |  |  |  |  | - | + | - | - |
| M28 | 3.49 | 303.1336 | C16 H19 O4 N2 | -1.17 | 227.0666(100) 152.0708(5) 134.0602(78) 106.0656(13) |  |  |  |  | + | + | - | - |
| M29 | 3.49 | 393.0295 | C13 H13 O14 | -1.20 | 340.0254(29) 312.0309(19) 269.0213(23) 251.0113(39) 223.0168(40) |  |  |  |  | + | + | - | - |
| M30 | 3.51 | 387.0861 | C23 H15 O6 | -0.58 | 254.0333(74) 244.0492(29) 236.0231(29) 226.0384(100) 208.0277(19) |  |  |  |  | + | + | - | - |
| M31 | 3.52 | 507.1093 | C18 H23 O15 N2 | -0.03 | 356.0467(100) 312.0308(98) 240.0191(26) 225.0072(43) |  |  |  |  | + | + | - | - |
| M32 | 3.53 | 359.0535 | C21 H11 O6 | -4.14 | 226.0005(16) |  |  |  |  | + | + | - | - |
| M33 | 3.56 | 421.1270 | C16 H25 O9 N2 S | -1.30 | 259.0744(100) 241.0639(17) |  |  |  |  | + | + | + | - |
| M34 | 3.58 | 388.1502 | C19 H22 O6 N3 | -0.42 | 156.0766(100) 110.0716(39) |  |  |  |  | - | + | - | - |
| M35 | 3.71 | 371.1449 | C16 H23 O8 N2 | -0.06 | 209.0919(100) |  |  |  |  | + | + | + | - |
| M36 | 3.86 | 543.1566 | C20 H31 O17 | 1.04 | 504.0957(56) 459.0743(49) 342.0425(20) 305.5721(100) 152.0704(19) |  |  |  |  | - | + | - | - |
| M37 | 3.91 |  |  |  |  | 462.1405 | C22 H24 O10 N | -0.21 | 377.1268(1) 149.0232(100) 121.0281(10) | - | + | - | - |
| M38 | 3.95 | 490.1563 | C20 H28 O13 N | 1.50 | 370.0803(10) 333.0965(28) 252.0702(100) 134.0604(10) 106.0655(3) |  |  |  |  | - | + | - | - |
| M39 | 3.97 | 336.1052 | C14 H19 O7 N Na | -0.63 | 336.1049(100) 174.0522(5) | 358.1140 | C15 H20 O9 N | -0.88 | 312.1092(15) 161.0445(22) 150.0549(100) 113.0230(13) 101.0229(26) | + | + | + | + |
| M40 | 3.97 |  |  |  |  | 374.1086 | C15 H20 O10 N | -1.68 | 328.1063(3) 166.0499(8) 150.0555(2) | + | + | - | - |
| M41 | 3.98 |  |  |  |  | 457.0316 | C10 H17 O20 | -0.67 | 457.0393(15) 412.0343(57) 368.0448(59) | + | + | + | - |
| M42 | 4.00 | 681.1582 | C20 H33 O22 N4 | 0.15 | 368.0418(100) 350.0314(14) 302.0105(17) 278.0104(19) 224.0002(15) |  |  |  |  | + | + | - | - |
| M43 | 4.02 | 649.2213 | C28 H38 O14 N2 Na | -0.33 | 336.1050(100) |  |  |  |  | + | + | + | - |
| M44 | 4.17 | 273.1339 | C13 H21 O6 | 2.47 | 273.1348(2) 228.1130(39) 110.0715(100) |  |  |  |  | - | + | - | - |
| M45 | 4.40 | 533.2250 | C25 H33 O9 N4 | 1.45 | 220.1078(15) 114.0664(100) |  |  |  |  | - | + | - | - |
| M46 | 4.50 | 337.1868 | C16 H25 O4 N4 | -0.69 | 337.1088(20) 118.0864(100) |  |  |  |  | - | + | - | - |
| M47 | 4.52 | 439.2087 | C21 H31 O8 N2 | 2.84 | 220.1082(12) 114.0664(100) |  |  |  |  | - | + | - | - |
| M48 | 4.55 | 220.1082 | C11 H14 O2 N3 | 0.12 | 220.1062(1) 114.0664(100) | 218.0931 | C11 H12 O2 N3 | -2.02 | 218.0909(3) 146.0812(2) 129.0168(3) 112.0502(100) | - | + | - | - |
| M49 | 4.57 | 505.2040 | C21 H33 O12 N2 | 2.39 | 244.1658(1) 220.1077(13) 154.0609(11) 114.0664(100) |  |  |  |  | - | + | - | - |
| M50 | 4.59 |  |  |  |  | 369.1302 | C16 H21 O8 N2 | -0.40 | 369.1290(3) 101.0342(3) | - | + | - | - |
| M51 | 4.69 |  |  |  |  | 361.1142 | C15 H21 O10 | 0.58 | 341.1274(3) 315.1154(4) 153.0545(100) 146.0235(42) | - | + | - | - |
| M52 | 4.89 | 261.1960 | C16 H25 O N2 | -0.73 | 261.1957(7) 243.1854(12) 127.1231(54) |  |  |  |  | - | + | - | - |
| M53 | 4.93 |  |  |  |  | 457.0328 | C10 H17 O20 | 2.00 | 412.0343(34) 368.0434(63) 305.0008(14) | - | + | + | + |
| M54 | 5.08 | 627.2399 | C28 H39 O14 N2 | 0.56 | 278.1941(1) 152.0704(27) 134.0600(100) 106.0654(14) |  |  |  |  | - | + | - | - |
| M55 | 5.09 |  |  |  |  | 388.1255 | C16 H22 O10 N | 1.55 | 312.1090(6) 161.0443(2) 113.0225(2) 101.0232(2) | - | + | - | - |
| M56 | 5.10 | 649.2213 | C26 H33 O12 N8 | 0.01 | 336.1048(100) |  |  |  |  | + | + | - | - |
| M57 | 5.12 | 336.1052 | C14 H19 O7 N Na | -0.63 | 336.1048(100) 174.0531(6) | 358.1144 | C15 H20 O9 N | 0.16 | 312.1091(19) 161.0445(27) 150.0549(100) 113.0231(14) 101.0230(26) | + | + | + | - |
| M58 | 5.17 | 239.2118 | C14 H27 O N2 | -0.03 | 239.2110(3) 221.2013(14) 138.0581(24) 127.1230(50) 109.0649(10) |  |  |  |  | - | + | - | - |
| M59 | 5.19 | 273.1339 | C13 H21 O6 | 2.47 | 273.1343(59) 255.1236(12) 227.1288(18) 127.0503(100) 101.0600(10) |  |  |  |  | - | + | - | - |
| M60 | 5.21 | 441.1867 | C20 H29 O9 N2 | -0.04 | 441.1845(20) 279.1336(48) 261.1230(100) 155.0814(12) 127.0867(14) |  |  |  |  | - | + | - | - |
| M61 | 5.28 | 274.1186 | C14 H16 O3 N3 | -0.10 | 274.1182(25) 228.1130(32) 156.0766(70) 110.0716(100) |  |  |  |  | - | + | - | + |
| M62 | 5.32 | 537.1199 | C19 H25 O16 N2 | 0.02 | 375.0665(15) 329.0617(12) 254.0477(63) 238.0528(100) 210.0582(23) 176.0705(70) |  |  |  |  | + | + | + | + |
| M63 | 5.41 |  |  |  |  | 369.1303 | C16 H21 O8 N2 | -0.24 | 369.1300(3) 207.0769(29) 192.0882(100) 150.3210(1) 101.0338(1) | - | + | - | - |
| M64 | 5.41 |  |  |  |  | 194.0815 | C10 H12 O3 N | -3.74 | 194.0814(100) 146.0600(9) 116.0335(4) | - | + | - | + |
| M65 | 5.43 | 466.1820 | C21 H28 O9 N3 | -0.12 | 304.1289(100) 286.1181(52) 258.1238(32) 214.1336(17) 170.0923(67) 124.0870(59) |  |  |  |  | - | + | - | - |
| M66 | 5.56 | 227.1755 | C12 H23 O2 N2 | 0.20 | 227.1752(100) 209.1647(23) 114.0916(16) |  |  |  |  | - | + | - | - |
| M67 | 5.59 | 442.1822 | C19 H28 O9 N3 | 0.35 | 442.1806(46) 280.1290(100) 267.1699(17) 263.1026(28) 134.0602(10) |  |  |  |  | - | + | + | - |
| M68 | 5.63 |  |  |  |  | 361.1142 | C15 H21 O10 | 0.50 | 315.1095(10) 192.0879(5) 161.0443(25) 152.0466(100) 137.0229(25) 101.0230(18) | - | + | - | - |
| M69 | 5.74 | 239.2118 | C14 H27 O N2 | -0.03 | 239.2109(7) 221.2008(22) 192.0876(14) 157.0682(31) 127.1231(77) 109.0652(40) |  |  |  |  | - | + | - | - |
| M70 | 5.81 |  |  |  |  | 474.0738 | C14 H20 O17 N | 0.31 | 312.1092(30) 221.0666(7) 161.0444(64) 153.0547(34) 150.0550(91) 119.0335(48) 111.0075(90) | - | + | - | - |
| M71 | 5.81 | 288.1341 | C15 H18 O3 N3 | -0.15 | 288.1340(100) 244.1440(21) 170.0923(26) 153.0659(15) 124.0870(25) 109.0763(61) |  |  |  |  | - | + | - | - |
| M72 | 5.84 | 358.1497 | C16 H24 O8 N | 0.13 | 196.0965(27) 189.0868(48) 178.0861(74) 150.0912(100) | 356.1352 | C16 H22 O8 N | 0.20 | 356.1358(2) 194.0814(100) 187.0714(24) 113.0228(1) 101.0229(3) | + | + | + | - |
| M73 | 5.84 | 308.1766 | C19 H22 O N3 | 0.83 | 308.2062(70) 242.1131(18) 223.1237(28) 175.0864(100) 143.1179(81) 129.1023(57) |  |  |  |  | - | + | - | - |
| M74 | 5.90 |  |  |  |  | 515.2251 | C23 H35 O11 N2 | 0.91 | 158.0811(100) | - | + | - | - |
| M75 | 5.92 |  |  |  |  | 235.1086 | C12 H15 O3 N2 | -1.13 | 235.1077(7) 129.0656(100) 111.0550(64) 102.0546(25) | - | + | - | - |
| M76 | 5.93 | 385.1604 | C17 H25 O8 N2 | -0.45 | 385.1598(6) 223.1075(100) 159.0916(6) |  |  |  |  | + | + | + | + |
| M77 | 5.93 | 514.1318 | C23 H25 O11 N Na | -0.28 | 514.1315(100) |  |  |  |  | + | - | - | - |
| M78 | 5.98 | 476.1761 | C20 H30 O12 N | -0.40 | 152.0705(23) 134.0600(100) 106.0654(16) | 520.1669 | C21 H30 O14 N | -0.48 | 474.1621(46) 312.1090(54) 263.0769(19) 161.0444(75) 150.0548(73) 101.0230(100) | + | + | + | - |
| M79 | 5.99 | 314.1232 | C14 H20 O7 N | -0.63 | 134.0600(100) 106.0655(29) |  |  |  |  | - | + | - | - |
| M80 | 6.00 |  |  |  |  | 510.1387 | C10 H24 O15 N9 | -1.06 | 510.1376(26) 179.0552(19) 161.0444(33) 125.0233(20) 119.0336(33) 113.0230(35) | + | + | + | - |
| M81 | 6.00 | 511.1429 | C19 H23 O11 N6 | 0.95 | 256.0743(4) 190.0609(18) 112.0508(100) |  |  |  |  | - | + | - | - |
| M82 | 6.02 | 498.1581 | C18 H24 O10 N7 | 0.22 | 498.1576(100) |  |  |  |  | + | + | + | - |
| M83 | 6.07 |  |  |  |  | 510.1011 | C9 H20 O16 N9 | -2.21 | 101.0230(71) | + | - | + | - |
| M84 | 6.16 |  |  |  |  | 622.1368 | C22 H28 O18 N3 | -0.89 | 212.0016(100) | + | + | - | - |
| M85 | 6.16 |  |  |  |  | 267.0876 | C13 H15 O6 | 0.52 | 187.0062(14) 161.0444(100) 149.0598(3) 131.0337(67) | - | + | - | - |
| M86 | 6.17 | 261.2173 | C13 H29 O3 N2 | -0.02 | 261.1263(14) 198.1850(11) 130.0863(72) 114.1279(11) |  |  |  |  | - | + | - | - |
| M87 | 6.18 | 308.1766 | C19 H22 O N3 | 0.83 | 308.2060(57) 223.1242(46) 204.1338(19) 175.0860(29) 143.1177(100) |  |  |  |  | - | + | - | - |
| M88 | 6.34 |  |  |  |  | 204.0520 | C7 H10 O6 N | 3.28 | 204.0520(100) 161.0458(20) 159.0916(22) | - | + | - | - |
| M89 | 6.45 |  |  |  |  | 331.1035 | C14 H19 O9 | 0.20 | 161.0447(3) 113.0231(1) 101.0229(1) | - | + | - | - |
| M90 | 6.45 | 351.1802 | C19 H27 O6 | -0.04 | 335.1745(60) 321.1782(33) 306.6636(26) 116.0708(100) |  |  |  |  | - | + | - | - |
| M91 | 6.49 | 332.1339 | C14 H22 O8 N | -0.25 | 145.0495(35) 135.0441(40) 127.0391(25) | 313.0931 | C14 H17 O8 | 0.51 | 313.0930(9) 161.0445(28) 161.0442(27) 159.0284(9) 113.0230(50) 101.0230(100) | - | + | + | + |
| M92 | 6.56 | 329.1344 | C14 H21 O7 N2 | 0.13 | 196.0827(15) 145.0495(17) 132.0444(84) 127.0387(5) |  |  |  |  | - | + | - | - |
| M93 | 6.57 |  |  |  |  | 523.1016 | C13 H23 O18 N4 | 0.64 | 212.0017(100) 132.0439(1) | - | + | - | - |
| M94 | 6.57 | 485.2362 | C24 H37 O10 | -1.94 | 459.2191(5) 414.1975(9) 244.1292(19) 129.1024(22) |  |  |  |  | - | + | - | - |
| M95 | 6.58 |  |  |  |  | 488.1409 | C20 H26 O13 N | -0.13 | 488.1404(100) 312.1088(14) 161.0447(19) 150.0547(70) 113.0230(91) 101.0229(19) | + | + | + | + |
| M96 | 6.76 |  |  |  |  | 356.1350 | C16 H22 O8 N | -0.14 | 356.1343(2) 150.0553(1) 113.0229(1) 101.0228(5) | + | + | - | - |
| M97 | 6.78 | 537.1199 | C19 H25 O16 N2 | 0.02 | 375.0682(20) 329.0620(11) 254.0477(52) 238.0529(100) 210.0580(23) 120.0116(24) |  |  |  |  | + | + | - | - |
| M98 | 6.78 | 351.1550 | C17 H23 O6 N2 | -0.03 | 351.1546(4) 189.1020(30) 171.0915(100) |  |  |  |  | - | + | - | - |
| M99 | 6.79 | 471.2087 | C19 H35 O13 | 1.52 | 471.2080(71) 309.1553(100) 274.1183(36) 246.1235(76) 175.1189(18) |  |  |  |  | - | + | - | - |
| M100 | 6.85 | 561.2291 | C24 H37 O13 N2 | 0.20 | 561.2286(30) 399.1758(10) 237.1230(100) | 559.2142 | C24 H35 O13 N2 | -0.42 | 559.2148(100) 397.1633(8) 235.1083(12) 129.0659(23) 102.0545(18) | - | + | - | - |
| M101 | 6.85 | 623.3262 | C29 H51 O14 | -1.77 | 623.3255(100) 606.2976(6) 578.3061(8) |  |  |  |  | - | + | - | - |
| M102 | 6.90 | 332.1339 | C14 H22 O8 N | -0.25 | 145.0494(43) 135.0440(45) 127.0390(30) | 313.0930 | C14 H17 O8 | 0.32 | 313.0933(6) 161.0444(31) 113.0231(56) 101.0230(100) | - | + | + | + |
| M103 | 6.96 | 329.1344 | C14 H21 O7 N2 | 0.13 | 239.1509(4) 145.0494(20) 132.0444(100) 127.0388(9) |  |  |  |  | - | + | - | - |
| M104 | 6.97 | 288.1341 | C15 H18 O3 N3 | -0.15 | 288.1345(25) 242.1281(24) 199.1077(15) 170.0923(48) 124.0870(71) |  |  |  |  | - | + | - | - |
| M105 | 6.97 | 594.1426 | C21 H28 O17 N3 | 1.26 | 386.0824(12) 265.0644(14) 254.0482(48) 233.0916(34) 177.0327(100) |  |  |  |  | - | + | - | - |
| M106 | 6.98 |  |  |  |  | 523.1016 | C13 H23 O18 N4 | 0.64 | 427.0637(1) 212.0016(100) 132.0439(1) | - | + | - | - |
| M107 | 7.03 | 493.1310 | C19 H21 O10 N6 | -0.36 | 331.0778(24) 210.0579(59) 194.0632(100) 176.0703(35) 164.0525(13) 120.0118(12) | 491.1160 | C18 H23 O14 N2 | 1.01 | 404.0848(63) 372.1125(23) 370.0964(92) 312.1090(14) 210.0585(40) 176.0709(35) 150.0548(87) | - | + | - | - |
| M108 | 7.07 |  |  |  |  | 285.0982 | C13 H17 O7 | 0.72 | 267.1004(2) 206.0458(10) 179.0553(8) 119.0336(31) 113.0231(22) 101.0227(22) | - | + | - | - |
| M109 | 7.09 | 399.1761 | C18 H27 O8 N2 | -0.10 | 399.1758(20) 237.1230(100) | 397.1617 | C18 H25 O8 N2 | 0.03 | 397.1610(100) 235.1078(22) 161.0444(19) 129.0654(46) 102.0546(53) | - | + | - | - |
| M110 | 7.14 | 291.1456 | C13 H23 O7 | 1.77 | 291.1449(100) 273.1292(7) 247.1562(8) 120.0808(12) 112.0873(30) |  |  |  |  | - | + | - | + |
| M111 | 7.23 | 248.1858 | C12 H26 O4 N | 0.12 | 248.1139(100) 230.1034(12) |  |  |  |  | - | + | - | - |
| M112 | 7.26 | 322.1977 | C18 H28 O4 N | -3.57 | 322.1758(100) 199.1073(18) 172.0968(22) 130.0863(13) 112.0760(54) |  |  |  |  | - | + | - | - |
| M113 | 7.28 |  |  |  |  | 395.1194 | C15 H23 O12 | -0.20 | 357.0617(57) 269.1253(56) 267.1102(100) 130.1727(58) 113.0232(88) 101.0231(81) | - | + | - | - |
| M114 | 7.32 |  |  |  |  | 267.0876 | C13 H15 O6 | 0.52 | 223.0955(3) 161.0446(100) 131.0337(54) 125.0239(4) | - | + | - | - |
| M115 | 7.37 | 322.1762 | C16 H24 O4 N3 | 0.15 | 322.1756(99) 280.1673(7) 130.0864(22) 112.0759(100) |  |  |  |  | + | + | + | + |
| M116 | 7.37 | 297.1401 | C14 H21 O5 N2 | -4.45 | 136.0617(100) |  |  |  |  | - | + | - | - |
| M117 | 7.37 | 367.1862 | C18 H27 O6 N2 | -0.17 | 367.1858(100) 205.1333(96) |  |  |  |  | - | + | - | - |
| M118 | 7.38 |  |  |  |  | 533.1712 | C19 H33 O17 | -2.11 | 212.0016(100) 132.0441(2) | - | + | - | - |
| M119 | 7.42 | 411.1765 | C19 H27 O8 N2 | 0.70 | 411.1757(36) 249.1230(100) 161.0743(16) |  |  |  |  | - | + | - | - |
| M120 | 7.44 |  |  |  |  | 320.1613 | C16 H22 O4 N3 | -1.03 | 320.1615(11) 214.1190(100) 172.1080(64) | + | + | - | + |
| M121 | 7.47 | 273.1339 | C13 H21 O6 | 2.47 | 273.1346(5) 138.0661(100) 114.0552(15) |  |  |  |  | + | + | - | + |
| M122 | 7.48 | 351.1550 | C17 H23 O6 N2 | -0.03 | 351.1543(5) 189.1020(38) 171.0915(100) |  |  |  |  | - | + | - | - |
| M123 | 7.55 | 291.1456 | C13 H23 O7 | 1.77 | 291.1450(100) 247.1554(18) 173.1032(27) 129.0548(19) 112.0872(60) |  |  |  |  | - | + | - | + |
| M124 | 7.72 |  |  |  |  | 230.1393 | C11 H20 O4 N | -2.22 | 230.1391(21) 182.1179(3) 151.0388(100) 141.0905(3) | - | + | - | - |
| M125 | 7.74 | 205.1337 | C12 H17 O N2 | 0.19 | 205.1335(90) 187.1230(17) 178.0497(68) 120.0810(38) |  |  |  |  | - | + | - | - |
| M126 | 7.85 |  |  |  |  | 308.0776 | C14 H14 O7 N | 0.01 | 262.0069(15) 113.0232(100) | + | - | - | - |
| M127 | 7.89 | 294.1451 | C14 H20 O4 N3 | 0.94 | 294.1788(8) 120.0810(7) |  |  |  |  | - | - | - | + |
| M128 | 7.96 | 407.2046 | C22 H31 O7 | -1.81 | 310.1468(2) 235.1188(100) 207.1241(24) 166.0610(73) 162.1020(11) 110.0716(29) |  |  |  |  | - | + | - | - |
| M129 | 7.98 |  |  |  |  | 475.1460 | C20 H27 O13 | 0.60 | 261.0616(63) 212.0022(48) 112.0720(36) | + | + | + | - |
| M130 | 7.99 | 251.1389 | C13 H19 O3 N2 | -0.10 | 251.1387(100) 233.1284(9) 133.0760(6) |  |  |  |  | + | + | - | + |
| M131 | 8.01 | 241.2274 | C14 H29 O N2 | -0.08 | 241.2274(10) 127.1230(60) |  |  |  |  | - | + | - | - |
| M132 | 8.03 |  |  |  |  | 285.0981 | C13 H17 O7 | 0.26 | 285.0986(22) 206.0446(2) 167.0564(15) 113.0231(22) 101.0227(22) | - | + | - | - |
| M133 | 8.07 | 293.1608 | C13 H25 O7 | 1.32 | 293.1601(100) 276.1343(30) 234.1121(55) 233.1279(23) 175.1189(26) 158.0923(42) |  |  |  |  | - | + | - | - |
| M134 | 8.07 | 367.1862 | C18 H27 O6 N2 | -0.17 | 367.1856(72) 205.1334(100) |  |  |  |  | - | + | - | - |
| M135 | 8.09 | 323.1102 | C14 H20 O7 Na | 0.24 | 323.1100(96) 199.1077(18) 172.0967(23) 130.0864(18) 112.0760(68) | 345.1189 | C15 H21 O9 | -0.51 | 310.0935(27) 299.1143(12) 161.0442(100) 159.0286(9) 148.0394(54) 121.0283(54) 113.0230(57) 101.0230(87) | + | + | - | - |
| M136 | 8.11 | 484.2268 | C22 H34 O9 N3 | -2.21 | 484.2279(58) 361.1597(4) 322.1757(100) 286.1395(4) 199.1074(15) 112.0760(27) |  |  |  |  | - | + | - | - |
| M137 | 8.12 |  |  |  |  | 444.1516 | C19 H26 O11 N | 1.07 | 150.0549(15) 101.0229(10) | - | + | - | - |
| M138 | 8.12 |  |  |  |  | 433.1351 | C18 H25 O12 | -0.05 | 295.0834(5) 235.0460(5) 161.0603(27) 151.0387(100) 133.0645(10) | - | + | - | - |
| M139 | 8.17 | 517.2154 | C22 H29 O7 N8 | 0.02 | 347.1548(1) 329.1420(1) 217.0969(64) 144.0806(100) |  |  |  |  | - | + | - | - |
| M140 | 8.21 | 242.1170 | C15 H16 O2 N | -0.56 | 242.1170(66) 225.0907(31) 207.0803(11) 151.0629(12) |  |  |  |  | - | + | - | - |
| M141 | 8.22 | 567.2543 | C27 H39 O11 N2 | -0.89 | 456.2078(2) 286.1394(6) 217.0969(100) 144.0807(97) |  |  |  |  | - | + | - | - |
| M142 | 8.25 | 433.1866 | C23 H29 O8 | 1.98 | 420.2091(3) 217.0969(81) 144.0806(100) |  |  |  |  | - | + | - | - |
| M143 | 8.26 |  |  |  |  | 310.0931 | C14 H16 O7 N | -0.57 | 161.0444(5) 148.0391(43) 121.0281(100) 113.0228(3) 101.0230(5) | + | + | - | - |
| M144 | 8.26 |  |  |  |  | 356.0987 | C15 H18 O9 N | -0.13 | 310.0932(24) 161.0444(12) 148.0391(83) 121.0281(100) | - | + | - | - |
| M145 | 8.26 |  |  |  |  | 431.1724 | C23 H27 O8 | 2.83 | 215.0820(100) 171.0917(25) 142.0649(5) 116.0492(14) | - | + | - | - |
| M146 | 8.26 |  |  |  |  | 523.1016 | C13 H23 O18 N4 | 0.64 | 215.0812(1) 212.0926(2) 178.0497(4) 161.0444(2) 132.0440(2) | - | + | - | - |
| M147 | 8.28 | 217.0972 | C12 H13 O2 N2 | 0.35 | 217.1047(23) 144.0808(100) | 215.0821 | C12 H11 O2 N2 | -2.38 | 215.0822(51) 171.0917(91) 142.0649(21) 116.0492(61) | + | + | + | - |
| M148 | 8.28 | 496.1493 | C15 H30 O17 N | -3.09 | 496.1490(60) 409.1167(6) 362.0915(10) 281.0619(10) 241.0716(100) 217.0971(15) |  |  |  |  | - | + | - | - |
| M149 | 8.33 |  |  |  |  | 515.2035 | C26 H31 O9 N2 | -0.03 | 515.1238(1) 469.1203(5) 299.0353(3) 215.0819(100) 171.0917(15) | - | + | - | - |
| M150 | 8.33 | 398.2268 | C19 H32 O6 N3 | -1.78 | 398.2259(26) 384.2313(11) 217.0971(13) 183.1126(17) 143.1179(20) 129.1023(100) |  |  |  |  | - | + | - | - |
| M151 | 8.33 | 517.2154 | C22 H29 O7 N8 | 0.02 | 217.0970(56) 163.0422(6) 144.0807(100) |  |  |  |  | - | + | - | - |
| M152 | 8.35 |  |  |  |  | 345.1189 | C15 H21 O9 | -0.51 | 310.0936(45) 299.1139(33) 161.0444(89) 148.0392(80) 121.0281(100) 113.0230(63) 101.0229(81) | - | + | - | - |
| M153 | 8.41 | 594.1426 | C21 H28 O17 N3 | 1.26 | 432.0888(14) 386.0825(11) 265.0641(15) 254.0477(47) 233.0918(35) 177.0326(100) |  |  |  |  | - | + | - | - |
| M154 | 8.41 | 598.2826 | C24 H44 O14 N3 | 1.36 | 598.2831(12) 383.1931(55) 286.1392(50) 201.0869(31) 173.0920(67) |  |  |  |  | - | + | - | - |
| M155 | 8.44 | 577.2384 | C28 H37 O11 N2 | -1.36 | 577.2200(9) 217.0970(89) 144.0807(100) | 575.2248 | C28 H35 O11 N2 | 0.27 | 359.1348(2) 215.0819(100) 197.0808(3) 171.0915(20) 116.0490(12) | - | + | - | - |
| M156 | 8.52 | 323.2327 | C18 H31 O3 N2 | -0.59 | 323.2316(1) 127.1231(61) |  |  |  |  | - | + | - | - |
| M157 | 8.56 |  |  |  |  | 576.1932 | C24 H34 O15 N | -0.37 | 576.1943(20) 426.1413(4) 312.1101(15) 294.0973(22) 192.0655(22) 150.0548(55) 131.0332(35) 113.0229(84) | - | + | - | - |
| M158 | 8.61 | 241.2274 | C14 H29 O N2 | -0.08 | 241.2272(21) 223.2166(13) 127.1231(88) |  |  |  |  | - | + | - | - |
| M159 | 8.62 |  |  |  |  | 525.1615 | C24 H29 O13 | 0.26 | 301.1082(56) 175.0238(14) 117.0178(14) 113.0230(100) | - | + | - | - |
| M160 | 8.64 | 316.1657 | C17 H22 O3 N3 | 0.45 | 316.1653(100) 299.1018(7) 145.0759(21) |  |  |  |  | - | + | - | - |
| M161 | 8.64 | 377.1820 | C17 H29 O9 | 3.56 | 377.1815(2) 359.1715(3) 271.1397(100) 229.1294(7) 166.0974(20) 158.0811(18) |  |  |  |  | - | + | - | - |
| M162 | 8.67 |  |  |  |  | 345.1189 | C15 H21 O9 | -0.51 | 299.1163(7) 161.0444(9) 137.0595(100) 119.0336(10) 101.0228(15) | - | + | - | - |
| M163 | 8.70 |  |  |  |  | 374.0551 | C14 H16 O9 N S | -0.12 | 374.0549(100) 161.0436(1) 113.0229(5) 101.0227(1) | + | + | + | - |
| M164 | 8.78 | 249.1235 | C13 H17 O3 N2 | 0.53 | 249.1231(100) 231.1128(8) 204.1016(6) 185.1073(13) |  |  |  |  | + | + | - | + |
| M165 | 8.78 | 473.2541 | C27 H37 O7 | 1.44 | 372.1863(8) 201.1230(9) 173.1284(100) 133.0608(22) |  |  |  |  | - | + | - | - |
| M166 | 8.85 | 536.1976 | C22 H34 O14 N | 0.35 | 195.0651(52) 177.0545(100) 145.0495(23) 127.0391(15) | 517.1562 | C22 H29 O14 | -0.17 | 193.0498(100) 178.0261(13) 134.0361(7) | + | + | + | - |
| M167 | 8.86 | 377.1820 | C17 H29 O9 | 3.56 | 377.1822(3) 271.1398(100) 229.1292(4) 166.0974(12) 158.0811(11) |  |  |  |  | - | + | - | - |
| M168 | 8.89 | 306.1814 | C16 H24 O3 N3 | 0.59 | 306.1806(51) 200.1027(100) 172.0966(24) 130.0863(14) |  |  |  |  | - | + | - | - |
| M169 | 9.05 | 416.2323 | C26 H30 O2 N3 | -2.29 | 372.1873(11) 243.1455(10) 173.1283(100) 147.0761(20) 130.0499(19) |  |  |  |  | - | + | - | - |
| M170 | 9.13 | 453.2626 | C28 H37 O5 | -2.03 | 277.1536(1) 192.1051(21) 175.0786(100) 115.0756(11) |  |  |  |  | - | + | - | - |
| M171 | 9.19 | 355.1020 | C16 H19 O9 | -0.95 | 193.0495(100) |  |  |  |  | - | + | - | - |
| M172 | 9.19 | 571.2911 | C32 H43 O9 | 1.72 | 380.1922(100) 352.1975(11) 175.0786(26) |  |  |  |  | - | + | - | - |
| M173 | 9.36 | 450.1970 | C19 H32 O11 N | 0.01 | 254.1386(8) 145.0494(42) 127.0391(24) | 477.1614 | C20 H29 O13 | 0.10 | 431.1558(55) 269.1034(66) 161.0445(75) 113.0230(45) 101.0230(100) | + | + | - | - |
| M174 | 9.36 | 230.0923 | C12 H12 O2 N3 | -0.49 | 230.0922(100) 144.0807(2) |  |  |  |  | - | + | - | - |
| M175 | 9.38 | 637.2448 | C26 H41 O16 N2 | -0.39 | 163.0599(20) 145.0495(81) 127.0391(41) | 664.2098 | C27 H38 O18 N | 0.61 | 618.2081(5) 407.1202(9) 383.1192(9) 221.0663(42) 179.0552(100) 161.0446(32) | - | + | - | - |
| M176 | 9.39 | 563.2098 | C23 H35 O14 N2 | 2.77 | 293.0900(100) |  |  |  |  | + | + | - | - |
| M177 | 9.39 | 271.1176 | C13 H19 O6 | -0.13 | 145.0494(5) 127.0390(5) | 269.1030 | C13 H17 O6 | -0.34 | 161.0446(17) 113.0230(49) 101.0229(100) | + | + | - | - |
| M178 | 9.39 | 425.1459 | C20 H25 O10 | 3.87 | 369.0852(30) 327.0747(49) 309.0638(11) 263.1388(10) |  |  |  |  | - | + | - | - |
| M179 | 9.41 | 477.1867 | C23 H29 O9 N2 | -0.04 | 477.1860(6) 315.1334(100) 165.0546(7) |  |  |  |  | + | + | - | - |
| M180 | 9.45 |  |  |  |  | 315.1086 | C14 H19 O8 | 0.12 | 101.0225(100) | + | + | + | - |
| M181 | 9.45 |  |  |  |  | 359.1346 | C16 H23 O9 | -0.32 | 359.1091(6) 313.1298(21) 279.0873(22) 161.0447(73) 113.0230(44) 101.0230(66) | - | + | + | - |
| M182 | 9.49 |  |  |  |  | 263.1401 | C14 H19 O3 N2 | -0.25 | 264.1412(9) 219.1501(10) 164.0706(13) 115.0863(62) | - | + | - | - |
| M183 | 9.55 |  |  |  |  | 473.1235 | C19 H25 O10 N2 S | 0.00 | 473.1222(8) 101.0229(3) | - | + | - | - |
| M184 | 9.57 | 378.1164 | C16 H21 O8 N Na | 0.42 | 174.0542(2) | 400.1250 | C17 H22 O10 N | 0.20 | 113.0230(10) 101.0228(7) | - | + | - | - |
| M185 | 9.57 | 425.1918 | C20 H29 O8 N2 | -0.03 | 425.1914(4) 369.0852(30) 309.0638(11) |  |  |  |  | - | + | - | - |
| M186 | 9.70 |  |  |  |  | 416.1022 | C9 H18 O12 N7 | 0.74 | 416.1003(7) 382.1133(1) 338.1242(20) 176.0706(100) 120.0111(4) | - | + | + | - |
| M187 | 9.78 | 355.1020 | C16 H19 O9 | -0.95 | 327.0745(4) 193.0493(11) 127.1230(80) |  |  |  |  | - | + | - | - |
| M188 | 9.86 | 295.1775 | C14 H23 O3 N4 | 3.57 | 130.0652(18) |  |  |  |  | - | + | - | - |
| M189 | 9.89 | 216.1021 | C13 H14 O2 N | 0.95 | 216.1020(29) 198.0913(100) 170.0964(11) 144.0807(10) |  |  |  |  | - | + | - | - |
| M190 | 9.91 | 321.1806 | C17 H25 O4 N2 | -0.79 | 321.1806(100) 303.1701(27) 261.1594(41) 216.1382(33) 198.1276(47) |  |  |  |  | - | + | - | - |
| M191 | 9.92 |  |  |  |  | 492.1282 | C10 H22 O14 N9 | -2.03 | 323.0986(65) 221.0654(30) 179.0552(21) 161.0450(24) 125.0230(27) 119.0336(49) 113.0231(30) | + | + | + | - |
| M192 | 9.93 |  |  |  |  | 519.1467 | C20 H27 O14 N2 | -0.21 | 471.0001(3) 182.1231(1) | + | + | + | - |
| M193 | 9.97 | 352.2027 | C21 H26 O2 N3 | 2.20 | 253.1180(76) 207.1126(11) 187.1076(36) 166.0862(57) 159.1127(45) |  |  |  |  | - | + | - | - |
| M194 | 9.98 | 477.1970 | C21 H33 O12 | 0.77 | 145.0494(62) 127.0390(33) |  |  |  |  | + | + | - | - |
| M195 | 9.98 |  |  |  |  | 644.1654 | C19 H30 O18 N7 | 0.20 | 187.0061(100) 107.0487(8) | - | + | - | - |
| M196 | 10.00 | 197.0809 | C10 H13 O4 | 0.18 | 197.0809(11) 179.0702(35) 151.0389(100) 125.0599(26) |  |  |  |  | - | + | - | - |
| M197 | 10.01 | 475.1921 | C20 H31 O11 N2 | -0.39 | 296.1124(2) 163.0599(12) 145.0495(64) 127.0391(33) | 502.1565 | C21 H28 O13 N | -0.31 | 456.1511(2) 323.0984(33) 179.0552(26) 161.0445(33) 125.0231(26) 119.0336(63) | + | + | + | - |
| M198 | 10.01 | 337.2155 | C23 H29 O2 | -2.04 | 261.1556(14) 173.1285(12) 162.0548(100) 129.1022(11) 113.0236(11) |  |  |  |  | - | + | - | - |
| M199 | 10.03 | 303.1335 | C16 H19 O4 N2 | -0.39 | 303.1335(41) 285.1233(11) 256.0966(11) 152.0704(100) 134.0599(14) |  |  |  |  | + | + | - | + |
| M200 | 10.07 | 165.0911 | C10 H13 O2 | 0.51 | 124.0757(23) 120.0809(45) 107.0494(19) |  |  |  |  | - | + | - | - |
| M201 | 10.18 | 425.1918 | C20 H29 O8 N2 | -0.03 | 425.1915(44) 263.1387(100) 173.0921(3) |  |  |  |  | - | + | - | - |
| M202 | 10.20 | 512.2646 | C29 H38 O7 N | 0.61 | 427.2115(47) 342.1764(71) 171.0762(51) 155.0811(17) 143.1179(60) |  |  |  |  | - | + | - | - |
| M203 | 10.22 |  |  |  |  | 328.1041 | C14 H18 O8 N | 1.04 | 161.0444(13) 113.0227(11) 101.0228(14) | - | + | - | - |
| M204 | 10.25 |  |  |  |  | 477.1614 | C20 H29 O13 | 0.16 | 431.1566(73) 269.1041(34) 179.0548(24) 161.0445(49) 113.0230(68) 101.0227(68) | - | + | - | - |
| M205 | 10.33 | 230.1176 | C14 H16 O2 N | 0.06 | 230.1173(100) 186.0911(17) |  |  |  |  | + | + | - | + |
| M206 | 10.52 | 413.1921 | C19 H29 O8 N2 | 0.58 | 413.1914(12) 251.1387(100) 187.1230(6) | 411.1773 | C19 H27 O8 N2 | 0.05 | 411.1767(56) 393.1671(16) 331.1187(17) 231.1137(18) 213.1027(87) 202.1160(21) 119.0336(56) | + | + | - | + |
| M207 | 10.52 |  |  |  |  | 373.1141 | C16 H21 O10 | 0.13 | 267.0879(1) 237.0757(6) 207.0655(46) 179.0704(24) 165.0545(100) 119.0488(17) 113.0230(19) | - | + | - | - |
| M208 | 10.54 | 281.1133 | C13 H17 O5 N2 | 0.19 | 281.1137(21) 263.1031(39) 236.0914(100) 208.0967(69) 162.0911(45) |  |  |  |  | - | + | - | - |
| M209 | 10.60 |  |  |  |  | 618.2044 | C26 H36 O16 N | 0.64 | 618.2037(2) 323.0995(15) 179.0550(17) 161.0445(33) 113.0230(100) | - | + | - | - |
| M210 | 10.68 | 587.2446 | C26 H39 O13 N2 | -0.06 | 587.2429(9) 425.1913(95) 263.1387(100) |  |  |  |  | + | + | - | - |
| M211 | 10.68 |  |  |  |  | 463.1830 | C20 H31 O12 | 1.97 | 417.1783(9) 343.1397(100) 181.0860(14) | + | + | - | - |
| M212 | 10.74 | 151.0753 | C9 H11 O2 | -0.64 | 151.0753(100) |  |  |  |  | - | + | - | - |
| M213 | 10.75 |  |  |  |  | 632.1830 | C26 H34 O17 N | -0.38 | 632.1832(100) 323.0987(4) 161.0443(4) 125.0231(7) 119.0337(9) | - | + | - | - |
| M214 | 10.84 |  |  |  |  | 445.1353 | C19 H25 O12 | 0.34 | 445.1353(100) 269.1031(16) 161.0445(17) 113.0230(89) 101.0229(31) | + | + | - | - |
| M215 | 10.85 | 313.1395 | C14 H21 O6 N2 | 0.28 | 145.0496(39) 127.0393(27) | 340.1037 | C15 H18 O8 N | -0.18 | 188.0560(5) 161.0445(100) 159.0287(6) 113.0230(21) 101.0231(23) | + | + | + | - |
| M216 | 10.85 |  |  |  |  | 465.1877 | C22 H29 O9 N2 | -0.31 | 170.0812(100) 126.0911(14) | - | + | - | - |
| M217 | 10.86 |  |  |  |  | 308.0776 | C14 H14 O7 N | 0.02 | 187.0064(100) 161.0601(31) 153.0567(21) 131.0363(39) 107.0495(26) | + | + | - | + |
| M218 | 10.87 | 610.1912 | C15 H32 O17 N9 | 0.25 | 352.0698(97) 325.0592(20) 270.1444(15) 191.0387(37) 116.0497(68) |  |  |  |  | - | + | - | - |
| M219 | 10.94 |  |  |  |  | 439.0204 | C10 H15 O19 | 3.50 | 394.0224(46) 350.0337(62) 158.9373(100) 123.9453(12) 114.9471(22) | + | + | + | - |
| M220 | 10.94 |  |  |  |  | 642.1138 | C18 H24 O19 N7 | 0.82 | 642.1154(100) 509.0628(25) 407.0311(36) 383.0313(10) | + | + | - | - |
| M221 | 10.97 |  |  |  |  | 384.1304 | C17 H22 O9 N | 0.95 | 384.1300(2) 305.1040(1) 181.0496(3) 176.0707(4) | + | + | + | - |
| M222 | 10.98 | 278.1023 | C14 H16 O5 N | -0.14 | 278.1020(46) 127.0394(16) |  |  |  |  | - | + | - | - |
| M223 | 10.98 |  |  |  |  | 555.1716 | C25 H31 O14 | -0.65 | 331.1184(13) 261.1242(100) 195.0653(10) 165.0545(16) 113.0230(55) | - | + | - | - |
| M224 | 10.99 | 413.1921 | C19 H29 O8 N2 | 0.58 | 413.1914(10) 251.1387(100) 187.1229(6) |  |  |  |  | + | + | - | - |
| M225 | 11.07 |  |  |  |  | 206.0817 | C11 H12 O3 N | -2.85 | 206.0812(58) 164.0707(38) | - | - | - | + |
| M226 | 11.12 | 195.1017 | C11 H15 O3 | 0.41 | 195.1014(92) 167.0700(66) 163.0751(78) 135.0803(41) 107.0494(100) |  |  |  |  | - | + | - | - |
| M227 | 11.17 |  |  |  |  | 419.1564 | C18 H27 O11 | 5.50 | 419.1904(33) 278.0935(57) 211.0975(57) 183.0763(21) | - | + | - | - |
| M228 | 11.17 | 327.1187 | C14 H19 O7 N2 | 0.10 | 145.0495(3) 127.0392(2) | 308.0777 | C14 H14 O7 N | 0.31 | 308.0777(71) 161.0598(25) 113.0230(69) | + | + | + | + |
| M229 | 11.23 |  | C18 H29 O8 | 3.28 | 373.1865(70) 331.1759(24) 268.1440(100) 175.0867(24) |  |  |  |  | - | + | - | - |
| M230 | 11.23 | 373.1869 | C13 H23 O7 | 1.77 | 291.1428(9) 273.1263(51) 179.0484(29) 162.0218(61) 113.0964(30) 112.0872(47) |  |  |  |  | - | + | - | - |
| M231 | 11.25 | 619.1802 | C25 H35 O14 N2 S | -0.18 | 163.0594(1) 145.0495(9) 127.0390(8) | 617.1663 | C25 H33 O14 N2 S | 0.75 | 617.1653(4) 179.0557(7) 161.0450(4) 119.0337(9) | + | + | - | - |
| M232 | 11.31 |  |  |  |  | 447.1513 | C19 H27 O12 | 1.14 | 401.1462(12) 269.1028(100) 161.0445(44) 101.0232(25) | - | + | - | - |
| M233 | 11.39 |  |  |  |  | 431.1560 | C19 H27 O11 | 6.50 | 432.1557(60) 195.0654(100) | + | + | - | - |
| M234 | 11.42 | 305.1497 | C16 H21 O4 N2 | 0.25 | 305.1498(100) 200.1070(38) 172.0964(24) 130.0863(45) 126.0914(34) |  |  |  |  | - | + | - | - |
| M235 | 11.42 | 323.1602 | C16 H23 O5 N2 | 0.22 | 305.1491(99) 277.1542(70) 263.1381(14) 218.1175(21) 200.1068(60) 172.0968(36) |  |  |  |  | + | + | + | - |
| M236 | 11.47 |  |  |  |  | 359.1346 | C16 H23 O9 | -0.32 | 313.1308(22) 161.0447(87) 124.0063(58) 113.0231(54) 101.0230(100) | - | + | - | - |
| M237 | 11.55 | 315.0697 | C13 H15 O9 | -4.22 | 145.0495(24) 127.0392(24) |  |  |  |  | - | + | - | - |
| M238 | 11.57 | 251.1389 | C13 H19 O3 N2 | -0.10 | 251.1387(100) 233.1285(10) 205.1336(18) 187.1229(37) |  |  |  |  | + | + | - | + |
| M239 | 11.57 |  |  |  |  | 525.1615 | C24 H29 O13 | 0.26 | 327.0869(10) 177.0548(6) 167.0338(100) 152.0103(16) 123.0437(9) | - | + | - | - |
| M240 | 11.57 | 347.1124 | C18 H19 O7 | -0.46 | 329.1527(35) 299.0912(30) 193.0492(23) 184.0967(49) 149.0595(31) |  |  |  |  | - | + | - | - |
| M241 | 11.64 |  |  |  |  | 336.0728 | C15 H14 O8 N | 0.78 | 161.0431(1) 113.0230(53) 101.0230(12) | - | + | - | - |
| M242 | 11.68 | 523.2897 | C28 H43 O9 | -0.82 | 485.2731(10) 372.1863(10) 244.0921(12) 226.1185(14) 187.1077(22) 173.1285(27) |  |  |  |  | - | + | - | - |
| M243 | 11.83 |  |  |  |  | 579.1935 | C24 H35 O16 | 0.83 | 533.1879(43) 149.0447(26) 131.0338(19) 113.0231(62) 107.0487(14) | - | + | - | - |
| M244 | 11.86 |  |  |  |  | 472.1463 | C20 H26 O12 N | 0.53 | 161.0443(53) 125.0232(20) | - | + | - | - |
| M245 | 11.88 | 408.1877 | C18 H26 O6 N5 | -0.20 | 408.1847(21) 362.1803(5) 295.1032(26) 277.0927(90) 249.0979(100) |  |  |  |  | - | + | - | - |
| M246 | 11.89 | 216.1021 | C13 H14 O2 N | 0.95 | 216.1021(11) 198.0915(50) 152.1069(28) 123.0806(26) |  |  |  |  | - | + | - | - |
| M247 | 11.93 |  |  |  |  | 521.1664 | C25 H29 O12 | -0.13 | 327.1238(16) 315.1238(69) 300.1013(8) 227.1278(4) 175.0234(16) 113.0229(100) | - | + | - | - |
| M248 | 11.96 |  |  |  |  | 634.1998 | C26 H36 O17 N | 3.15 | 377.1102(8) 191.0553(43) 149.0444(100) 131.0338(25) | - | + | - | - |
| M249 | 11.97 |  |  |  |  | 359.1346 | C16 H23 O9 | -0.32 | 313.1291(50) 269.1020(4) 161.0445(96) 119.0335(30) 101.0229(100) | - | + | - | - |
| M250 | 11.98 |  |  |  |  | 499.1473 | C22 H27 O13 | 3.14 | 204.0693(100) 126.0911(70) | - | + | - | - |
| M251 | 12.14 | 472.1460 | C20 H26 O12 N | 2.22 | 310.0916(67) 296.1121(20) 278.1013(16) 250.1071(16) 212.0703(24) 162.0760(51) |  |  |  |  | + | + | - | - |
| M252 | 12.15 | 303.0706 | C12 H15 O9 | -1.65 | 145.0490(14) 135.0803(12) 127.0390(29) |  |  |  |  | - | + | - | - |
| M253 | 12.19 | 645.2494 | C28 H41 O15 N2 | -1.17 | 174.1123(36) 141.0181(9) |  |  |  |  | - | + | - | - |
| M254 | 12.21 | 489.1711 | C20 H29 O12 N2 | -0.92 | 163.0599(18) 159.0287(17) 145.0494(74) 141.0181(100) 127.0390(33) | 470.1299 | C20 H24 O12 N | -1.04 | 470.1304(89) 188.0553(5) 161.0443(46) 159.0291(4) 113.0230(100) 101.0229(13) | + | + | + | + |
| M255 | 12.21 |  |  |  |  | 447.1514 | C19 H27 O12 | 1.28 | 401.1451(67) 149.0448(13) 131.0336(17) | - | + | - | - |
| M256 | 12.24 |  |  |  |  | 461.1671 | C20 H29 O12 | 1.37 | 415.1605(55) 269.1033(100) 161.0443(74) 101.0230(72) | - | + | - | - |
| M257 | 12.27 |  |  |  |  | 405.1400 | C17 H25 O11 | -0.63 | 124.0061(100) | - | + | - | - |
| M258 | 12.28 |  |  |  |  | 373.1141 | C16 H21 O10 | 0.13 | 310.0817(2) 241.0025(12) | + | + | - | - |
| M259 | 12.29 | 179.0701 | C10 H11 O3 | -0.73 | 179.0688(39) 137.0596(100) |  |  |  |  | - | + | - | - |
| M260 | 12.30 | 251.1389 | C13 H19 O3 N2 | -0.10 | 251.1386(100) 233.1283(11) 205.1333(18) 187.1228(38) 118.0862(10) |  |  |  |  | - | + | - | + |
| M261 | 12.35 | 271.1194 | C13 H19 O6 | 1.81 | 253.0963(3) 179.0700(12) 137.0596(100) | 269.1044 | C13 H17 O6 | 4.97 | 269.1033(14) 195.0654(100) 151.0753(14) 136.0517(13) | - | + | + | + |
| M262 | 12.36 | 457.1268 | C19 H25 O9 N2 S | -1.59 | 145.0494(1) 127.0388(2) | 455.1134 | C19 H23 O9 N2 S | 0.87 | 275.0497(100) 197.0712(20) 174.0372(25) 161.0450(1) 113.0231(8) 101.0230(12) | + | + | + | + |
| M263 | 12.48 | 545.3288 | C24 H45 O8 N6 | -0.99 | 300.1913(29) 282.1810(49) 246.1443(15) 213.1596(53) 201.1232(38) 185.1647(64) |  |  |  |  | - | + | - | - |
| M264 | 12.54 |  |  |  |  | 683.1404 | C20 H27 O19 N8 | 0.95 | 212.0016(100) | - | + | - | - |
| M265 | 12.61 | 572.2318 | C26 H38 O13 N | -3.42 | 331.1530(19) 313.1433(23) 287.1280(31) 207.1014(100) 189.0906(47) 163.0752(59) | 553.1932 | C26 H33 O13 | 1.04 | - | + | + | + | - |
| M266 | 12.64 |  |  |  |  | 535.1455 | C20 H29 O12 N3 S | -4.17 | 535.1815(22) 113.0229(100) | - | + | - | - |
| M267 | 12.71 | 347.1124 | C18 H19 O7 | -0.46 | 329.1012(15) 299.0910(53) 193.0495(36) 162.0674(10) 149.0596(52) |  |  |  |  | - | + | - | - |
| M268 | 12.72 |  |  |  |  | 363.1086 | C18 H19 O8 | 0.27 | 363.1668(2) 315.0876(39) 271.0982(6) 256.0738(13) 195.0655(39) 165.0546(100) 150.0310(15) | - | + | - | - |
| M269 | 12.75 | 317.1022 | C17 H17 O6 | 0.65 | 175.0752(82) 163.0750(29) 149.0597(48) 137.0596(26) 109.1014(99) |  |  |  |  | - | + | - | - |
| M270 | 12.94 |  |  |  |  | 780.1810 | C26 H34 O21 N7 | -0.45 | 404.1925(100) 224.1288(12) | - | + | + | - |
| M271 | 12.94 | 244.0461 | C9 H10 O7 N | 3.57 | 123.1169(47) |  |  |  |  | - | + | - | - |
| M272 | 12.95 |  |  |  |  | 567.1720 | C26 H31 O14 | 0.13 | 391.1405(9) 343.1185(50) 195.0654(27) 175.0236(19) 165.0548(11) 113.0230(100) | + | + | + | - |
| M273 | 13.07 | 331.1538 | C19 H23 O5 | -0.48 | 287.1273(21) 255.1012(42) 227.1062(43) 189.0909(46) 151.0754(100) 137.0597(68) |  |  |  |  | - | + | - | - |
| M274 | 13.08 | 457.1271 | C19 H25 O9 N2 S | -0.98 | 145.0494(1) 127.0396(2) | 455.1130 | C19 H23 O9 N2 S | -0.05 | 197.0712(16) 174.0372(15) 150.0371(22) 113.0231(7) 101.0230(10) | + | + | + | + |
| M275 | 13.08 | 235.1803 | C14 H23 O N2 | -0.81 | 235.1684(82) 130.0650(44) |  |  |  |  | - | + | - | - |
| M276 | 13.13 | 589.2603 | C26 H41 O13 N2 | -0.01 | 589.2557(14) 427.2077(26) 265.1544(100) |  |  |  |  | - | + | - | - |
| M277 | 13.15 |  |  |  |  | 597.1823 | C27 H33 O15 | -0.31 | 421.1509(28) 404.1867(5) 227.0918(6) 212.0019(18) 193.0500(56) 175.0241(19) 138.0310(28) 113.0229(100) | - | + | - | - |
| M278 | 13.17 | 272.1281 | C16 H18 O3 N | -0.15 | 272.1313(32) 255.1044(37) 191.0700(100) 177.0905(26) 149.0958(32) 135.0803(63) |  |  |  |  | - | + | - | - |
| M279 | 13.33 |  |  |  |  | 523.1819 | C25 H31 O12 | -0.42 | 523.1858(44) 347.1497(99) 329.1396(11) 317.1400(18) 113.0230(100) | - | + | - | - |
| M280 | 13.36 |  |  |  |  | 567.1726 | C26 H31 O14 | 1.10 | 391.1399(5) 343.1209(11) 195.0655(50) 165.0543(23) 113.0229(100) | - | + | + | - |
| M281 | 13.37 | 633.1594 | C17 H29 O18 N8 | -0.11 | 477.1556(1) 295.0747(27) 277.0638(87) 174.0370(100) 141.0181(15) | 631.1451 | C17 H27 O18 N8 | 0.32 | 527.0928(4) 455.1152(5) 275.0498(83) 212.0020(33) 193.0346(100) 113.0230(65) | - | + | - | - |
| M282 | 13.41 |  |  |  |  | 461.1668 | C20 H29 O12 | 0.65 | 461.1657(74) 113.0228(2) 101.0229(4) | + | + | - | - |
| M283 | 13.51 |  |  |  |  | 771.1924 | C24 H35 O21 N8 | 0.28 | 566.1783(1) 184.2137(1) | - | + | - | - |
| M284 | 13.52 |  |  |  |  | 604.1882 | C25 H34 O16 N | -0.13 | 558.1824(30) 212.0021(35) 149.0445(30) 131.0337(70) 113.0230(62) | - | + | - | - |
| M285 | 13.54 |  |  |  |  | 558.1829 | C24 H32 O14 N | 0.06 | 558.1853(10) 461.1656(3) 351.2171(11) 212.0020(13) 149.0441(22) 131.0335(45) | + | + | + | - |
| M286 | 13.65 |  |  |  |  | 387.0934 | C16 H19 O11 | 0.40 | 211.0604(100) 175.0240(6) 153.0544(40) 113.0229(66) | - | + | - | - |
| M287 | 13.66 |  |  |  |  | 567.1726 | C26 H31 O14 | 1.10 | 391.1403(9) 343.1186(16) 195.0654(49) 175.0233(16) 157.0133(26) 113.0229(100) | - | + | + | - |
| M288 | 13.67 | 216.0650 | C12 H10 O3 N | -2.45 | 216.0694(3) 144.0807(10) |  |  |  |  | - | + | - | - |
| M289 | 13.74 |  |  |  |  | 558.1829 | C24 H32 O14 N | 0.06 | 558.1821(12) 323.0979(2) 228.1235(3) 205.0710(3) 158.0601(16) 131.0335(45) | - | + | + | - |
| M290 | 13.75 | 181.0860 | C10 H13 O3 | 0.33 | 181.0857(100) 148.0518(15) |  |  |  |  | - | + | - | - |
| M291 | 13.86 | 272.1281 | C16 H18 O3 N | -0.15 | 272.1280(87) 186.0911(100) |  |  |  |  | - | + | - | + |
| M292 | 13.93 |  |  |  |  | 631.1451 | C17 H27 O18 N8 | 0.32 | 455.1143(13) 355.0871(15) 275.0495(100) 212.0021(14) 193.0346(80) | - | + | - | - |
| M293 | 13.99 | 427.2074 | C20 H31 O8 N2 | -0.29 | 427.2067(13) 265.1542(100) 201.1388(5) | 425.1931 | C20 H29 O8 N2 | 0.26 | 425.1935(22) 361.1907(4) 327.1232(19) 315.1234(57) 227.1186(69) 119.0335(48) | + | + | - | - |
| M294 | 14.17 | 414.2013 | C22 H28 O5 N3 | -2.48 | 414.2015(100) 356.1591(12) 291.1332(13) 280.1659(31) 273.1228(92) 227.1175(41) |  |  |  |  | - | + | - | + |
| M295 | 14.36 |  |  |  |  | 285.1705 | C15 H25 O5 | -0.97 | 285.1706(100) 255.1605(13) 205.0498(8) | - | + | - | + |
| M296 | 14.38 |  |  |  |  | 333.1565 | C15 H25 O8 | 3.00 | 333.1292(100) 315.1234(12) 303.1241(29) 288.0192(48) 153.0908(31) 119.0335(27) 101.0228(28) | - | + | - | - |
| M297 | 14.73 |  |  |  |  | 357.0679 | C11 H17 O13 | 1.22 | 357.0695(8) 195.0657(24) 185.0788(100) 151.0753(92) 107.0850(26) | - | + | - | - |
| M298 | 14.76 |  |  |  |  | 439.1067 | C12 H19 O12 N6 | 0.11 | 439.1072(94) 359.1497(100) 344.1262(47) | - | + | - | - |
| M299 | 14.81 | 427.2074 | C20 H31 O8 N2 | -0.29 | 427.2071(11) 265.1543(100) 247.1438(4) 201.1382(5) |  |  |  |  | + | + | - | - |
| M300 | 14.96 | 357.1807 | C20 H25 O4 N2 | -0.54 | 357.1802(100) 186.0912(19) 130.0863(13) | 355.1662 | C20 H23 O4 N2 | -0.28 | 355.1664(13) 184.0758(100) 170.0814(8) | - | + | - | + |
| M301 | 15.08 |  |  |  |  | 517.1320 | C20 H25 O14 N2 | 1.61 | 410.1386(2) 218.0491(3) 162.0218(31) 132.0474(29) | - | + | - | - |
| M302 | 15.12 | 540.2061 | C25 H34 O12 N | -2.78 | - | 521.1664 | C25 H29 O12 | -0.13 | 521.1689(10) 491.1562(7) 327.1238(53) 315.1234(69) 175.0238(23) 113.0230(100) | - | + | - | - |
| M303 | 15.15 | 501.1528 | C13 H25 O13 N8 | -1.46 | 338.1492(100) 188.0705(24) 151.0865(49) 127.1230(86) |  |  |  |  | - | + | - | - |
| M304 | 15.35 |  |  |  |  | 285.1705 | C15 H25 O5 | -0.97 | 285.1707(100) 267.1595(2) 241.1815(1) 193.1592(4) | - | + | - | + |
| M305 | 15.36 | 316.1179 | C17 H18 O5 N | -0.28 | 257.1380(9) 173.1170(6) |  |  |  |  | - | + | - | - |
| M306 | 15.40 | 500.2022 | C25 H30 O8 N3 | -1.06 | 500.2029(16) 338.1492(100) 188.0704(30) 151.0864(57) |  |  |  |  | - | + | - | - |
| M307 | 15.61 | 459.1974 | C20 H31 O10 N2 | 0.21 | 261.1261(7) 145.0495(24) 127.0389(11) | 440.1560 | C20 H26 O10 N | -0.41 | 161.0597(6) 119.0335(8) | - | + | - | - |
| M308 | 15.76 |  |  |  |  | 361.0934 | C18 H17 O8 | 1.27 | 361.0954(9) 317.1031(21) 273.1134(100) 257.0821(21) 195.0659(25) 135.0435(20) 123.0437(35) | - | - | - | + |
| M309 | 15.77 | 623.2445 | C29 H39 O13 N2 | -0.25 | 623.2412(13) 461.1902(25) 299.1386(100) |  |  |  |  | - | + | - | - |
| M310 | 15.82 | 167.0703 | C9 H11 O3 | 0.24 | 167.0700(100) |  |  |  |  | - | + | - | - |
| M311 | 15.82 | 227.0914 | C11 H15 O5 | 0.00 | 180.1379(15) 167.0701(26) 151.1116(12) 123.1169(34) 109.1014(100) |  |  |  |  | - | + | - | - |
| M312 | 15.82 | 301.1285 | C14 H21 O7 | 0.90 | 225.0755(3) 209.0807(11) 167.0701(100) |  |  |  |  | - | + | - | + |
| M313 | 15.83 |  |  |  |  | 299.1136 | C14 H19 O7 | -0.22 | 299.1133(24) 225.0762(100) 181.0859(20) 166.0624(26) | - | + | + | + |
| M314 | 15.90 | 427.2074 | C20 H31 O8 N2 | -0.29 | 427.2066(10) 265.1543(100) 247.1439(4) 201.1382(6) | 425.1931 | C20 H29 O8 N2 | 0.26 | 425.1944(24) 379.2157(10) 361.2033(10) 245.1292(13) 227.1182(100) 130.0862(22) | + | + | - | - |
| M315 | 15.99 |  |  |  |  | 423.1661 | C21 H27 O9 | -0.01 | 375.1445(33) 315.1233(14) 227.0918(23) 195.0653(38) 165.0544(100) | - | + | - | - |
| M316 | 16.00 | 383.2529 | C20 H35 O5 N2 | -3.08 | 346.2080(9) 272.1707(13) 255.1447(21) 227.1744(18) 199.1805(38) |  |  |  |  | - | + | - | - |
| M317 | 16.18 | 540.2061 | C25 H34 O12 N | -2.78 | 331.1166(11) 313.1065(100) 271.0958(24) 213.1232(12) | 521.1664 | C25 H29 O12 | -0.13 | 521.1633(2) 503.1588(2) 491.1559(4) 341.1027(100) 329.1030(92) 326.0795(22) | - | + | - | - |
| M318 | 16.24 |  |  |  |  | 379.1398 | C19 H23 O8 | -0.19 | 331.1185(47) 316.0949(14) 300.1246(17) 195.0654(43) 165.0545(100) | - | + | - | - |
| M319 | 16.40 | 304.1294 | C15 H18 O4 N3 | 0.81 | 277.1180(100) 201.1021(35) 139.0389(13) 116.0497(36) |  |  |  |  | - | + | - | - |
| M320 | 16.49 |  |  |  |  | 377.1816 | C17 H29 O9 | -0.33 | 377.1968(100) 298.0960(8) 161.0447(16) 151.0386(29) 101.0228(39) | - | + | - | - |
| M321 | 16.63 | 299.1125 | C14 H19 O7 | -0.23 | 225.0752(45) 210.0521(100) 175.0387(18) |  |  |  |  | - | + | - | - |
| M322 | 16.65 |  |  |  |  | 605.2457 | C27 H41 O15 | 2.86 | 605.2454(100) 221.0658(14) 161.0445(15) 119.0336(18) 101.0229(30) | - | + | - | - |
| M323 | 17.15 | 353.2795 | C20 H37 O3 N2 | -1.02 | 353.2795(8) 127.1231(67) |  |  |  |  | - | + | - | - |
| M324 | 17.21 |  |  |  |  | 639.2295 | C30 H39 O15 | 0.07 | 639.2303(59) 477.1778(6) 415.1777(6) 301.1444(100) 175.0236(19) 113.0230(67) | - | + | - | - |
| M325 | 17.32 |  |  |  |  | 324.0725 | C14 H14 O8 N | -0.03 | 324.0733(40) 113.0230(42) 101.0227(11) | - | + | - | - |
| M326 | 17.34 | 510.2797 | C25 H40 O8 N3 | -2.61 | 510.2837(3) 442.2755(31) 425.2499(11) 263.1395(18) 235.1438(66) |  |  |  |  | - | + | - | - |
| M327 | 17.49 | 461.1916 | C23 H29 O8 N2 | -0.61 | 461.1918(10) 299.1386(100) | 459.1774 | C23 H27 O8 N2 | 0.18 | 327.0963(8) 261.1025(97) 193.0341(9) 119.0336(55) 113.0232(59) | - | + | - | - |
| M328 | 17.59 |  |  |  |  | 597.1823 | C27 H33 O15 | -0.31 | 421.1517(10) 373.1307(33) 225.0765(40) 175.0242(23) 165.0548(24) 113.0230(100) | - | + | - | - |
| M329 | 17.83 | 327.1593 | C20 H23 O4 | 0.75 | 163.0752(85) 151.0752(15) 137.0597(100) 109.1012(17) |  |  |  |  | - | + | - | - |
| M330 | 18.18 | 387.1914 | C21 H27 O5 N2 | -0.18 | 387.1906(100) 216.1017(16) 198.0911(22) 130.0863(15) |  |  |  |  | - | + | - | - |
| M331 | 18.19 | 327.1593 | C20 H23 O4 | 0.75 | 327.1215(11) 163.0752(79) 151.0753(13) 137.0596(100) |  |  |  |  | - | + | - | - |
| M332 | 18.25 | 500.2022 | C25 H30 O8 N3 | -1.06 | 500.2022(14) 338.1493(100) 188.0704(28) 151.0865(57) |  |  |  |  | - | + | - | - |
| M333 | 18.28 |  |  |  |  | 491.1566 | C24 H27 O11 | 1.38 | 491.1530(35) 315.1232(99) 285.1134(13) 241.1231(27) 175.0234(38) 113.0230(100) | - | + | - | - |
| M334 | 18.33 |  |  |  |  | 432.1302 | C21 H22 O9 N | 0.36 | 270.1346(100) 252.1242(4) 195.1018(9) 151.1113(17) 113.0229(17) | - | + | - | - |
| M335 | 18.42 | 275.1039 | C14 H15 O4 N2 | 4.53 | 275.1036(79) 231.1138(18) 146.0447(100) 102.0547(46) |  |  |  |  | + | - | - | - |
| M336 | 18.49 | 458.2791 | C29 H36 O2 N3 | -2.39 | 243.1457(16) 226.1184(15) 185.1647(50) 173.1283(16) 129.1023(21) |  |  |  |  | - | + | - | - |
| M337 | 18.53 | 317.1383 | C18 H21 O5 | -0.13 | 299.1424(64) 271.0954(40) 197.1168(32) 179.0483(59) 162.0218(98) |  |  |  |  | - | + | - | - |
| M338 | 18.54 | 461.1916 | C23 H29 O8 N2 | -0.61 | 461.1905(6) 299.1386(100) 120.0809(10) | 459.1774 | C23 H27 O8 N2 | 0.18 | 459.1757(10) 321.1455(8) 279.1141(8) 261.1031(67) 119.0336(42) 113.0230(54) | - | + | - | - |
| M339 | 18.55 | 331.1546 | C19 H23 O5 | 1.90 | 331.1726(24) 285.1117(24) 255.1014(38) 227.1065(48) 189.0910(34) 151.0754(100) |  |  |  |  | - | + | - | - |
| M340 | 18.57 |  |  |  |  | 404.1826 | C20 H26 O6 N3 | -0.29 | 404.1787(100) 298.1406(9) 273.0880(62) 254.1509(25) 243.1234(77) 130.0860(65) | - | + | - | - |
| M341 | 18.57 | 338.1500 | C19 H20 O3 N3 | 0.21 | 338.1496(45) 209.0916(10) 188.0704(72) 151.0865(100) |  |  |  |  | - | + | - | + |
| M342 | 18.62 | 327.1224 | C19 H19 O5 | -0.89 | 327.1223(100) 309.1116(23) 285.1115(38) 203.0701(43) 137.0597(11) |  |  |  |  | - | + | - | - |
| M343 | 18.62 |  |  |  |  | 412.1622 | C19 H26 O9 N | 2.10 | 412.1612(100) 250.1079(19) 206.1178(34) 167.0338(20) 116.0702(41) 101.0228(11) | - | + | - | - |
| M344 | 18.63 | 345.1331 | C19 H21 O6 | -0.59 | 327.1223(39) 309.1118(11) 285.1112(100) 253.0853(30) 241.1430(55) |  |  |  |  | - | + | - | - |
| M345 | 18.69 |  |  |  |  | 391.1409 | C20 H23 O8 | 2.71 | 391.1433(3) 357.1484(2) 343.1184(100) 311.0924(13) 195.0654(46) 165.0545(43) | + | + | + | - |
| M346 | 18.69 | 299.1384 | C17 H19 O3 N2 | -1.97 | 299.1386(100) 283.0993(38) 235.1226(21) 223.0962(37) 187.0751(37) |  |  |  |  | - | + | - | + |
| M347 | 18.70 |  |  |  |  | 619.1632 | C24 H31 O17 N2 | 0.63 | 416.1011(12) 382.1140(24) 338.1245(22) 201.1123(100) 176.0706(68) 170.0811(90) | - | + | - | - |
| M348 | 18.78 | 483.1873 | C23 H31 O11 | 2.49 | 378.1526(100) 243.0872(46) 105.0338(21) |  |  |  |  | - | + | - | - |
| M349 | 18.85 |  |  |  |  | 421.1505 | C21 H25 O9 | 0.11 | 421.1536(6) 373.1289(52) 225.0762(52) 195.0653(31) 165.0545(100) 150.0309(28) | - | + | - | - |
| M350 | 18.94 |  |  |  |  | 460.1619 | C23 H26 O9 N | 1.36 | 460.1609(100) 298.1089(13) 254.1183(66) 164.0705(51) 150.0548(58) 147.0439(16) | - | + | - | - |
| M351 | 19.21 |  |  |  |  | 513.2702 | C26 H41 O10 | -0.63 | 513.2719(11) | - | - | + | - |

**Table S4** Predictable metabolic pathways.

| Metabolic pathway | Molecular formula change | m/z change |
| --- | --- | --- |
| D(+)-Sucrose removal | -C12H22O11 | -342.1157 |
| D-glucuronic acid removal | -C6H10O7 | -194.0421 |
| D-Galacturonic acid removal | -C6H10O7 | -194.0421 |
| D-mannose removal | -C6H12O6 | -180.0628 |
| D-glucose removal | -C6H12O6 | -180.0628 |
| D-galactose removal | -C6H12O6 | -180.0628 |
| Fructose removal | -C6H12O6 | -180.0628 |
| D-glucosamine removal | -C6H13NO5 | -179.0788 |
| L-rhamnose removal | -C6H12O5 | -164.0679 |
| D-fucose removal | -C6H12O5 | -164.0679 |
| D-lyxose removal | -C5H10O5 | -150.0523 |
| D-ribose removal | -C5H10O5 | -150.0523 |
| D-xylose removal | -C5H10O5 | -150.0523 |
| L-Arabinose removal | -C5H10O5 | -150.0523 |
| D-erythrose removal | -C4H8O4 | -120.0417 |
| Debenzylation | -C7H6 | -90.0470 |
| Debromination | -Br+H | -77.9105 |
| Trifluoromethyl loss | -CF3+H | -67.9874 |
| Oxidative debromination | +O-HBr | -63.9312 |
| Hydrolysis of nitrate esters | -NO2+H | -44.9851 |
| Decarboxylation | -CO2 | -43.9898 |
| Isopropyl dealkylation | -C3H6 | -42.0470 |
| Propyl ketone to acid | -C4H8+O | -40.0679 |
| Tert-butyl to alcohol | -C4H8+O | -40.0679 |
| Dechlorination | -Cl+H | -33.9610 |
| Hydroxymethylene loss | -CH2O | -30.0106 |
| Nitro-reduction | -O2+H2 | -29.9742 |
| Propyl ether to acid | -C3H8+O | -28.0677 |
| Tert-butyl to acid | -C3H8+O | -28.0677 |
| N-Deethylation | -C2H4 | -28.0313 |
| O-Deethylation | -C2H4 | -28.0313 |
| S-Deethylation | -C2H4 | -28.0313 |
| Tert-butyl dealkylation | -C2H4 | -28.0313 |
| Decarbonylation | -CO | -27.9949 |
| Ethyl ketone to acid | -C3H6+O | -26.0520 |
| Isopropyl to alcohol | -C3H6+O | -26.0520 |
| Oxidative dechlorination | +O-HCl | -19.9818 |
| Alcohols dehydration | -H2O | -18.0106 |
| Dehydration of oximes | -H2O | -18.0106 |
| Defluorination | -F+H | -17.9906 |
| Sulfoxide to thioether | -O | -15.9949 |
| Thioureas to ureas | -S+O | -15.9772 |
| Ethyl ether to acid | -C2H6+O | -14.0520 |
| Isopropyl to acid | -C2H6+O | -14.0520 |
| N-Demethylation | -CH2 | -14.0157 |
| O-Demethylation | -CH2 | -14.0157 |
| S-Demethylation | -CH2 | -14.0157 |
| Ethyl to alcohol | -C2H4+O | -12.0364 |
| Methyl ketone to acid | -C2H4+O | -12.0364 |
| Oxidation-De-ethylation | +O-CH3CH | -12.0364 |
| Two sequential desaturations | -H4 | -4.0313 |
| Tertiary amine oxides | -H2 | -2.0157 |
| Desaturation | -H2 | -2.0157 |
| 1,4-dihydropyridines to pyridines | -H2 | -2.0157 |
| Hydroxylation and dehydration | -H2 | -2.0157 |
| First/second alcohols to aldehyde/ketone | -H2 | -2.0157 |
| 1,4-dihydropyridines to pyridines | -H2 | -2.0157 |
| Oxidative Deamination | +O-NH3 | -1.0316 |
| 2-ethoxyl to acid | -CH4+O | -0.0364 |
| Demethylation and methylene to ketone | -CH4+O | -0.0364 |
| Demethylation and hydroxylation | -CH2+O | 1.9793 |
| Oxidation-Demethylation | +O-CH2 | 1.9793 |
| Nitroso | +H2 | 2.0157 |
| Double-bond Reduction | +H2 | 2.0157 |
| Aldehyde to Alcohol | +H2 | 2.0157 |
| Ketone to alcohol | +H2 | 2.0157 |
| Hydroxylation and desaturation | -H2+O | 13.9793 |
| Methylene to ketone | -H2+O | 13.9793 |
| Methylation (O, N, S) | +CH2 | 14.0157 |
| Ethyl to carboxylic acid | -C2H4+O2 | 15.9585 |
| Aromatic hydroxylation | +O | 15.9949 |
| Aliphatic hydroxylation | +O | 15.9949 |
| Epoxidation | +O | 15.9949 |
| N-oxidation | +O | 15.9949 |
| S-oxidation | +O | 15.9949 |
| Alkene to epoxide | +O | 15.9949 |
| Aromatic ring to arene oxide | +O | 15.9949 |
| Second/third amine to hydroxylamine/N-oxide | +O | 15.9949 |
| Thioether to sulfoxide, sulfoxide to sulfone | +O | 15.9949 |
| Demethylation and two hydroxylations | -CH2+O2 | 17.9742 |
| Hydrolysis | +H2O | 18.0106 |
| Hydrolysis of aromatic nitriles | +H2O | 18.0106 |
| Internal hydrolysis, hydration | +H2O | 18.0106 |
| Demethylation to carboxylic acid | -H2+O2 | 29.9742 |
| Hydroxylation and ketone formation | -H2+O2 | 29.9742 |
| Quinone formation | -H2+O2 | 29.9742 |
| Hydroxylation and Methylation | +CH2O | 30.0106 |
| Thioether to sulfone | +O2 | 31.9898 |
| Alkenes to dihydrodiol | +O2H2 | 34.0055 |
| Acetylation | +COCH2 | 42.0106 |
| Hydroxylation and Ethylation | +C2H4O | 44.0257 |
| Aromatic thiols to sulfonic acids | +O3 | 47.9847 |
| Glycine Conjugation (Carboxylic acids) | -OH+C2H4NO2 | 57.0215 |
| Sulfation | +SO3 | 79.9568 |
| Hydroxylation and Sulfation | +O4S | 95.9517 |
| Cysteine conjugation | +C3H5NOS | 103.0092 |
| Taurine conjugation | +C2H5NO2S | 107.0041 |
| S-cysteine conjugation | +C3H5NO2S | 119.0041 |
| De-ethlylation (O, N, S) + Glucuronidation | -CH3CH+C6H8O6 | 148.0008 |
| Decarboxylation and Glucuronidation | +C5H8O5 | 148.0372 |
| N-acetylcysteine conjugation | +C5H7NO3S | 161.0147 |
| De-methylation (O, N, S)+ Glucuronidation | -CH2+C6H8O6 | 162.0164 |
| Glucuronidation (O, N, S) | +C6H8O6 | 176.0321 |
| Glutathione conjugation (Glutamate Removal) | +C10H17N3O6S-C5H8NO3 | 177.0334 |
| Hydroxylation and Glucuronidation (O, N, S) | +C6H8O7 | 192.0270 |
| Oxidation+Glucuronidation | +O+C6H8O6 | 192.0270 |
| Desaturation + S-GSH conjugation | +C10H15N3O6S | 305.0682 |
| Glutathione conjugation | +C10H17N3O6S | 307.0838 |
| Epoxidation + S-GSH conjugation | +O+C10H17N3O6S | 323.0787 |

**Table S5** Correlation construction of CKXR-related components *in vitro* and *in vivo*.

| **No.** | **Name** |
| --- | --- |
| M1 | May be unpredictable metabolite of H164 |
| M2 | H10 |
| M3 | H11 |
| M4 | May be unpredictable metabolite of H41 |
| M5 | H34-C12H22O11 or H36-C6H12O6 |
| M6 | May be unpredictable metabolite of H25 or H30 |
| M7 | May be unpredictable metabolite of H40 |
| M8 | - |
| M9 | May be unpredictable metabolite of H41 |
| M10 | H11+C5H8O5+CH2 |
| M11 | H11-O or H116-NO2+H |
| M12 | H2-C2H4+O2 |
| M13 | H174+C10H17N3O6S+SO3 |
| M14 | H12+O-NH3-C2H4 |
| M15 | May be unpredictable metabolite of H8 or H122 |
| M16 | - |
| M17 | H36-CO or H97+H2O-CO |
| M18 | H97+C2H4O |
| M19 | H38-NO2+H-C6H12O6 |
| M20 | H44-OH+C2H4NO2-H2O or H45-OH+C2H4NO2-C6H12O6 or H51-OH+C2H4NO2-H2O or H59-OH+C2H4NO2-C6H12O6 |
| M21 | May be unpredictable metabolite of H41 |
| M22 | - |
| M23 | - |
| M24 | - |
| M25 | - |
| M26 | - |
| M27 | H11-OH+C2H4NO2 |
| M28 | H18 |
| M29 | H19 |
| M30 | H20 |
| M31 | H21 |
| M32 | H20-C2H4 |
| M33 | - |
| M34 | - |
| M35 | - |
| M36 | May be unpredictable metabolite of H34 |
| M37 | May be unpredictable metabolite of H111 or H139 |
| M38 | H34-H2+O or H36+C6H8O6 |
| M39 | H25 |
| M40 | H25+O or H30+O |
| M41 | H27 |
| M42 | - |
| M43 | H29 |
| M44 | - |
| M45 | - |
| M46 | - |
| M47 | - |
| M48 | - |
| M49 | - |
| M50 | - |
| M51 | May be unpredictable metabolite of H2 |
| M52 | - |
| M53 | Isomer of H27 |
| M54 | - |
| M55 | H25+CH2O or H30+CH2O |
| M56 | Isomer of H29 |
| M57 | H30 |
| M58 | May be unpredictable metabolite of H161 |
| M59 | H44-CH2-NO2+H or H51-CH2-NO2+H or H75+H2-C6H13NO5 |
| M60 | H76-C6H12O6-O or H88-O-H2O |
| M61 | H42-C12H22O11 |
| M62 | - |
| M63 | - |
| M64 | May be unpredictable metabolite of H39 or H65 |
| M65 | May be unpredictable metabolite of H141 |
| M66 | May be unpredictable metabolite of H164 |
| M67 | H11-CH2+C6H8O6 |
| M68 | H82-CH2+O or H109-CH2+O |
| M69 | May be unpredictable metabolite of H161 |
| M70 | H14+C6H8O7-CH4+O or H53-C3H6+O-C7H6 |
| M71 | May be unpredictable metabolite of H157 |
| M72 | May be unpredictable metabolite of H118 or H138 |
| M73 | May be unpredictable metabolite of H161 |
| M74 | - |
| M75 | H49-C12H22O11+H2O |
| M76 | H32-C6H10O5 or H43-C6H10O5 |
| M77 | H33 |
| M78 | H34 |
| M79 | H36 |
| M80 | H85+H2O |
| M81 | - |
| M82 | - |
| M83 | H94+O-NH3-C3H6+O |
| M84 | - |
| M85 | H99-C6H12O6 or H102-C6H12O6 or H111-C6H12O6 |
| M86 | - |
| M87 | May be unpredictable metabolite of H161 |
| M88 | - |
| M89 | H44+H2O or H51+H2O or H81+O or H82-C2H4 or H109-C2H4 |
| M90 | - |
| M91 | H44 |
| M92 | H97+O |
| M93 | - |
| M94 | - |
| M95 | H88-CH2 or H97+C5H8O5 |
| M96 | - |
| M97 | - |
| M98 | - |
| M99 | H42-C6H10O5 |
| M100 | H49 |
| M101 | - |
| M102 | H51 |
| M103 | H97+O |
| M104 | May be unpredictable metabolite of H12 |
| M105 | H69+C3H5NO2S+C3H5NO2S or H93+C3H5NO2S+C3H5NO2S or H122+O2H2+O2H2 |
| M106 | - |
| M107 | H83-C6H10O7+C2H5NO2S |
| M108 | H44-CO or H51-CO or H81-CH2O or H99-C6H10O5 or H102-C6H10O5 |
| M109 | H49-C6H10O5 |
| M110 | - |
| M111 | H12-O和-O2+H2 |
| M112 | May be unpredictable metabolite of M115 |
| M113 | - |
| M114 | H99-C6H12O6 or H102-C6H12O6 or H111-C6H12O6 |
| M115 | H11+COCH2 |
| M116 | - |
| M117 | - |
| M118 | - |
| M119 | H37-O2+H2-C5H10O5 |
| M120 | - |
| M121 | May be unpredictable metabolite of H8 |
| M122 | - |
| M123 | - |
| M124 | - |
| M125 | H39+CH2O-O2+H2 |
| M126 | - |
| M127 | - |
| M128 | H163+C5H8O5-OH+C2H4NO2 |
| M129 | H59 |
| M130 | May be unpredictable metabolite of H12 |
| M131 | - |
| M132 | H44-CO or H51-CO or H81-CH2O or H99-C6H10O5 or H102-C6H10O5 |
| M133 | May be unpredictable metabolite of H12 or H41 |
| M134 | - |
| M135 | H81+CH2O or H82-CH2 or H107-C6H10O5 or H109-CH2 |
| M136 | M115+C5H8O5+CH2 |
| M137 | - |
| M138 | H99-CH2 or H102-CH2 or H112-C2H4 |
| M139 | - |
| M140 | May be unpredictable metabolite of H157 or H163 |
| M141 | - |
| M142 | - |
| M143 | H97-CH2O |
| M144 | H25-H2 or H30-H2 or H34-C6H12O5 or H97+O |
| M145 | - |
| M146 | May be unpredictable metabolite of H97 |
| M147 | H65 |
| M148 | H65+C10H17N3O6S-C2H4 |
| M149 | - |
| M150 | - |
| M151 | - |
| M152 | H81+CH2O or H82-CH2 or H107-C6H10O5 or H109-CH2 |
| M153 | - |
| M154 | - |
| M155 | May be unpredictable metabolite of H133 |
| M156 | - |
| M157 | Isomer of H64 |
| M158 | - |
| M159 | - |
| M160 | - |
| M161 | May be unpredictable metabolite of H12 |
| M162 | H81+CH2O or H82-CH2 or H107-C6H10O5 or H109-CH2 |
| M163 | M177+C3H5NO2S-C2H6+O |
| M164 | May be unpredictable metabolite of H118 or H138 |
| M165 | H113+C3H5NOS-H2O or H127+C5H7NO3S-C4H8O4 or H132+C5H7NO3S-C6H12O5 |
| M166 | H68 |
| M167 | May be unpredictable metabolite of H12 |
| M168 | H11+COCH2-O |
| M169 | - |
| M170 | - |
| M171 | H72 |
| M172 | - |
| M173 | H75 |
| M174 | H65-H4 |
| M175 | H76 |
| M176 | Isomer of H119 ＆ H125 |
| M177 | H44-CO2 or H51-CO2 or H75-C6H13NO5 |
| M178 | - |
| M179 | - |
| M180 | H81 |
| M181 | H82 |
| M182 | - |
| M183 | - |
| M184 | H25+COCH2 or H30+COCH2 or H34-C4H8O4 or H48-C6H10O5 |
| M185 | - |
| M186 | H83-C6H10O5 |
| M187 | Isomer of H72 ＆ H80 |
| M188 | H11-H2 |
| M189 | H40+CH2+CH2 |
| M190 | May be unpredictable metabolite of H12 |
| M191 | H85 |
| M192 | - |
| M193 | May be unpredictable metabolite of H118 or H138 |
| M194 | H75+C2H4O |
| M195 | - |
| M196 | M212+CH2O+O |
| M197 | H88 |
| M198 | H129-C5H10O5-C6H10O7 |
| M199 | Isomer of H18 |
| M200 | - |
| M201 | - |
| M202 | May be unpredictable metabolite of H121 |
| M203 | H25-CH2O or H30-CH2O |
| M204 | Isomer of H75 |
| M205 | - |
| M206 | H31-C6H10O7+H2 |
| M207 | H91-C6H12O5 or H94-C6H12O5 |
| M208 | H31+H2O-C12H22O11 or H57-CO-O2+H2 |
| M209 | H95 |
| M210 | - |
| M211 | H115-CO2-CO2 |
| M212 | - |
| M213 | H53+COCH2 or H55+COCH2 or H95-H2+O |
| M214 | H45-CH2O or H59-CH2O or H99-H2 or H102-H2 |
| M215 | H97 |
| M216 | - |
| M217 | - |
| M218 | - |
| M219 | - |
| M220 | H76-CH2+O2-C4H8+O |
| M221 | H48-O-C6H10O5 or H101+H2-C6H10O5 |
| M222 | H76-C12H22O11 or H88-C6H12O6 or H97-H2O |
| M223 | H124-CH2+O |
| M224 | M238+C5H8O5+CH2 or M260+C5H8O5+CH2 |
| M225 | H100 |
| M226 | H164-C2H6+O |
| M227 | - |
| M228 | H97-H2+O |
| M229 | - |
| M230 | H177+O3+O2H2 |
| M231 | H45+C3H5NOS or H59+C3H5NOS or H88+C5H7NO3S |
| M232 | H102 |
| M233 | H123-C6H10O7-C7H6 |
| M234 | H11+COCH2 |
| M235 | May be unpredictable metabolite of H12 |
| M236 | Isomer of H82 or H109 |
| M237 | H15+C5H8O5 or H44-CH4+O or H51-CH4+O |
| M238 | H39+C2H4O+H2 |
| M239 | May be unpredictable metabolite of H38 or H91 |
| M240 | - |
| M241 | H97-H4 |
| M242 | - |
| M243 | H94+COCH2 |
| M244 | H88-CH2O |
| M245 | - |
| M246 | May be unpredictable metabolite of H8 |
| M247 | H137-CH4+O |
| M248 | H108 |
| M249 | H109 |
| M250 | - |
| M251 | - |
| M252 | H44-C2H4+O or H51-C2H4+O |
| M253 | - |
| M254 | H88-H2+O or H97+C6H8O6 |
| M255 | H111 |
| M256 | H112 |
| M257 | - |
| M258 | M135-H2O |
| M259 | - |
| M260 | H39+C2H4O和+H2 |
| M261 | H118+H2-C4H8+O |
| M262 | H44+C3H5NOS or H51+C3H5NOS or H97+C5H7NO3S |
| M263 | - |
| M264 | - |
| M265 | - |
| M266 | H25+C10H17N3O6S-C5H8NO3 or H30+C10H17N3O6S-C5H8NO3 |
| M267 | - |
| M268 | H139-C2H6+O or H143-C2H4 |
| M269 | H118-C2H6+O |
| M270 | - |
| M271 | H8+O2-H2+O2 |
| M272 | H124-H2+O or H143+C6H8O6 |
| M273 | H118 |
| M274 | H44+C3H5NOS or H51+C3H5NOS or H97+C5H7NO3S |
| M275 | H11-NO2+H |
| M276 | H121 |
| M277 | H52-CH2O |
| M278 | H185-H4-C2H6+O |
| M279 | H137-CH2+O |
| M280 | H124-H2+O or H143+C6H8O6 |
| M281 | M262+C6H8O6 |
| M282 | Isomer of H112 |
| M283 | - |
| M284 | H95-CH2 or H108-CH2O |
| M285 | - |
| M286 | - |
| M287 | H124-H2+O or H143+C6H8O6 |
| M288 | H65+O-NH3 |
| M289 | - |
| M290 | H126 |
| M291 | - |
| M292 | M262+C6H8O6 |
| M293 | H121-C6H10O5 |
| M294 | May be unpredictable metabolite of H12 |
| M295 | H166+H2O+H2O or H192-H2+O2-CH4+O |
| M296 | H151-C5H10O5 |
| M297 | - |
| M298 | - |
| M299 | H121-C6H10O5 |
| M300 | May be unpredictable metabolite of H11 |
| M301 | May be unpredictable metabolite of H105 |
| M302 | H137-CH4+O |
| M303 | - |
| M304 | H172+O2H2-C3H6 |
| M305 | - |
| M306 | May be unpredictable metabolite of H38 |
| M307 | H88-O |
| M308 | - |
| M309 | H130 |
| M310 | - |
| M311 | H118-CH2-C7H6 or H138-CH2-C7H6 |
| M312 | - |
| M313 | - |
| M314 | H121-C6H10O5 |
| M315 | H119-C6H10O5 or H125-C6H10O5 or H153+O |
| M316 | - |
| M317 | H137-CH4+O |
| M318 | H139-CH2+O or H153-C2H4 |
| M319 | - |
| M320 | H151+CH2-C4H8O4 |
| M321 | May be unpredictable metabolite of H122 |
| M322 | H131 |
| M323 | - |
| M324 | H129-C4H8+O |
| M325 | - |
| M326 | - |
| M327 | H130-C6H10O5 |
| M328 | H52-CH2O |
| M329 | H23-C6H10O7+C3H5NO2S |
| M330 | - |
| M331 | H23-C6H10O7+C3H5NO2S |
| M332 | May be unpredictable metabolite of H38 |
| M333 | - |
| M334 | - |
| M335 | H40-OH+C2H4NO2和+CH2O |
| M336 | - |
| M337 | - |
| M338 | H130-C6H10O5 |
| M339 | H138 |
| M340 | - |
| M341 | - |
| M342 | H118-H4 or H138-H4 |
| M343 | H48+C2H4O-C6H10O7 |
| M344 | H138-H2+O |
| M345 | H143 |
| M346 | H130-C12H22O11+H2O |
| M347 | H48-OH+C2H4NO2-CH4+O or H101-OH+C2H4NO2-CH2+O2 |
| M348 | - |
| M349 | H119-C6H12O5 or H125-C6H12O5 or H143+CH2O or H153-H2+O |
| M350 | H153-OH+C2H4NO2-H4 |
| M351 | - |
